# New Sorbicillinoids with Tea Pathogenic Fungus Inhibitory Effect from Marine-Derived Fungus *Hypocrea jecorina* H8

**DOI:** 10.3390/md20030213

**Published:** 2022-03-17

**Authors:** Shun-Zhi Liu, Guang-Xin Xu, Feng-Ming He, Wei-Bo Zhang, Zhen Wu, Ming-Yu Li, Xi-Xiang Tang, Ying-Kun Qiu

**Affiliations:** 1 State Key Laboratory of Cellular Stress Biology, School of Pharmaceutical Sciences, Xiamen University, South Xiang-An Road, Xiamen 361102, China; lsz@xmu.edu.cn (S.-Z.L.); fengminghe@stu.xmu.edu.cn (F.-M.H.); limingyu@xmu.edu.cn (M.-Y.L.); 2 Key Laboratory of Marine Biogenetic Resources, Third Institute of Oceanography State, Ministry of Natural Resources, Da-Xue Road, Xiamen 361005, China; xuguangxin@tio.org.cn (G.-X.X.); wuzhen@xmu.edu.cn (Z.W.); 3 State Key Laboratory of Marine Life, Ocean University of China, Yu-Shan Road, Qingdao 266100, China; 21200631103@stu.ouc.edu.cn

**Keywords:** *Hypocrea jecorina* H8, trichodermolide C, D, tea pathogenic fungus inhibitory effect, toxicity assessment

## Abstract

Four new dimeric sorbicillinoids (**1**–**3** and **5**) and a new monomeric sorbicillinoid (**4**) as well as six known analogs (**6**–**11**) were purified from the fungal strain *Hypocrea jecorina* H8, which was obtained from mangrove sediment, and showed potent inhibitory activity against the tea pathogenic fungus *Pestalotiopsis theae* (*P. theae*). The planar structures of **1**–**5** were assigned by analyses of their UV, IR, HR-ESI-MS, and NMR spectroscopic data. All the compounds were evaluated for growth inhibition of tea pathogenic fungus *P. theae*. Compounds **5**, **6**, **8**, **9**, and **10** exhibited more potent inhibitory activities compared with the positive control hexaconazole with an ED_50_ of 24.25 ± 1.57 µg/mL. The ED_50_ values of compounds **5**, **6**, **8**, **9**, and **10** were 9.13 ± 1.25, 2.04 ± 1.24, 18.22 ± 1.29, 1.83 ± 1.37, and 4.68 ± 1.44 µg/mL, respectively. Additionally, the effects of these compounds on zebrafish embryo development were also evaluated. Except for compounds **5** and **8**, which imparted toxic effects on zebrafish even at 0.625 μM, the other isolated compounds did not exhibit significant toxicity to zebrafish eggs, embryos, or larvae. Taken together, sorbicillinoid derivatives (**6**, **9**, and **10**) from *H. jecorina* H8 displayed low toxicity and high anti-tea pathogenic fungus potential.

## 1. Introduction

The influence of bioactive compounds from natural sources on human life has challenged scientists to research new environmental contexts and the associated biological diversity [1]. The ocean, as the largest frontier in biological exploration, represents one of the most favorable reservoirs of organisms producing secondary metabolites with biological activities [2]. The deep sea is an extreme environment; in this respect, its associated micro-organisms have great potential to produce natural products with novel biological properties [3].

The tea plant (*Camellia sinensis* L.) is an important commercial crop all over the world. However, the tea plant suffers from biotic stresses of some pathogenic fungi [4,5], which often exhibits severe damage of the blade tissue and discoloration of the leaves, common symptoms, including blight (*Exobasidium vexans* Massee), brown blight (*Colletotrichum camelliae* Massee), and red rust (*Cephaleuros parasiticus* Karst). These pathogenic fungi greatly reduce the quality of tea and damage human health [6,7,8].

As part of our continuing exploration for structurally novel and biologically interesting secondary metabolites from marine microorganisms, the fungal strain *Hypocrea jecorina* H8 (*H. jecorina* H8) was isolated from mangrove sediments and showed potent inhibitory activity against tea pathogenic fungus *P. theae*.

Chemical investigation of *H. jecorina* H8 from rice medium led to the isolation of 11 compounds, including five sorbicillinoids (**1**–**5**) and six known sorbicillinoid analogs. Some of these compounds exhibited significant inhibitory activity against tea pathogenic fungus *P. theae*. and low toxicity to zebrafish. Herein, we report the isolation, structural determination, as well as antifungal activity of these isolated compounds.

## 2. Results

### 2.1. Structural Elucidation of New Compounds

**Figure 1:**
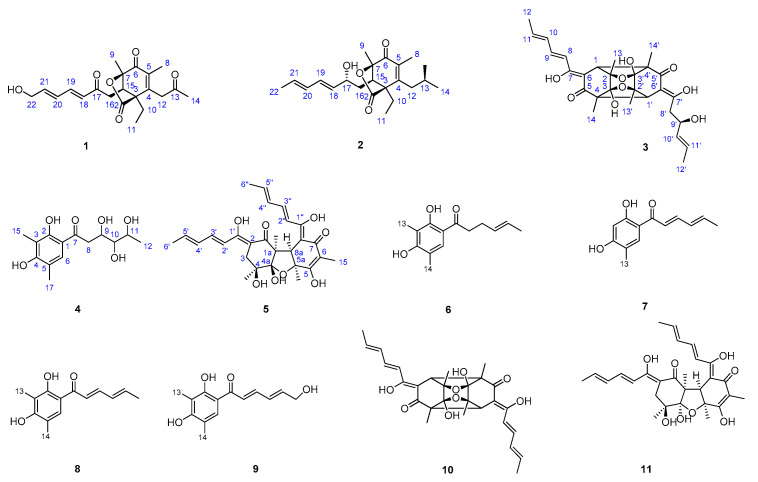
Structures of compounds **1**–**11** isolated from an extract of *Hypocrea jecorina* H8; the relative configuration of **5** is reported in this article.

A series of column chromatography (CC) methods were used during the isolation of *H. jecorina* H8. As a result, 11 compounds were isolated, including five new compounds, trichodermolide C (**1**), trichodermolide D (**2**), 7,7′,9′-hydroxy-trichodimerol (**3**), 1-(2,4-dihydroxy-3,5-dimethylphenyl)-3,4,5-trihydroxyhexan-1-one (**4**), and isobisvertinol A (**5**). At the same time, by comparing NMR spectral data with those published in literatures, the six known compounds were determined to be 2′,3′-dihydrosorbicillin (**6**) [9], 6-demethylsorbicillin (**7**) [10], sorbicillin (**8**) [9], (2*E*,4*E*)-1-(2,4-dihydroxy-3,5-dimethylphenyl)-6-hydroxyhexa-2,4-dien-1-one (**9**), trichodimerol (**10**), and bisvertinol (**11**) [11] (Figure 1).

Compound **1** was obtained as yellow amorphous powder. The molecular formula of **1** was established as C_21_H_26_O_6_ based on the HR-ESI-MS peak at *m/z* 375.1798 [M + H]^+^ (calcd for 375.1729 C_21_H_27_O_6_^+^), requiring nine degrees of unsaturation. The IR spectrum of **1** indicated the presence conjugated lactone carbonyl signal of at 1691 cm^−1^ [6]. In high chemical shifts region of ^1^H NMR, four olefinic protons were observed at *δ*_H_ 6.20 (d,15.4 Hz, H-20), 6.35 (dt, 15.2, 4.8 Hz, H-21), 6.43 (dd, 15.4, 10.2 Hz, H-18), and 7.25 (dd, 15.2, 10.8 Hz, H-19) (Table 1).

Their corresponding olefinic carbon signals were found in the *sp*^2^ region of the ^13^C NMR spectrum at *δ*_C_ 128.8 (C-20), 143.2 (C-21), 127.9 (C-18), and 143.0 (C-19). The *sp*^3^ low chemical shifts region of the ^1^H NMR spectrum displayed four notable methyl proton signals at *δ*_H_ 1.48 (9-CH_3_), 1.77 (8-CH_3_), 2.27(14-CH_3_) linked to quaternary carbons, and *δ*_H_ 0.95 (t, 7.2 Hz, 11-CH_3_) linked to a secondary carbon. 

The ^13^C NMR data showed four carbonyl signals at *δ*_C_ 204.0 (C-13), 196.5 (C-17), 191.3 (C-6), and 174.9 (C-2) attributing to a ketone carbonyl, two conjugated ketone carbonyl, and ester carbonyl, respectively. In addition, the ^13^C NMR spectrum and DEPT data of **1** showed the presence of 21 carbons, sorted into four methyls, four methylenes, five methines (four olefinic carbons), and eight quaternary carbons (four carbonyl carbons and two olefinic carbons). 

**Figure 2:**
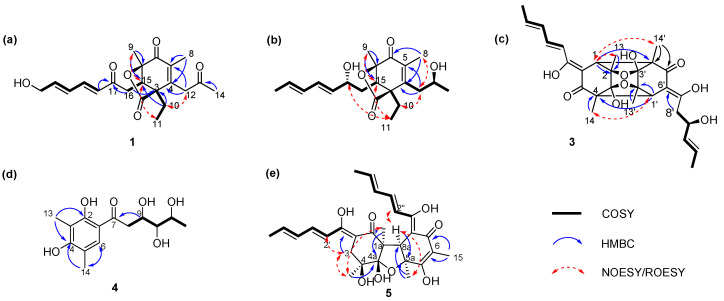
Key ^1^H–^1^H COSY, HMBC, and NOESY correlations of compounds **1**–**5**. (**a**) Key COSY, HMBC, and NOESY correlations of compounds **1**; (**b**) Key COSY, HMBC, and NOESY correlations of compounds **2**; (**c**) Key COSY, HMBC, and NOESY correlations of compounds **3**; (**d**) Key COSY, HMBC correlations of compounds **4**; (**e**) Key COSY, HMBC, and NOESY correlations of compounds **5**.

The above spectroscopic data showed high similarities to those of trichodermolide B, a known compound isolated from *Trichoderma reesei* (HN-2016-018) [12], except for the presence of signal for a hydroxyl group, a signal for methylene group at C-22 (*δ*_H_ 4.32, *δ*_C_ 62.7) and the lack of signal for a methyl group (*δ*_C_ 19.1). Thus, **1** was deduced to be a hydroxylated derivative of trichodermolide B at C-22, validated by the COSY correlations of *δ*_H_ 6.35 (H-21) with *δ*_H_ 4.32 (H2-22) (Figure 2a).

The two double bonds in sorbyl side chain for **1** were assigned both as *E* configuration based on their coupling constants (*J*_H-18/H-19_ = 15.2 Hz, *J*_H-20/H-21_ = 15.4 Hz) and the NOESY correlation between H-18/H-20. For the bridged bicycle lactone ring system, it was only possible if the CH_3_-9 and CH_2_-10 were oriented equatorially. In addition, the ^1^H NMR chemical shifts of H-15 (*δ*_H_ 3.37 for trichodermolide B compared to *δ*_H_ 3.55 for **1**), H-16 (*δ*_H_ 2.54, 2.99 for trichodermolide B compared to *δ*_H_ 2.43, 3.13 for **1**), CH_3_-9 (*δ*_H_ 1.37 for trichodermolide B compared to *δ*_H_ 1.48 for **1**), and CH_2_-10 (*δ*_H_ 1.25, 2.03 for trichodermolide B compared to *δ*_H_ 1.30, 2.08 for **1**) suggested the same relative configurations of C-3, C-15, and C-7 in 1 as that in trichodermolide B [12]. 

**Figure 3:**
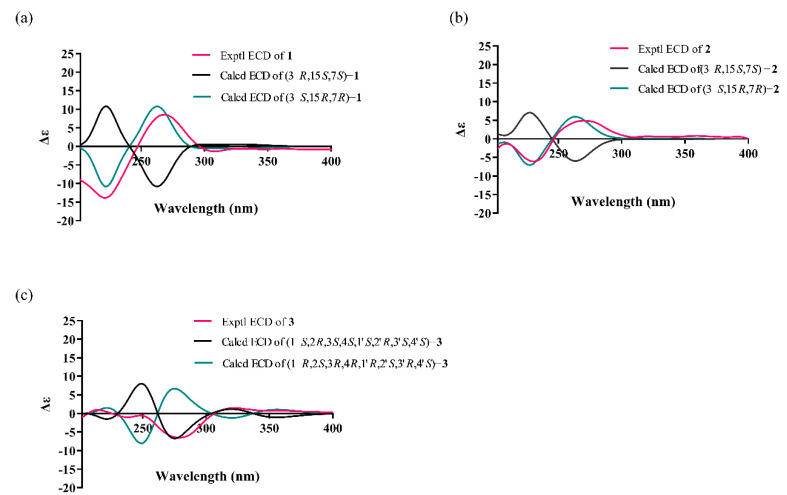
Calculated and experimental ECD spectra of compounds **1**, **2**, and **3**. (**a**) ECD spectra for compound **1**; (**b**) ECD spectra for compound **2**; (**c**): ECD spectra for compound **3**.

Therefore, the relative configuration of **1** was assumed as 3S*,15R*,7R*. The ECD curve of **1** showed a negative Cotton effect around 220 nm and a positive Cotton effect around 270 nm, respectively. These were the same as trichodermolide B (Figure S41: Calculated and experimental ECD spectra of trichodermolide B). The absolute configuration of **1** was assigned as 3*S*,15*R*,7*R* (Figure 3a). As a result, the structure of **1** was determined and named as trichodermolide C (Figure 1).

Compound **2** was also obtained as yellow amorphous powder. The molecular formula was deduced to be C_21_H_30_O_5_ by interpretation of the HR-ESI-MS peak at *m/z* 363.2162 [M + H]^+^ (calcd for 363.2093 C_21_H_31_O_5_^+^), implying seven degrees of unsaturation. Compound **2** presented ^1^H and ^13^C NMR signals similar to those of compounds **1**, especially those on the bridged bicyclic ring moiety. The structural differences in the side chains could be revealed by the DEPT spectra, in which two carbonyl signals vanished and two oxygenated methine signals emerged, together with the signal different from the oxygenated methylene (C-22) to methyl. 

The corresponding ^1^H NMR signals in **2** were found at *δ*_H_ 4.17 (H-17), 4.10 (H-13), and 1.76 (CH_3_-22), respectively. Thus, compound **2** is considered to be a reduction product of compound **1**. On the aids of ^1^H-^1^H COSY spectrum, two continuous connected spin systems revealed the presence of two side chain as: CH_3_-CH=CH-CH=CH-CH(O-)-CH_2_- and -CH_2_-CH(O)-CH_2_-. The key HMBC correlations from *δ*_H_ 2.60 (CH_2_-12) to *δ*_C_ 133.8 (C-5), 152.1 (C-4), 51.3 (C-3) and from *δ*_H_ 1.87 (CH_3_-8) to *δ*_C_ 191.9 (C-6), C-4, C-5 allowed the elucidation of structure for compound **2**. 

The *E* configurations among the two double bonds in the side chains of **2** could be confirmed by the large coupling constant (*J*_H-18/H-19_ = *J*_H-20/H-21_ = 15.2 Hz). The relative configurations of three stereocenters, C-3, C-15, and C-7 of **2** were confirmed by comparison of its ^1^H NMR data with compound **1**. The NOESY correlations between H-15 and CH_3_-11, H-17/CH_3_-11, and CH_3_-8/CH_2_-12 established the relative configuration of **2**. Therefore, the relative configuration of **2** was assumed as 3S*,15R*,7R*. The calculated ECD curve of 3*S*,15*R*,7*R*-**2** was consistent with the experimental data (Figure 3b), and hence the absolute configuration of **2** was assigned as 3*S*,15*R*,7*R*.

Compound **3** was obtained as a yellow amorphous powder. The results from the HR-ESI-MS peak at *m/z* 537.2090 [M + Na]^+^ (calcd for 537.2100 C_28_H_34_O_9_Na^+^) suggested that the molecular formula of **3** was C_28_H_34_O_9_, thus, implying twelve degrees of unsaturation. The ^1^H and ^13^C NMR data of **3** showed 30 protons and 28 carbons signals, and these carbon signals were classified into twelve quaternary carbons (four ethylenic bonds), two carbonyls, six ethylenic bonds, one oxygenated methines, two methines without an oxygen link, one methylene, and six methyls. 

Comparison of the NMR data of **3** with those of **10**, a known metabolite isolated from a strain of the same genus [6], indicated that **3** possessed an identical trichodimerol [13] skeleton to **10**. Although compound **10** afforded only 14 signals because of a symmetric structure, some ^13^C signals of compound 3 split, suggesting an asymmetric structure (Table 2). The major difference were found at the signals due to C-8′, C-10′ moiety, suggesting a hydration at C-8′/C-9′ double bond in compound **3**. In ^13^C NMR and DEPT, the double bond signals of C-8′ and C-9′ in **10** turn to a methylene (*δ*_C_ 40.9, C-8′) and an oxygen-linked methine (*δ*_C_ 80.7, C-9′) in **3**. The conclusion was confirmed by the COSY correlation from *δ*_H_ 2.38 (H-8′) to *δ*_H_ 4.31 (H-9′), and to *δ*_H_ 5.67 (H-10′). In the HMBC spectrum of **3**, the correlations from H-8′ to C-7′, C-9′ were also found.

The NOESY correlations between *δ*_H_ 1.41 (CH_3_-14) and *δ*_H_ 3.31 (H-1′) indicated the same orientation of these signals. In addition, the protons *δ*_H_ 1.41 (CH_3_-14′) and *δ*_H_ 2.94 (H-1) were simultaneously correlated with *δ*_H_ 1.35 (CH_3_-13), reflecting that these signals locate at the same orientation. (Figure 2c). The absolute configuration of **3** was established as 1*S*,2*R*,3*S*, 4*S*, 1′*S*,2′*R*, 3′*S*, 4′*S* by comparison on experimental and calculated ECD spectra (Figure 3c).

Compound **4** was isolated as a colorless amorphous powder. The molecular formula of C_14_H_20_O_6_, which gave five unsaturation degrees, was established by the positive HR-ESI-MS ion peak at *m/z* 285.1342 [M + H]^+^ (calcd for 285.1338 C_14_H_21_O_6_^+^). The UV maximum absorption bands at *λ*max (log *ε*): 216 (3.66) nm were assigned to a conjugated carbonyl, which was confirmed by the ^13^C NMR data at *δ*_C_ 192.1 (C-1′). In the ^1^H NMR, three methyl peaks at *δ*_H_ 1.19 (d, 6.0 Hz, CH_3_-12), 2.04 (s, CH_3_-13), and 2.12 (s, CH_3_-14) were assigned. One olefinic proton was also observed at 7.34 (H-6). 

In the ^13^C NMR, except for the carbonyl, six *sp*^2^ carbons at *δ*_C_ 160.3 (C-4), 159.9 (C-2), 125.1 (C-6), 118.4 (C-5), 113.6 (C-1), and 111.7 (C-3) and three oxygenated methines at *δ*_C_ 77.1 (C-3′), 76.8 (C-3′), and 65.9 (C-5′) were assigned, respectively. The benzene ring signals in compound **4** closely resembled those of the known compound 2′,3′-dihydrosorbicillin (**6**) [14]; however, they were different regarding the side chain. The COSY correlation data suggested the side chain of **4** was -CH_2_-CH(OH)-CH(OH)-CH(OH)-CH_3_. In addition, the HMBC correlation from *δ*_H_ 2.89 (CH_2_-8) to *δ*_C_ 192.1 (C-7) indicated that the carbonyl was at C-7. Comprehensive HSQC, COSY, and HMBC established the structure of **4** (Figure 1).

Compound **5** was obtained as a white amorphous powder with positive HR-ESI-MS ion peaks at m/z 499.2312 [M + H]^+^ indicating 12 degrees of unsaturation. According to the HR-ESI-MS data, compound **5** and **11** shared the same molecular formula (Figure S42). The NMR data of **5** had similar features compared to those of **11**, which suggests that they are stereoisomers. The planar structure of **5** was determined by the COSY and HMBC data. The main differences of NMR signals were attributable to C-1, C-4, and C-4a, with the ^13^C NMR deference of [*δ*_C_ 191.8 (C-1), 73.9 (C-4), and 110.2 (C-4a) in **5** vs. *δ*_C_ 194.3 (C-1), 72.7 (C-4), and 108.4 (C-4a) in **11**]. 

Thus, compounds **5** and **11** should be epimers around either C-4 or C-4a. In the NOESY spectrum, key cross peaks were observed between *δ*_H_ 3.63 (H-8a) and *δ*_H_ 1.34 (CH_3_-1a) and *δ*_H_ 1.48 (CH_3_-5a) (Figure 2e), indicating that the methyls CH_3_-1a and CH_3_-5a were at the same side with H-8a. Furthermore, NOESY were observed between CH_3_-1a and *δ*_H_ 1.29 (CH_3_-4). These results indicated that the relative configuration of C-4 was same as that of **11**. Therefore, we concluded that **5** is a stereoisomer of **11** on C-4a. The relative configuration of **5** was defined as shown in Figure 1.

### 2.2. Evaluation of Antifungal Activity

Compounds **1** to **11** were evaluated for antifungal activities by a paper disc inhibition assay. Compounds **5**, **6**, **8**, **9**, and **10** possessed significant activities against the tea pathogenic fungus *P. theae* (Table 3). The ED_50_ values of **5**, **6**, **8**, **9**, and **10** were 9.13 ± 1.25, 2.04 ± 1.91, 18.22 ± 1.29, 1.83 ± 1.37, and 4.68 ± 1.44 µg/mL, respectively. Compared to positive control hexaconazole (24.25 ± 1.57 µg/mL), compounds **5**, **6**, **8**, **9**, and **10** exhibited more potent antifungal activity. Particularly, new compound **5** had nearly 3-fold more, and known compound **9** had a 13-fold stronger anti-tea pathogenic fungus effect.

### 2.3. Evaluation of Toxicity

The zebrafish is a small teleost that is becoming increasingly popular in many biomedical and environmental studies [15]. This model has shown sensitivity to a broad variety of contaminants (such as endocrine disruptors and organic pollutants), indicating their suitability as a biological method for environmental monitoring in risk assessment.

**Figure 4:**
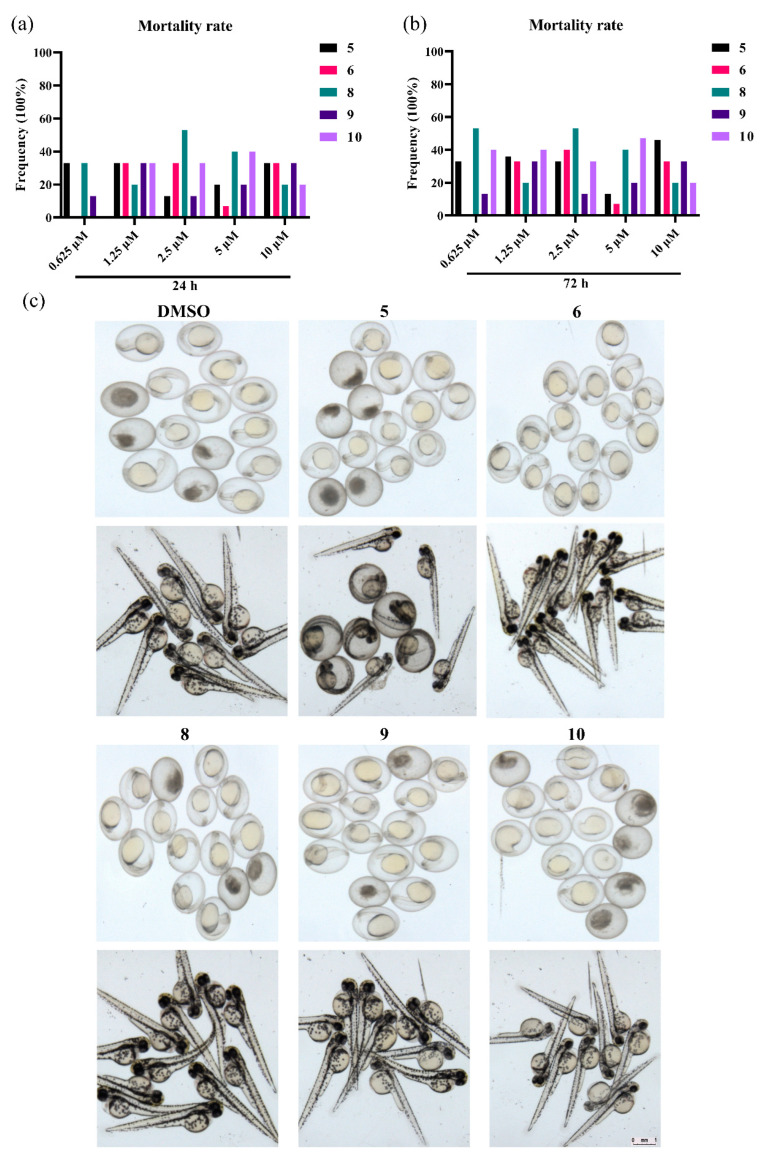
Embryotoxicity and developmental toxicity assay; 15 zebrafish embryos per condition were exposed to compounds at the concentrations of 10, 5, 2.5, 1.25, and 0.625 μM, and 0.1% DMSO was used as blank control. (**a**) The mortality rate of 24 embryo treated with compounds; (**b**) The mortality rate of 72 embryo treated with compounds; (**c**) The impact on the malformation of zebrafish treated with compounds) The statistics of 24 and 72 h mortality rate; © Morphology of 24 h embryo or 72 h zebrafish larvae treated with compounds or control.

We evaluated the toxicity of compounds **5**, **6**, **8**, **9**, and **10** in a zebrafish model (Figure 4). Figure 4a showed that these compounds, except compound **8**, killed zebrafish embryo less than 50% when treated with a concentration of 10 μM. When the treatment time was prolonged to 72 h (Figure 4b), the mortality rate of zebrafish embryo caused by compound **8** at 0.625 μM increased to nearly 60%, whereas the effects of compounds **5**, **6**, **9**, and **10** did not change greatly. 

In addition, the impact on the malformation of zebrafish by these compounds was observed using a Leica stereomicroscope. Figure 4c showed graphically under the same treatment that compounds **5** and **8** had greater effects than other compounds on the mortality rate and malformation of zebrafish both at a concentration of 0.625 μM for 24 h and at a concentration of 10 μM for 72 h. In summary, our data demonstrated that compounds **6**, **9**, and **10** were of low toxicity and could be used against tea pathogenic fungi agents and deserve further optimization.

## 3. Discussion

During the analyses of natural products from the ethyl acetate (EtOAc) extract of anti-bacteria from special growing environment, we discovered five new sorbicillinoids (**1**–**5**) together with six known analogs (**6**–**11**). These compounds were obtained from the marine-derived *H. jecorina* (H8) from the mangrove sediments collected from Zhangjiangkou Mangrove National Nature Reserve, China. 

Chemically, the configurations and absolute of these compounds were described by their NOESY and CD spectra, respectively. All the isolated compounds of anti-tea pathogenic fungus *Pestalotiopsis theae* activities were evaluated. Compounds **5**, **6**, **8**, **9**, and **10** had stronger inhibitory effects against the fungi assays compared with hexaconazole.

However, the security of the antifungal regents is an important factor for use in agricultural applications. Although it showed potent activity, compound **5** exhibited strong anti-proliferative effects on the embryonic development of zebrafish, and compound **8** killed zebrafish embryos more than 50% at both a concentration of 0.625 μM for 24 h and a concentration of 10 μM for 72 h, thus, indicating high toxicity. Compounds **6**, **9**, and **10** showed much lower toxicity to zebrafish. In summary, sorbicillinoid derivatives (**6**, **9**, and **10**) from *H. jecorina* H8 had low toxicity and potent potency against tea pathogenic fungus.

## 4. Materials and Methods

### 4.1. General Experimental Procedures

An electrospray ionization source (ESI)-equipped Q-Exactive Mass spectrometer (Thermo Fisher Scientific Corporation, Waltham, MA, USA) was used to analyse the HR-ESI-MS data. A Shimadzu UV-260 spectrometer (Shimadzu Corporation, Tokyo, Japan) and a Perkin–Elmer 683 infrared spectrometer (PerkinElmer, Inc., Waltham, MA, USA) were used to obtain the UV and IR spectra, respectively. A JASCO P-200 polarimeter (JASCO Corporation, Tokyo, Japan) with a 5 cm cell was applied to measure the optical rotation value. The NMR spectra with TMS as the internal standard were taken on a Brucker Avance III 600 FT NMR spectrometer (Bruker Corporation, Billerica, MA, USA).

Column chromatography was performed with silica gel (Yantai Chemical Industry Research Institute, Shandong, China), Cosmosil 75 C18-MS-II (75 μm, Nacalai Tesqye corporation, Kyoto, Japan), and Spehadex LH-20 (GE Healthcare, Danderyd, Sweden). Semi-preparative HPLC was conducted on an Aglient HPLC (Agilent Technologies Inc., Santa Clara, CA, USA) system equipped with a diode array detector via a preparative Cosmosil ODS column. The HR-ESI-MS spectra were measured using a thermo Q-Exactive Mass spectrometer (Thermo Fisher Scientific Corporation). 

### 4.2. Eletronic Circular Dichroism (ECD) Calculations

The circular dichroism (CD) spectra were recorded on a MOS-500 dichroism spectrometer. The conformational analyses were conducted with MOE 2018 using MMFF94. All calculations were conducted with Gaussian 09 using various functionals (b3lyp/6-31+g(d) and cam-b3lyp/6-31+g(d)). The overall theoretical ECD data were weighted by Boltzmann distribution, and the ECD spectra were produced by SpecDis 1.70 software [6,16].

### 4.3. Fungus Carbohydrate Fermentation

*H. jecorina* (H8) was isolated from the mangrove sediments collected from Zhangjiangkou Mangrove National Nature Reserve, Fujian province, China. The strain was identified as *Hypocrea jecorina* on the basis of the internal transcribed spaces (ITS) sequence. The ITS region of the fungus was a 636 bp DNA sequence (GenBank accession number OL376355), which had 99% identity to *Hypocrea jecorina*. The fungal strain has been preserved at Third Institute of Oceanography, China. *P. theae* (ITS GenBank accession number HQ832793) was isolated from foliar lesions of the tea leaf, and its pathogenicity to tea leaves was verified both in vitro and in vivo.

### 4.4. Extraction and Isolation

The fungus H8 was cultivated on rice-artificial sea water medium, incubated at 28 °C for 20 days in a standing position. After 20 days of fermentation, the solid cultures were dispersed in water (H_2_O) and extracted with EtOAc (1:1, *v/v*) three times. The EtOAc extract was concentrated under reduced pressure at 40 °C to afford 22.0 g residue. The residue (21 g) was subjected to silica gel CC with petroleum ether (PE)-EtOAc (*v/v*) (20:1; 10:1; 5:1; 2:1; 1:1; 0:1) and chloroform (CDCl_3_)-methanol (MeOH) (*v/v*) (100:1; 50:1; 20:1; 10:1; 5:1; 100% MeOH) to yield nine fractions (Fr. 1–9). 

Fr. 2 (400 mg) was subjected to octadecylsilyl (ODS) chromatography and eluted with MeOH-H_2_O (80–100%) to give 10 subfractions (Fr. 2A–2J). Then, Fr. 2G was purified using preparative reversed-phase HPLC C18 column (pre. Rp-C18) and isocratic eluted with MeOH-H_2_O (85%) to obtain compound **6** (50 mg). Fr.3 (500 mg) was subjected to CC over ODS gel eluting with to MeOH-H_2_O (70–100%) to obtain four subfractions (Fr. 3A-Fr.3D). Subfraction Fr.3C was purified by Prep. Rp-C18 using MeOH-H_2_O (74%) elution to obtain **7** (11.9 mg). Fraction Fr.4 (496 mg) was subjected to CC over Sephadex LH-20 (MeOH) to yield six subfractions (Fr.4A–Fr.4F). 

Compounds **1** (1.3 mg), **3** (1.1 mg), **10** (4.4 mg), and **8** (10 mg) were separated from subfraction Fr.4C by Prep. Rp-C18 (MeOH-H_2_O, 80%). Fraction Fr.5 was purified by CC on Sephadex LH-20 (MeOH) to yield four subfractions (Fr.5A–Fr.5D), and then fraction Fr.5B was purified by Prep. Rp-C18 isocratic eluted with MeOH-H_2_O (80:20) to provide **2** (5.5 mg). Fr. 5C was also purified by Prep. Rp-C18 isocratic eluted with MeOH-H_2_O (52:48) to obtain compound **4** (1.0 mg). Compounds **5** (9.3 mg), **9** (20 mg), and **11** (25 mg) were separated from subfraction Fr.5D by Prep. Rp-C18 (MeOH-H_2_O, 80%).

### 4.5. Structrural Elucidation of the New Compounds **1**–**5**

Trichodermolide C (**1**): yellow amorphous powder; HR-ESI-MS *m/z* 375.1798 [M + H]^+^ (calcd. for 375.1729 C_21_H_27_O_6_^+^) and 397.1609 [M + Na]^+^ (calcd. for 397.1627 C_21_H_26_O_6_Na^+^) in the positive mode; [α]${}_{D}^{30}$ = +54° (*c* = 0.5, MeOH). IR (KBr) (*ν*max): 3385, 1646, and 1436 cm^−1^. ^1^H NMR and ^13^C NMR data are listed in Table 1.

Trichodermolide D (**2**): yellow amorphous powder; HR-ESI-MS *m/z* 363.2162 [M + H]^+^ (calcd. for 363.2093 C_21_H_31_O_5_^+^) and 389.1972 [M + Na]^+^ (calcd. for 385.1991 C_21_H_30_O_5_Na^+^) in the positive mode; [α]${}_{D}^{30}$ = +105° (*c* = 0.5, MeOH). IR (KBr) (*ν*max): 3406, 2927, 1560, and 1430 cm^−1^. ^1^H NMR and ^13^C NMR data are listed in Table 1.

7, 7′, 9′-Hydroxy-trichodimerol (**3**) isolated as a yellow amorphous powder; HR-ESI-MS *m/z* 51.2265 (calcd. for 515.2281 C_28_H_35_O_9_^+^) and 537.2090 [M + Na]^+^ (calcd. for 537.2100 C_28_H_34_O_9_Na^+^) in the positive mode; [α]${}_{D}^{30}$ = −35° (*c* = 0.5, MeOH), IR (KBr) *ν*max): 3406, 1570, and 1430 cm^−1^. UV (MeOH) *λ*max (log *ε*): 286 (4.39) and 359 (4.46) nm. ^1^H NMR and ^13^C NMR data are listed in Table 2.

1-(2,4-Dihydroxy-3,5-dimethylphenyl)-3,4,5-trihydroxyhexan-1-one (**4**): colorless amorphous powder; HR-ESI-MS *m/z* 285.1342 [M + H]^+^ (calcd. for 285.1338 C_14_H_21_O_6_^+^) in the positive mode; [α]${}_{D}^{30}$ = +6° (*c* = 0.05, MeOH), IR (KBr) (*ν*max): 3416, 2927, 1601, 1367, 1188, and 1075 cm^−1^. UV (MeOH) *λ*max (log *ε*): 216 (3.66), 285 (3.72), and 329 (3.41) nm. ^1^H NMR and ^13^C NMR data are listed in Table 1.

Isobisvertinol A (**5**): white amorphous powder; HR-ESI-MS *m/z* 499.2312 [M + H]^+^ (calcd. for 499.2326 C_28_H_35_O_8_^+^) in the positive mode, and 497.2194 [M − H]^-^ (calcd. for 497.2181 C_28_H_33_O_8_^−^) in the negative mode; [α]${}_{D}^{30}$ = −46.2° (*c* = 0.5, MeOH), IR (KBr) (*ν*max): 3416, 1570, and 1430 cm^−1^. UV (MeOH) *λ*max (log *ε*): 278 (3.44) and 374 (3.52) nm. ^1^H NMR and ^13^C NMR data are listed in Table 2.

### 4.6. Antifungal Activity Assay

Initial evaluations of the antifungal activity of the purified compounds were conducted against tea pathogenic fungus *P. theae* in six-well microplates as described by Xia Yan with certain modifications [17]. The final concentrations of each compound in the wells were 80, 40, 20, 10, 5, and 2.5 µg/mL (two-fold dilutions). DMSO and hexaconazole were used as a blank control and positive control, respectively. The ED_50_ (μg/mL) values were calculated statistically using Probit analyses.

### 4.7. Toxicity Evaluation

Wild-type (AB strain) zebra fish (Danio rerio) were used in this test. Compounds **5**, **6**, **8**, **9**, and **10** were dissolved in DMSO (10 mM) and stored at −20 °C. Toxicity evaluations were analyzed regarding the anti-proliferative effects on embryos according to the literature [18]. In brief, 15 zebrafish embryos per condition were exposed to compounds at the concentrations of 10, 5, 2.5, 1.25, and 0.625 μM, and 0.1% DMSO was used as the vehicle control. A Leica stereomicroscope was used to observe the embryos every 24 h.

## Figures and Tables

**Table 1:** ^1^H NMR (600 Hz) and ^13^C NMR (150 Hz) data of **1**–**2** (CDCl_3_) and **4** (DMSO-*d*_6_).

NO.	1		Trichodermolide B	2		4	
	*δ*_H_ (*J* in Hz)	*δ*_C_	*δ*_C_	*δ*_H_ (*J* in Hz)	*δ*_C_	*δ*_H_ (*J* in Hz)	*δ*_C_
2		174.9	174.7		176.4		113.6
3		55.7	55.9		56.2		159.9
4		149.2	150.9		152.1		111.7
5		134.4	133.9		133.8		160.3
6		191.3	191.2		191.9	7.34	118.4
7		86.8	86.5		87.1		125.1
8	1.77 s	11.6	11.9	1.87 s	12.1	2.89 dd (16.9, 13.7) 2.31 dd (16.9, 2.7)	192.1
9	1.48 s	16.4	16.5	1.56 s	17.3	4.64 dt (13.7, 2.6)	39.8
10	1.30 dq (13.7, 7.2) 2.08 dq (13.8, 7.2)	20.5	20.5	2.21 dq (14.2, 7.3) 1.75 s	20.9	3.09 td (8.5, 1.5)	77.1
11	0.95 t (7.2)	8.4	8.6	0.97 t (7.2)	8.6	3.88 dq (13.6, 6.0)	76.8
12	3.55 d (18.2) 3.63 d (18.2)	44.5	44.7	2.60 dd (13.7, 9.6) 2.39 brd (13.6)	39.2	1.19 d (6.0)	65.9
13		204	205.0	4.10 m	67.1	2.04 s	21.5
14	2.27 s	30.3	30.5	1.29 t (7.2)	24.1	2.12 s	9.2
15	3.55 t (5.3)	49.9	50.0	2.97 dd (6.4, 4.4)	51.3		16.7
16	2.43 dd (18.5, 5.2) 3.13 dd (18.5, 5.2)	35.2	35.0	1.52 m 1.37 m	32.3		
17		196.5	197.2	4.17 m	71.1		
18	6.43 br dd (15.4, 10.2)	127.9	127.6	6.16 dd (15.2, 10.5)	132.1		
19	7.25 dd (15.2, 10.8)	143.0	143.9	5.44 dd (15.2, 7.2)	131.9		
20	6.20 d (15.4)	128.8	130.6	5.75 dq (14.3, 6.8)	131.7		
21	6.35 dt (15.2, 4.8)	143.2	141.9	5.99 dd (15.2, 10.7)	130.0		
22	4.32 d (4.2)	62.7	19.1	1.76 br d (6.6)	18.2		

**Table 2:** ^1^H NMR (600 Hz) and ^13^C NMR (150 Hz) data of **3**, **5** (CDCl_3_) and ^13^C NMR data of Compounds **10** and **11**.

NO.	3		10	NO.	5		11
	*δ*_H_ (*J* in Hz)	*δ*_C_	*δ*_C_		*δ*_H_ (*J* in Hz)	*δ*_C_	*δ*_C_
1	2.94, s	58.3	57.5	1		191.8	194.3
1′	3.31, s	52.3	57.5	2		100.7	101.9
2,2′		78.3	78.9	3	2.58 br d (14.1) 2.73 m	35.9	35.4
3,3′		108.7	104.1	4		73.9	72.7
4		59.0	58.8	5		168.7	168.8
4′		59.0	58.8	6		103.9	105.6
5,5′		199.0	198.0	7		191.7	191.6
6′		104.0	102.8	8		106.1	106.2
6		103.8	102.8	1′		179.7	178.0
7		172.5	175.9	2′	6.40 br d (14.9)	120.3	121.4
7′		175.2	175.9	3′	7.25 br s	139.0	137.8
8	6.18 d (14.7)	118.6	118.5	4′	6.21 m	130.8	131.4
8′	2.38 dd (16.9, 3.5) 2.54 dd (16.9,13.6)	40.9	118.5	5′	6.10 br d (7.0)	131.1	131.7
9	7.35 dd (14.8,10.9)	143.0	143.6	6′	1.86 br d (6.4)	18.8	18.9
9′	4.31 ddd (13.5, 7.1,3.2)	80.7	143.6	1″		168.8	167.3
10	6.31 dd (15.4, 11.0)	130.8	130.9	2″	6.27 m	120.2	121.3
10′	5.67 dq (15.2,6.6)	131.8	130.9	3″	6.29 m	140.3	140.1
11	6.22 dd (13.6,6.6)	140.1	140.5	4″	6.23 br s	131.8	131.4
11′	5.49 ddd (15.3,7.0,1.6)	127.2	140.5	5″	6.18 m	137.0	136.6
12	1.91 brd (6.6)	18.9	18.8	6″	1.86 br d (6.4)	18.9	19.1
12′	1.68 brd (6.4)	17.8	18.8	1a		58.4	59.1
13	1.43 s	21.5	21.3	1a-CH_3_	1.34 s	19.2	19.9
13′	1.35 s	20.7	21.3	4a		110.2	108.4
14	1.41 s	18.2	18.9	4-CH_3_	1.29, s	22.4	22.8
14′	1.41 s	18.4	18.9	5a		79.6	78.8
				5a-CH_3_	1.48 br s	25.6	25.8
				6-CH_3_	1.53, s 1.86 br d (6.4)	6.8	7.3
				8a	3.63, s	53.6	53.6

**Table 3:** Antifungal activities of **5**, **6**, **8**, **9**, and **10** (ED_50_, µg/mL).

Compd.	*Pestalotiopsis theae*	Compd.	*Pestalotiopsis theae*
1	>100	8	18.22 ± 1.29
2	>100	9	1.83 ± 1.37
3	>100	10	4.68 ± 1.44
4	>100	11	>100
5	9.13 ± 1.25	hexaconazole *	24.25 ± 1.57
6	2.04 ± 1.91	DMSO	None
7	>100		

* hexaconazole serves as positive control.

## Supplementary Materials

The following are available online at https://www.mdpi.com/article/10.3390/md20030213/s1, Figures S1–S8: Spectra of **1**, Figures S9–S16: Sepctra of **2**, Figures S17–S25: Spectra of **3**, Figures S26–S32: Spectra of **4**, Spectra of **3**, Figures S33–S40: Spectra of **5**, Figure S41: ECD spectra of trichodermolide B, Figure S42: HR-ESI-MS spectra of 11. ECD calculation of **1**–**3**.
