# Supplementary material for: New Sorbicillinoids with Tea Pathogenic Fungus Inhibitory Effect from Marine-Derived Fungus Hypocrea jecorina H8
Source: Mar Drugs. 2022 Mar 17;20(3):213. doi: 10.3390/md20030213 (PMC8955853; doi:10.3390/md20030213)
Supplement: Supplementary file 1 [file marinedrugs-20-00213-s001.zip › marinedrugs-1564270-supplementary.pdf]

Figure S1. HR-ESI-MS spectra of compound **1**......2
Figure S2 ^{1}H-NMR (600 MHz, $CDCl_3$) of compound **1**......2
Figure S3 ^{13}C-NMR (150 MHz, $CDCl_3$) of compound **1**......3
Figure S4 DEPT of compound **1**......3
Figure S5. HSQC spectrum of compound **1**......4
Figure S6. COSY spectrum of compound **1**......4
Figure S7. HMBC spectrum of compound **1**......5
Figure S8. NOESY spectrum of compound **1**......5
Figure S9. HR-ESI-MS spectra of compound **2**......6
Figure S10 ^{1}H-NMR (600 MHz, $CDCl_3$) of compound **2**......6
Figure S11 ^{13}C-NMR (150 MHz, $CDCl_3$) of compound **2**......7
Figure S12 DEPT of compound **2**......7
Figure S13 HSQC spectrum of compound **2**......8
Figure S14 COSY spectrum of compound **2**......8
Figure S15 HMBC spectrum of compound **2**......9
Figure S16 NOESY spectrum of compound **2**......9
Figure S17 HR-ESI-MS spectra of compound **3**......10
Figure S18 UV spectrum of compound **3**......10
Figure S19 ^{1}H-NMR (600 MHz, $CDCl_3$) of compound **3**......11
Figure S20 ^{1}C-NMR (150 MHz, $CDCl_3$) of compound **3**......11
Figure S21 DEPT of compound **3**......12
Figure S22 HSQC spectrum of compound **3**......12
Figure S23 COSY spectrum of compound **3**......13
Figure S24 HMBC spectrum of compound **3**......13
Figure S25 NOESY spectrum of compound **3**......14
Figure S26 HR-ESI-MS spectra of compound **4**......15
Figure S27 UV spectrum of compound **4**......15
Figure S28 ^{1}H-NMR (150 MHz, DMSO-d_6) of compound **4**......16
Figure S29 ^{13}C-NMR (600 MHz, DMSO-d_6) of compound **4**......16
Figure S30 HSQC spectrum of compound **4**......17
Figure S31 COSY spectrum of compound **4**......17
Figure S32 HMBC spectrum of compound **4**......18
Figure S33 HR-ESI-MS spectra of compound **5**......19
Figure S34 UV spectrum of compound **5**......19
Figure S35 ^{1}H-NMR (600 MHz, $CDCl_3$) of compound **5**......20
Figure S36 ^{13}C-NMR (150 MHz, $CDCl_3$) of compound **5**......20
Figure S37. HSQC spectrum of compound **5**......21
Figure S38. COSY spectrum of compound **5**......21
Figure S39 HMBC spectrum of compound **5**......22
Figure S40 NOESY spectrum of compound **5**......22
ECD calculation of compound **1**......23
ECD calculation of compound **2**......29
ECD calculation of compound **3**......36
Figure S41 Calculated and experimental ECD spectra of trichodermolide B......59

Figure S42 HR-ESI-MS spectra of compound **11**..59

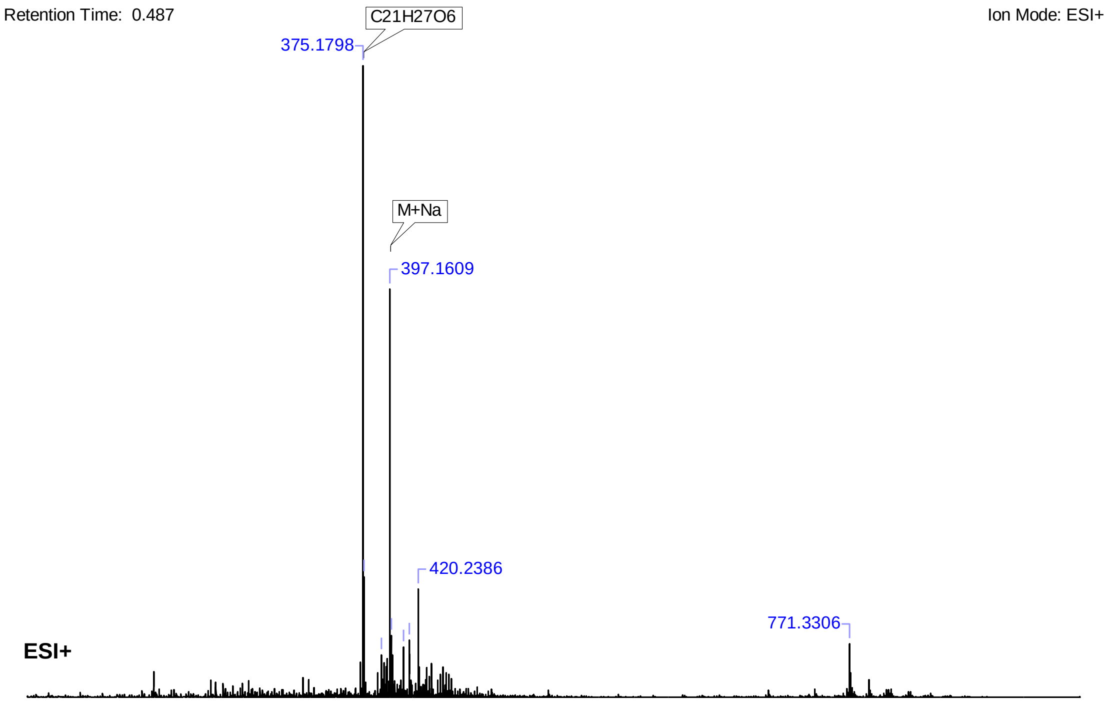

Figure S1. HR-ESI-MS spectra of compound **1**

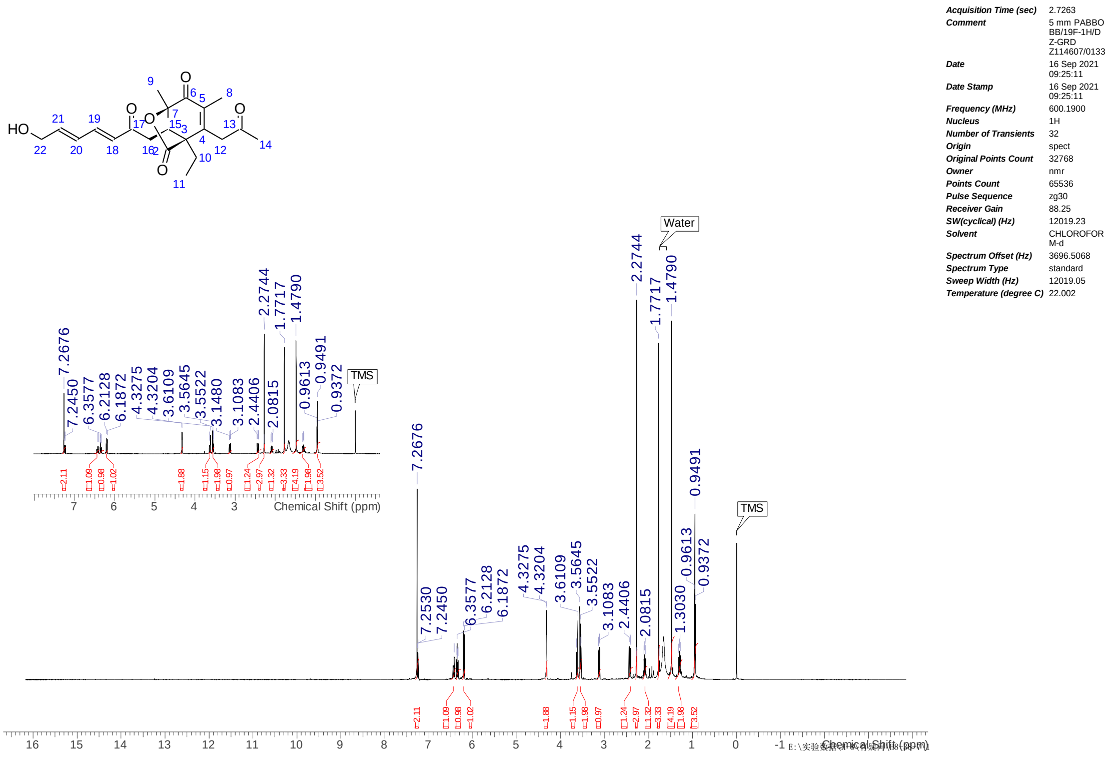

Figure S2 ^{1}H-NMR (600 MHz, $CDCl_3$) of compound **1**

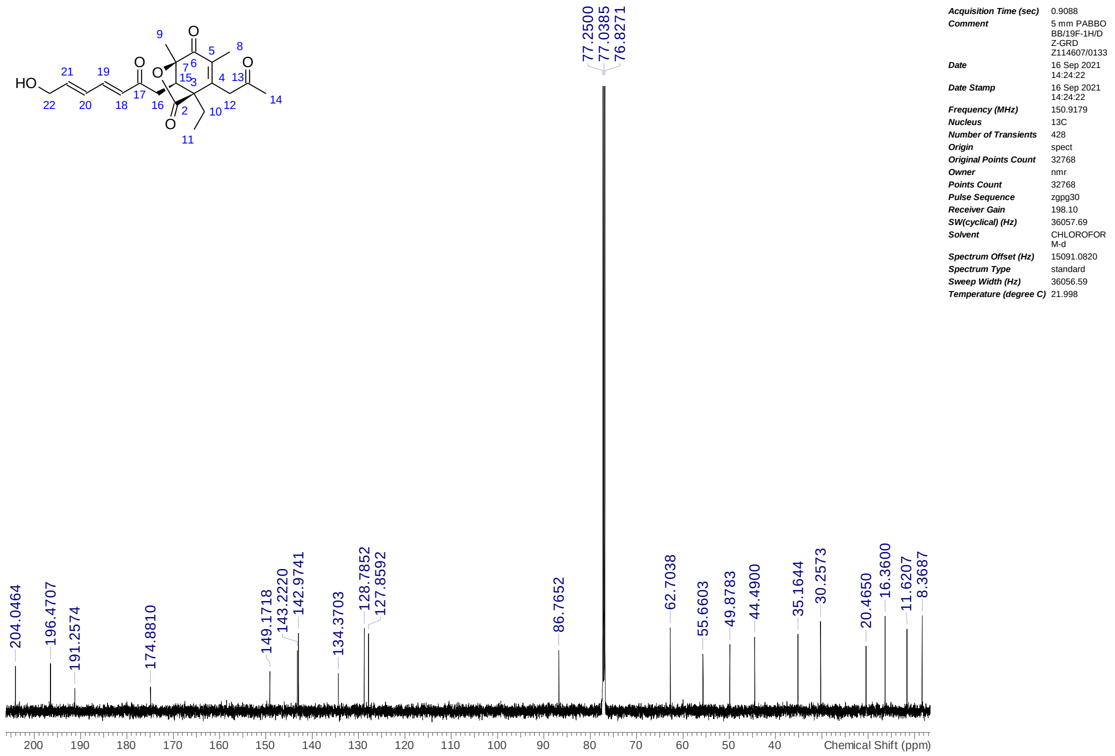

Figure S3 ^{13}C-NMR (150 MHz, $CDCl_3$) of compound **1**

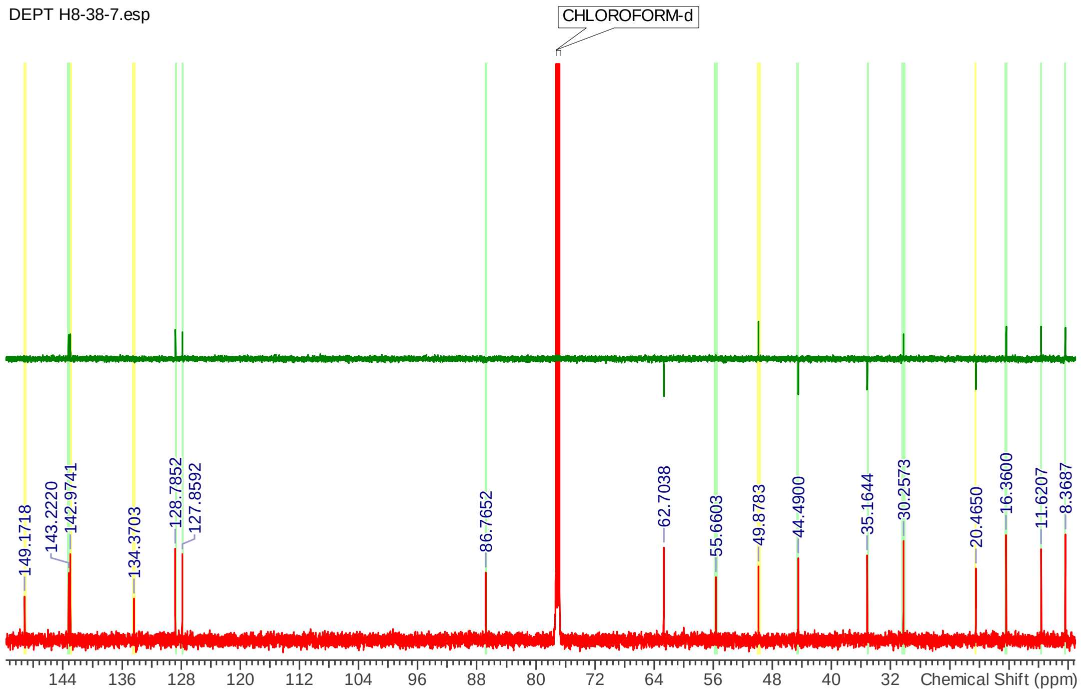

Figure S4 DEPT of compound **1**

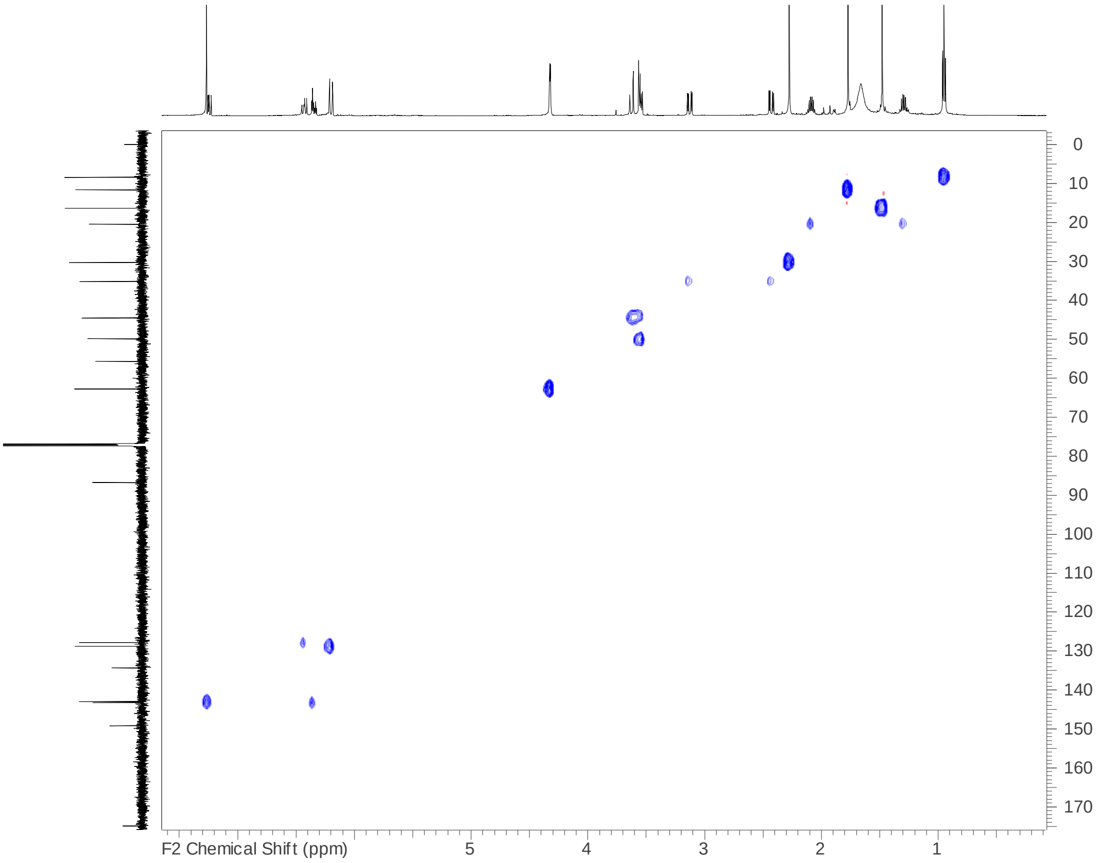

Figure S5. HSQC spectrum of compound **1**

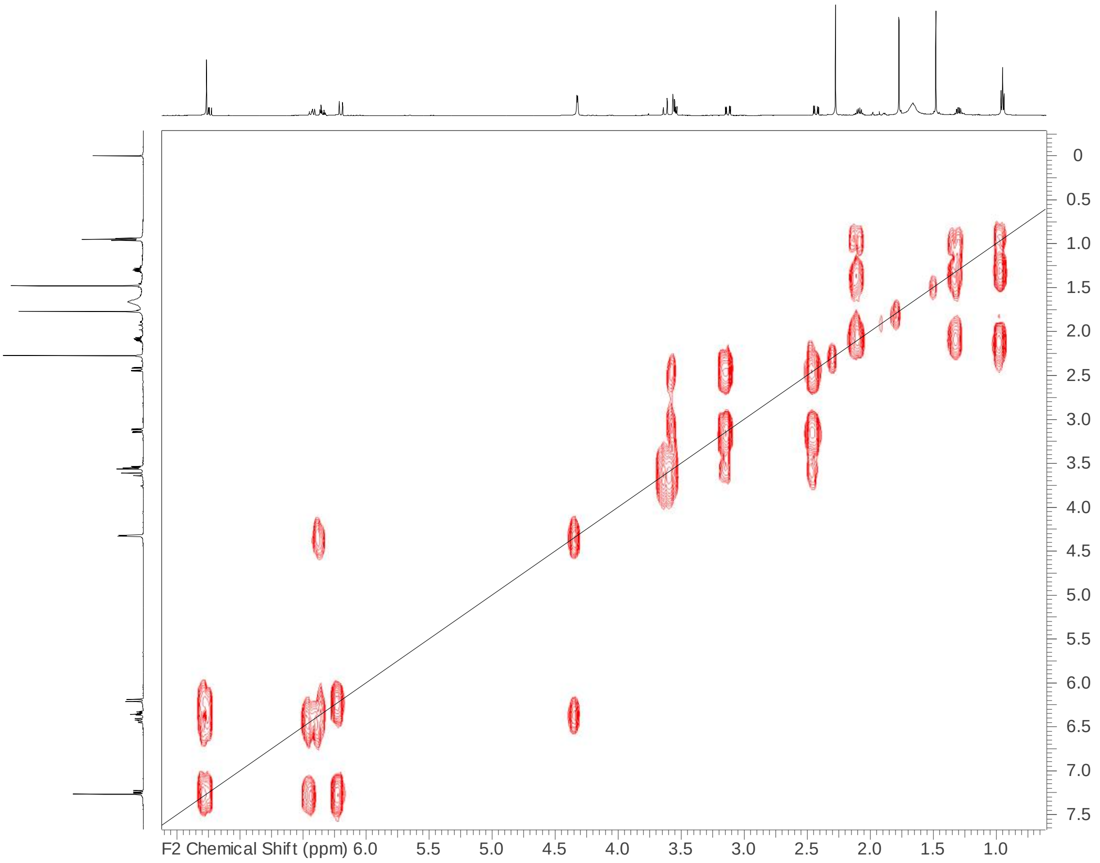

Figure S6. COSY spectrum of compound **1**

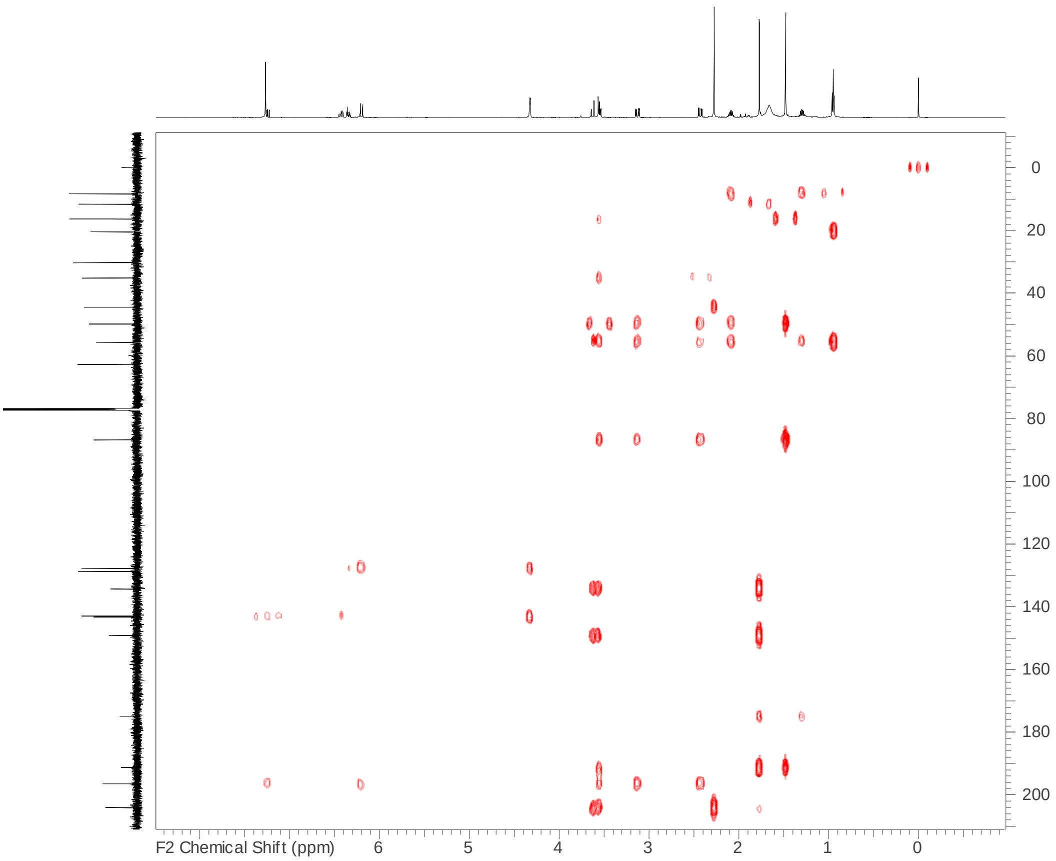

Figure S7. HMBC spectrum of compound **1**

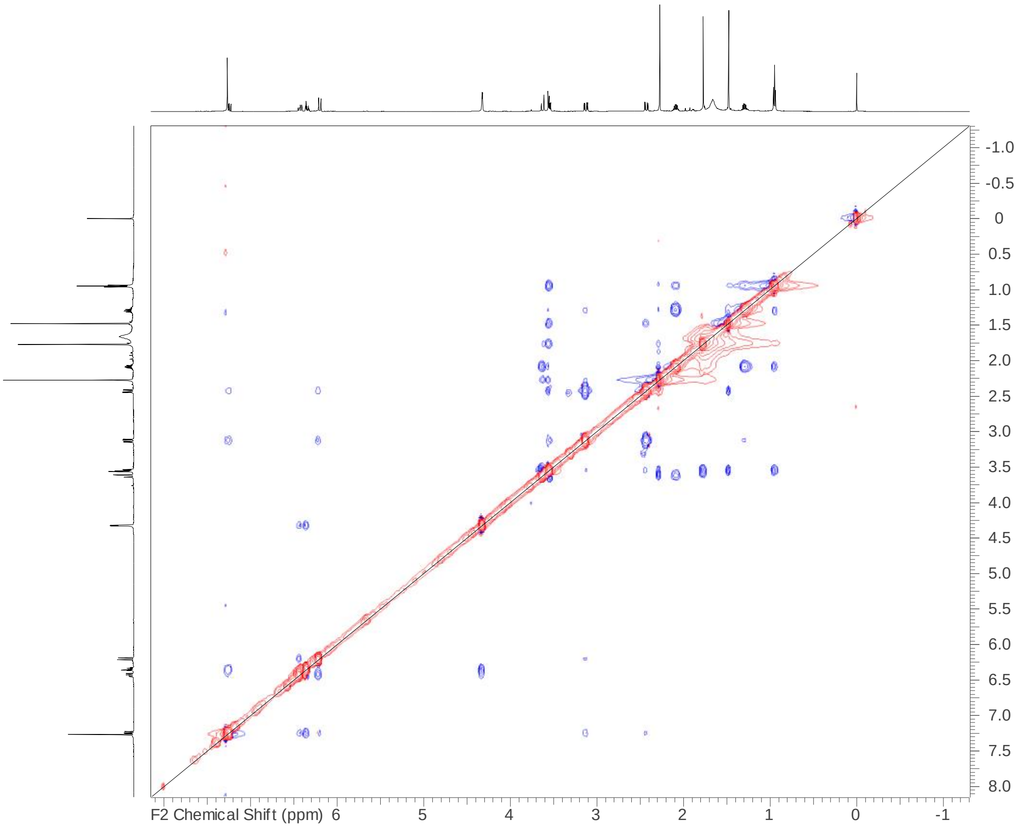

Figure S8. NOESY spectrum of compound **1**

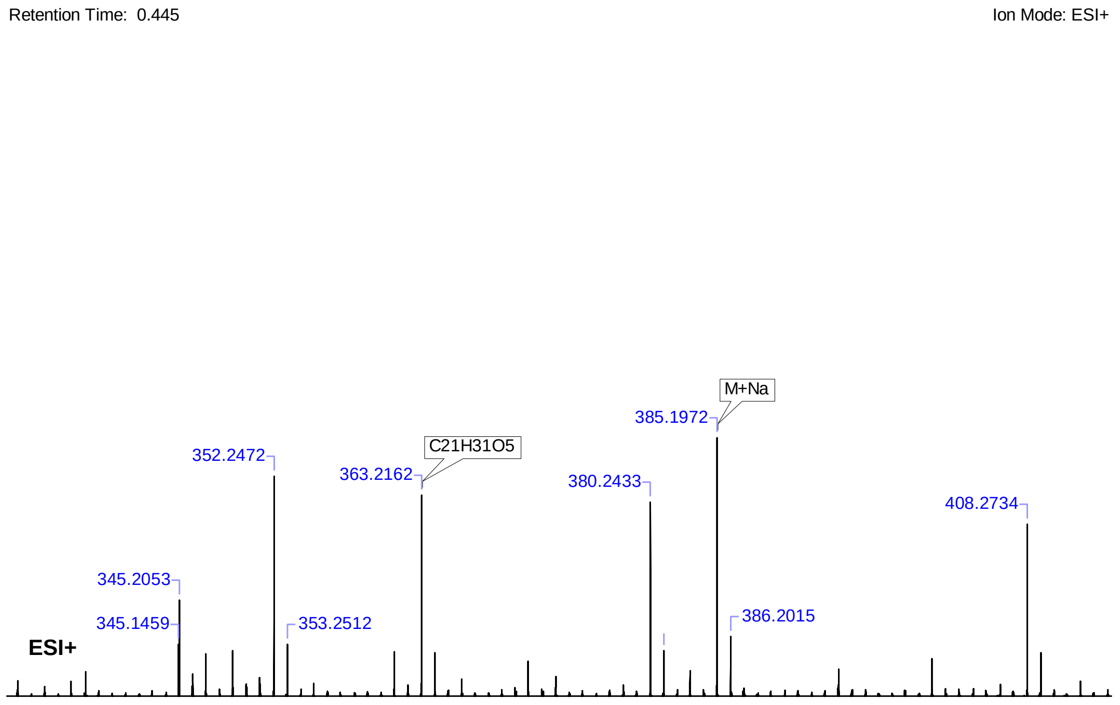

Figure S9. HR-ESI-MS spectra of compound **2**

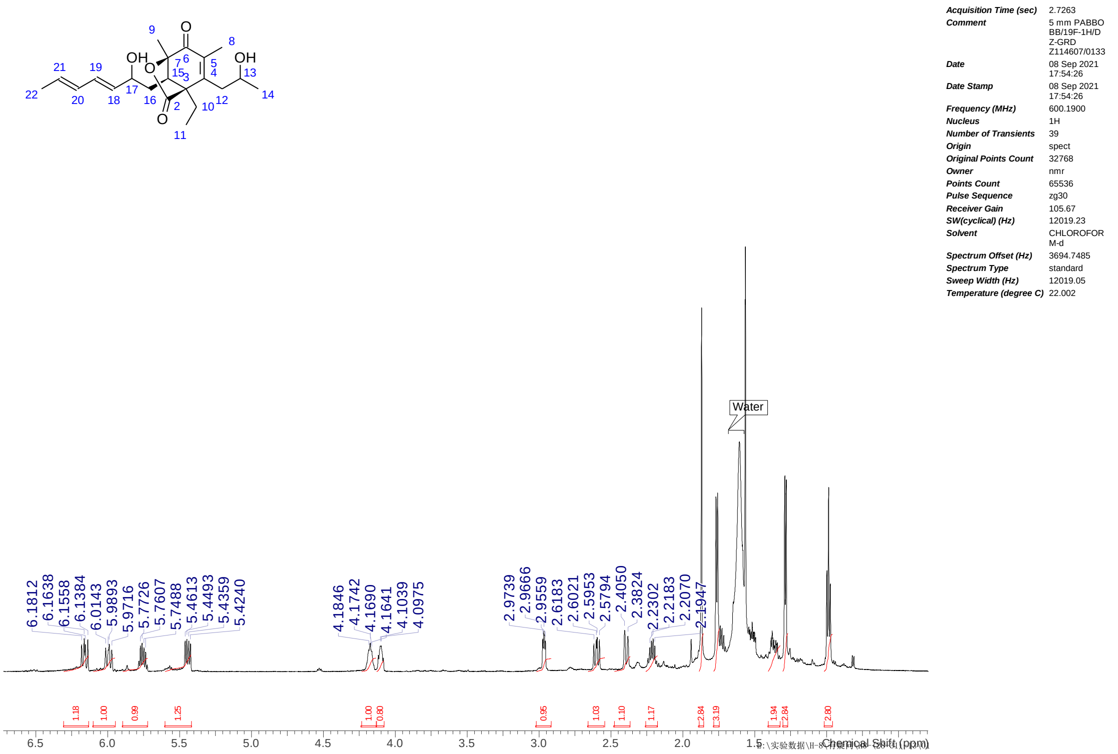

Figure S10 ^{1}H-NMR (600 MHz, $CDCl_3$) of compound **2**

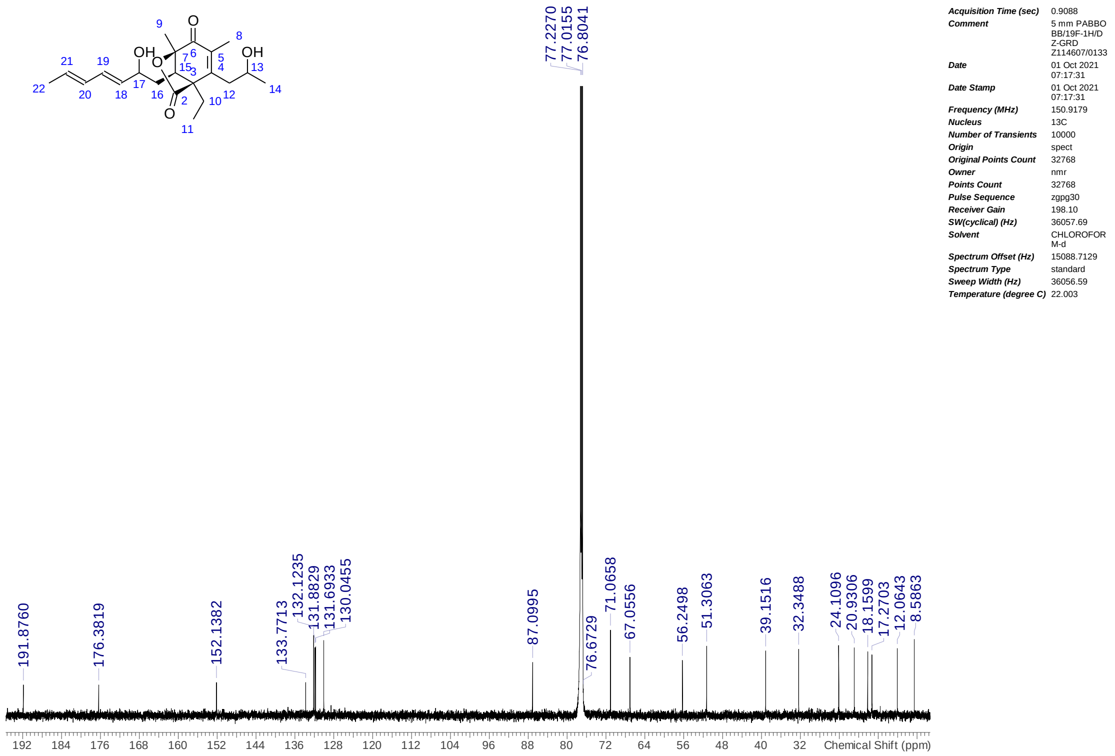

Figure S11 ^{13}C-NMR (150 MHz, $CDCl_3$) of compound **2**

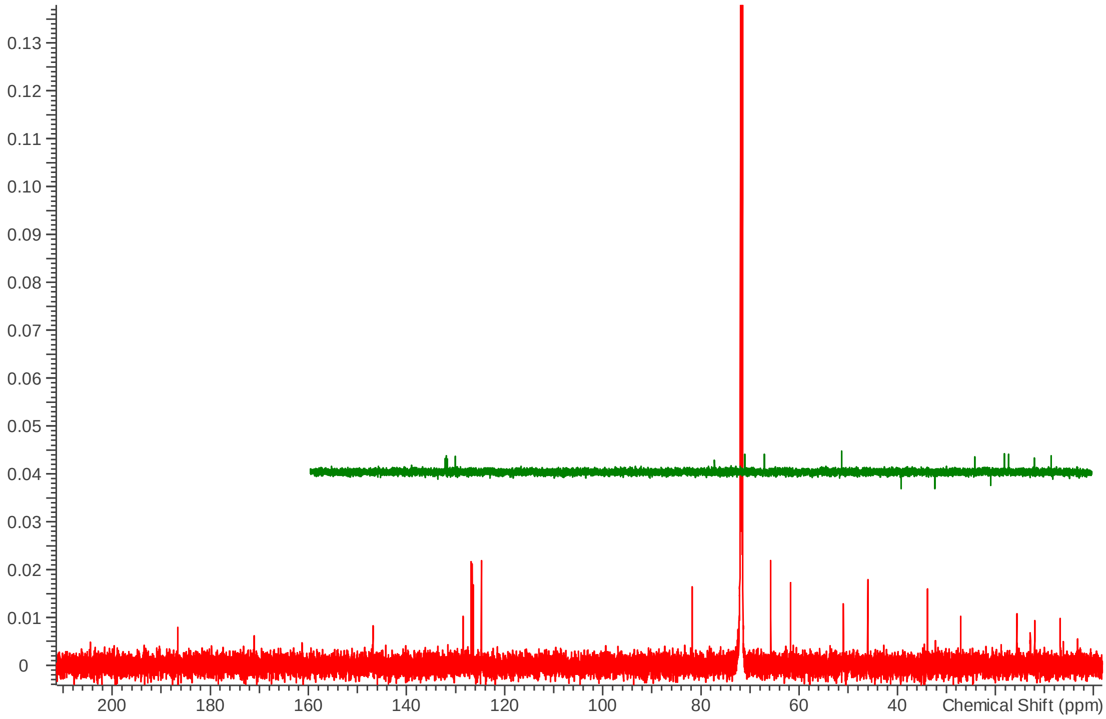

Figure S12 DEPT of compound **2**

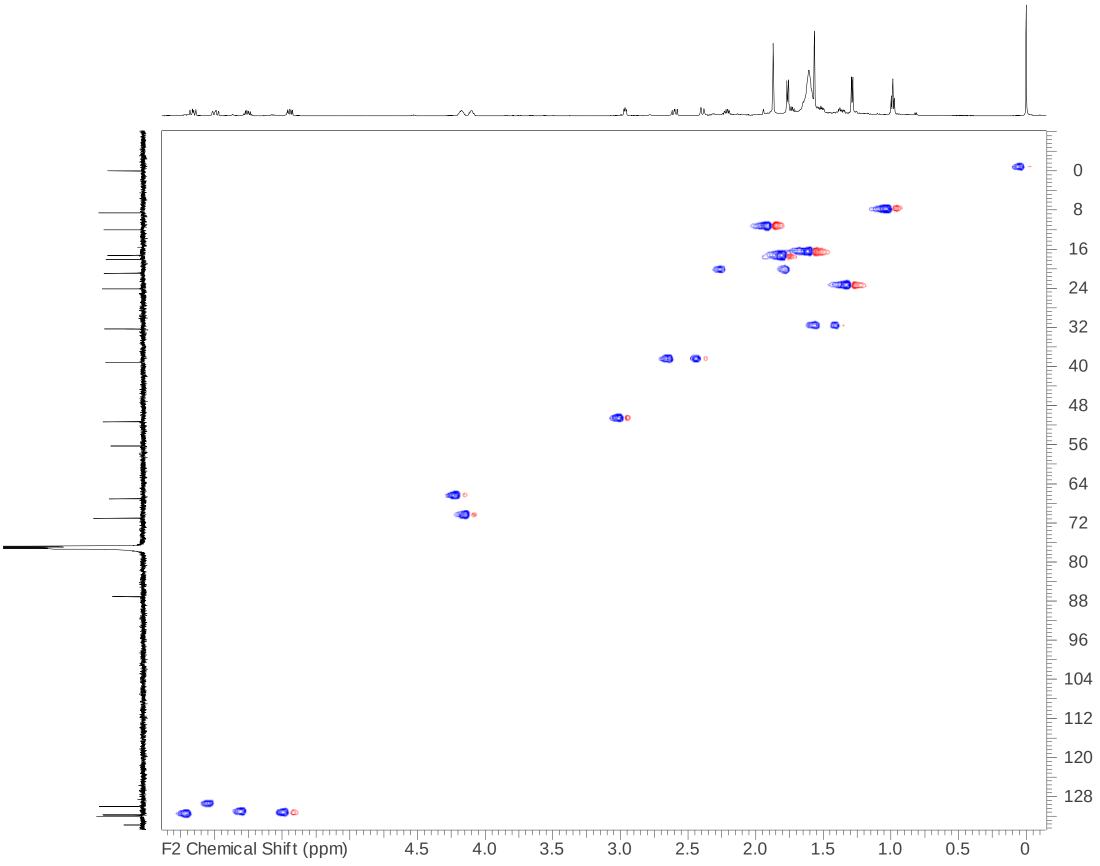

Figure S13 HSQC spectrum of compound **2**

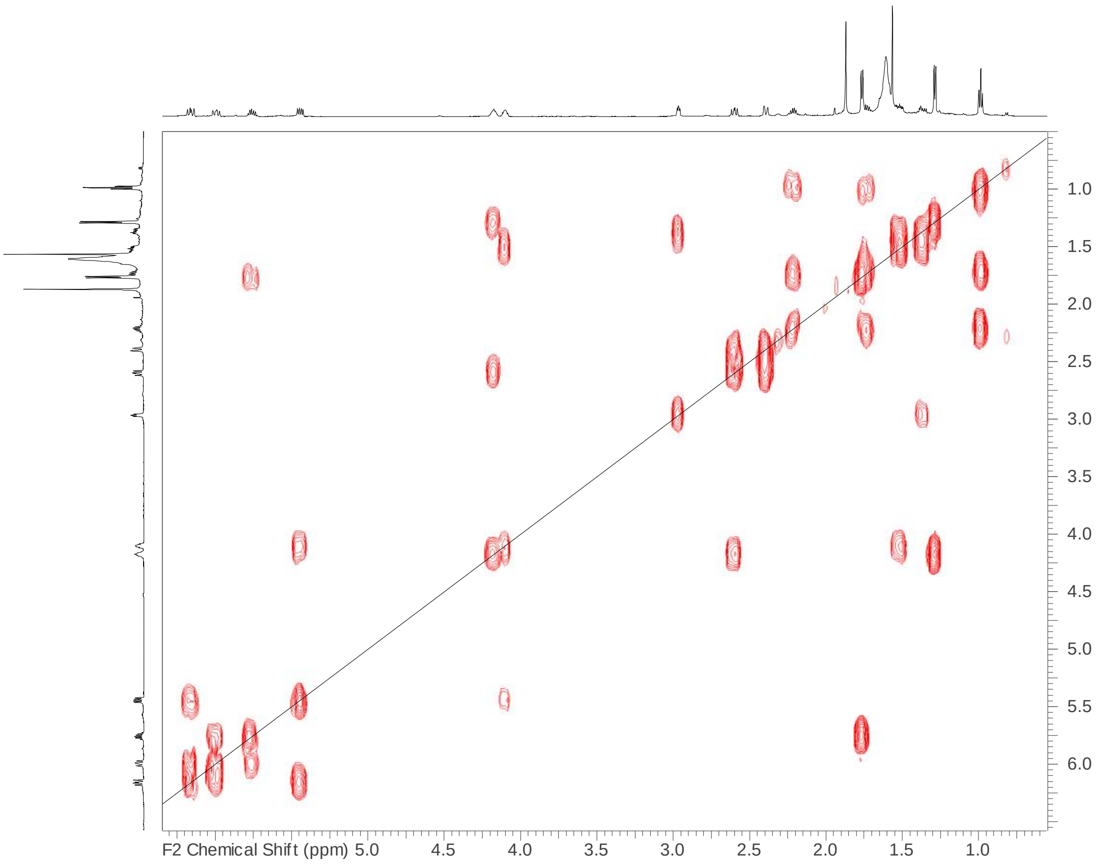

Figure S14 COSY spectrum of compound **2**

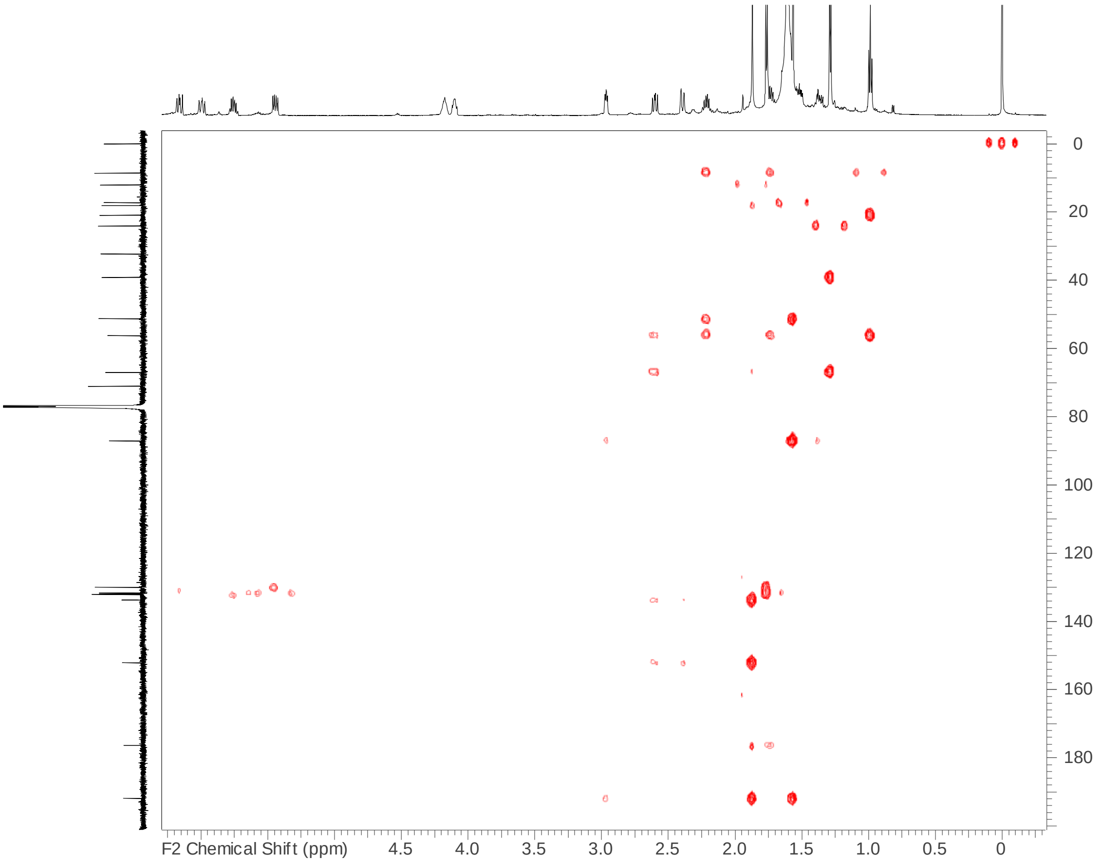

Figure S15 HMBC spectrum of compound **2**

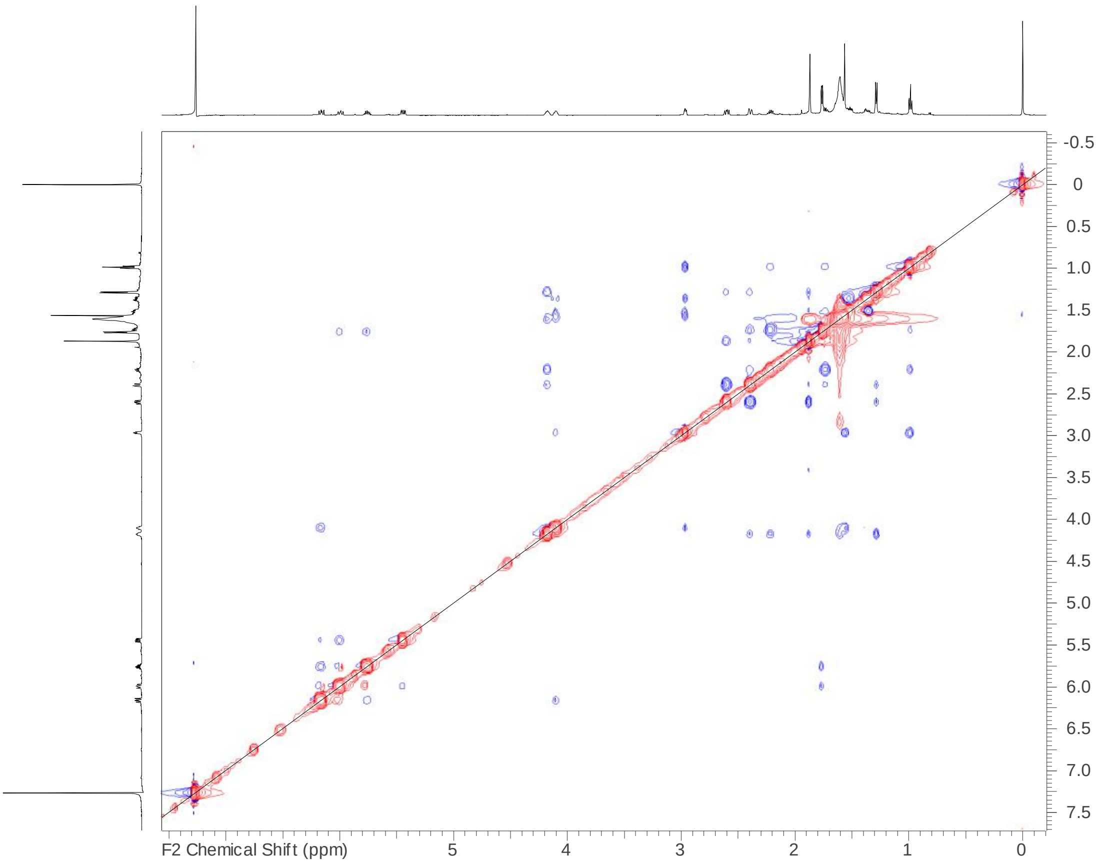

Figure S16 NOESY spectrum of compound **2**

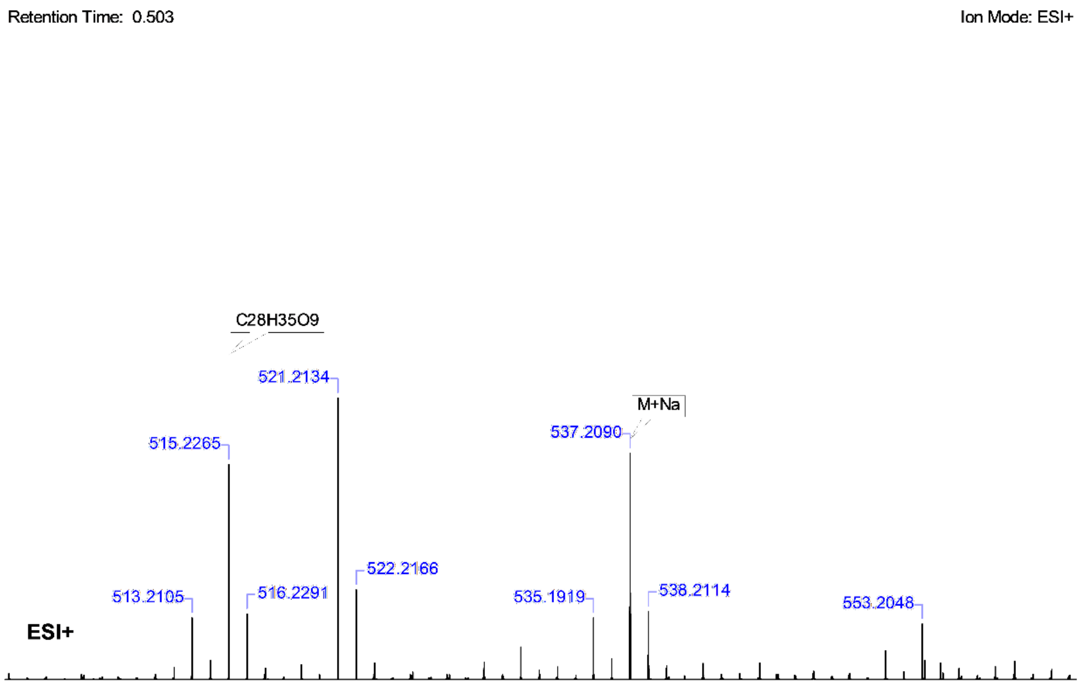

Figure S17 HR-ESI-MS spectra of compound **3**

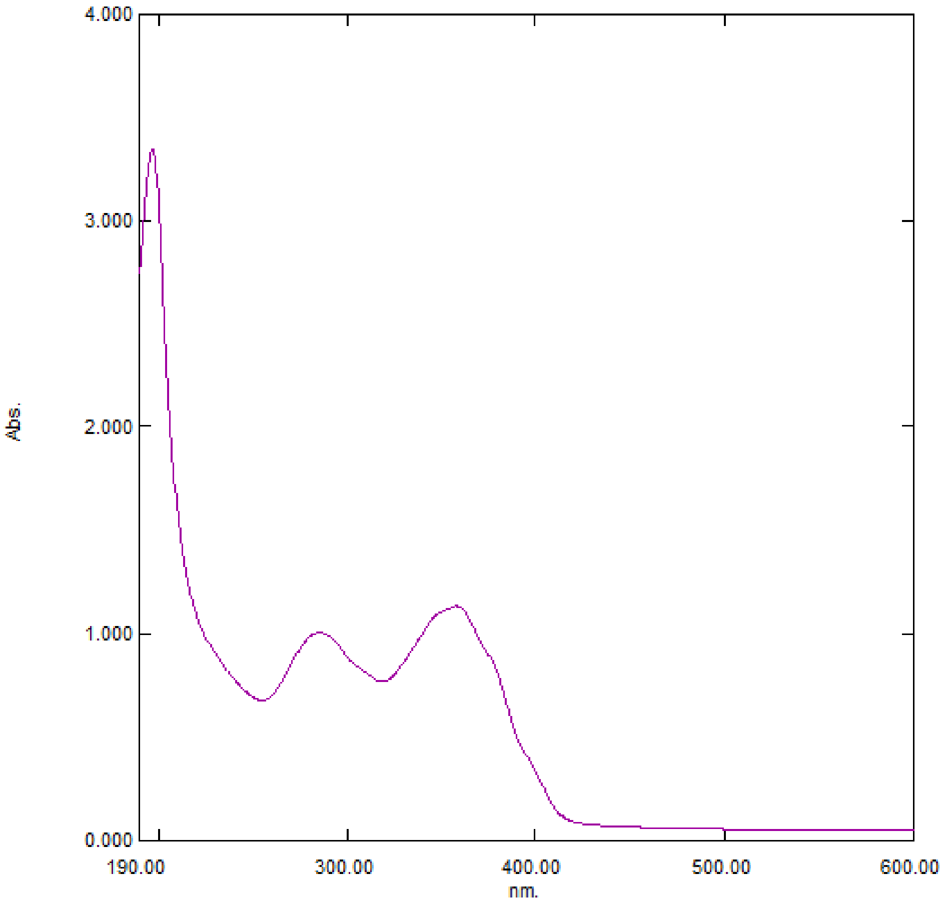

Figure S18 UV spectrum of compound **3**

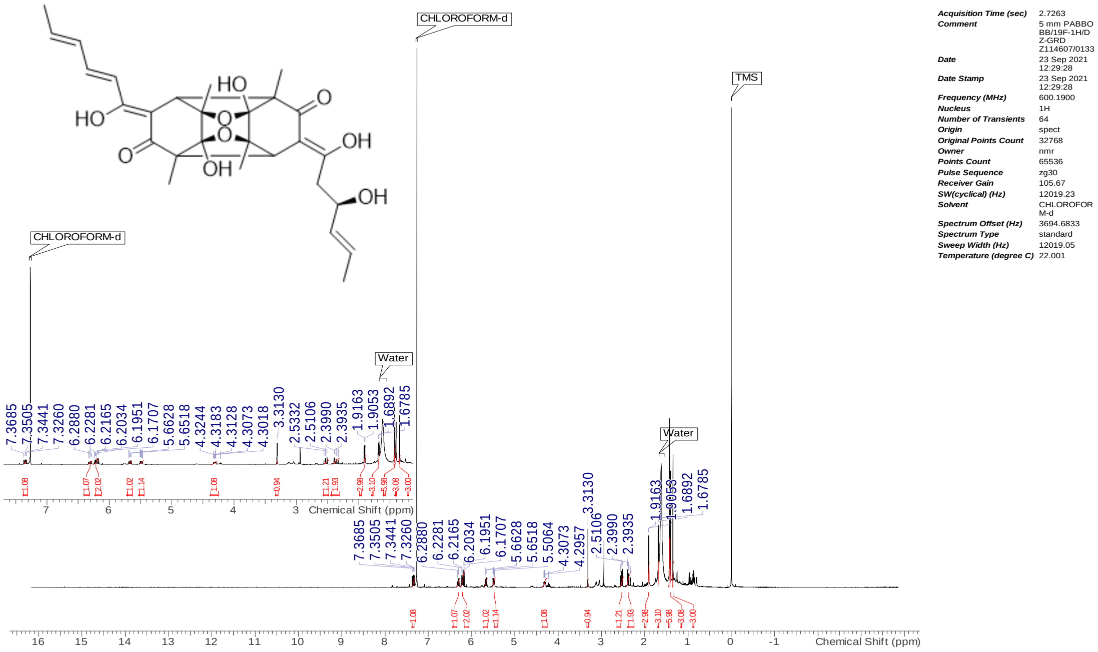

Figure S19 ^{1}H-NMR (600 MHz, $CDCl_3$) of compound **3**

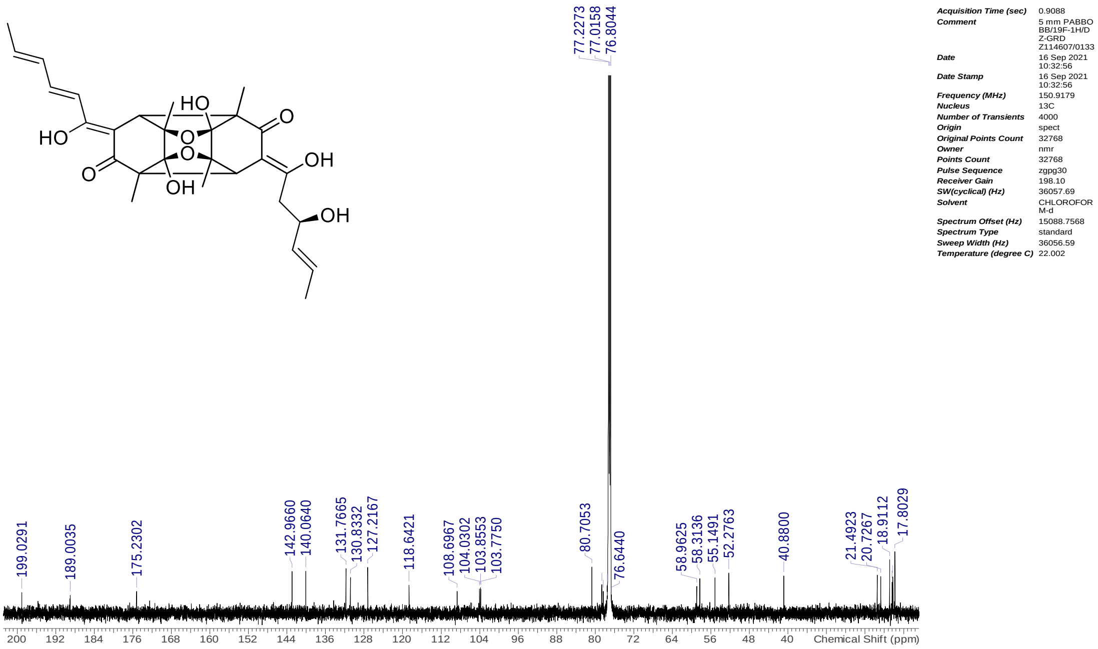

Figure S20 [1]C-NMR (150 MHz, $CDCl_3$) of compound **3**

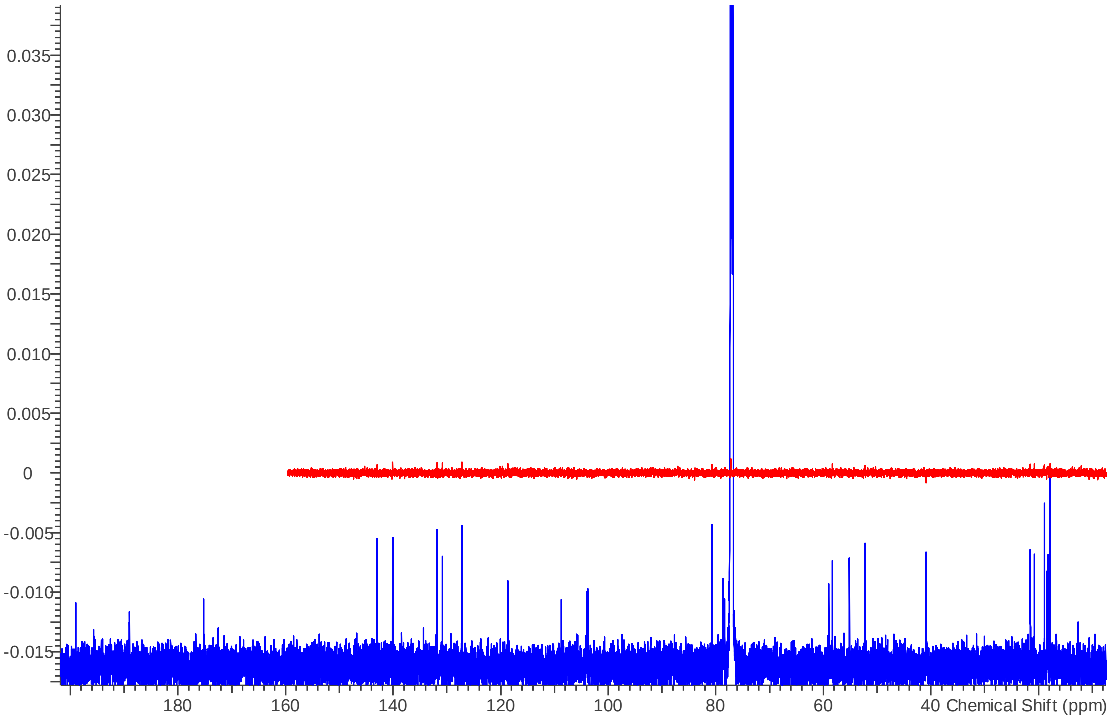

Figure S21 DEPT of compound **3**

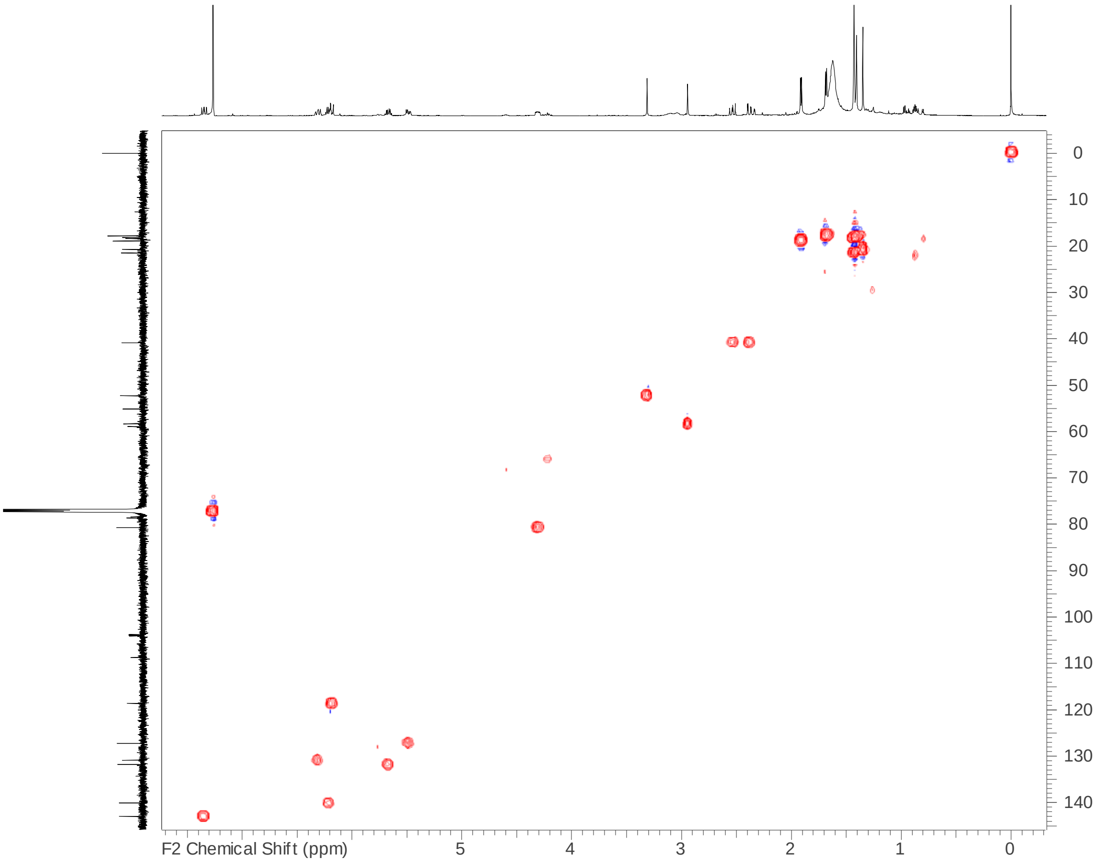

Figure S22 HSQC spectrum of compound **3**

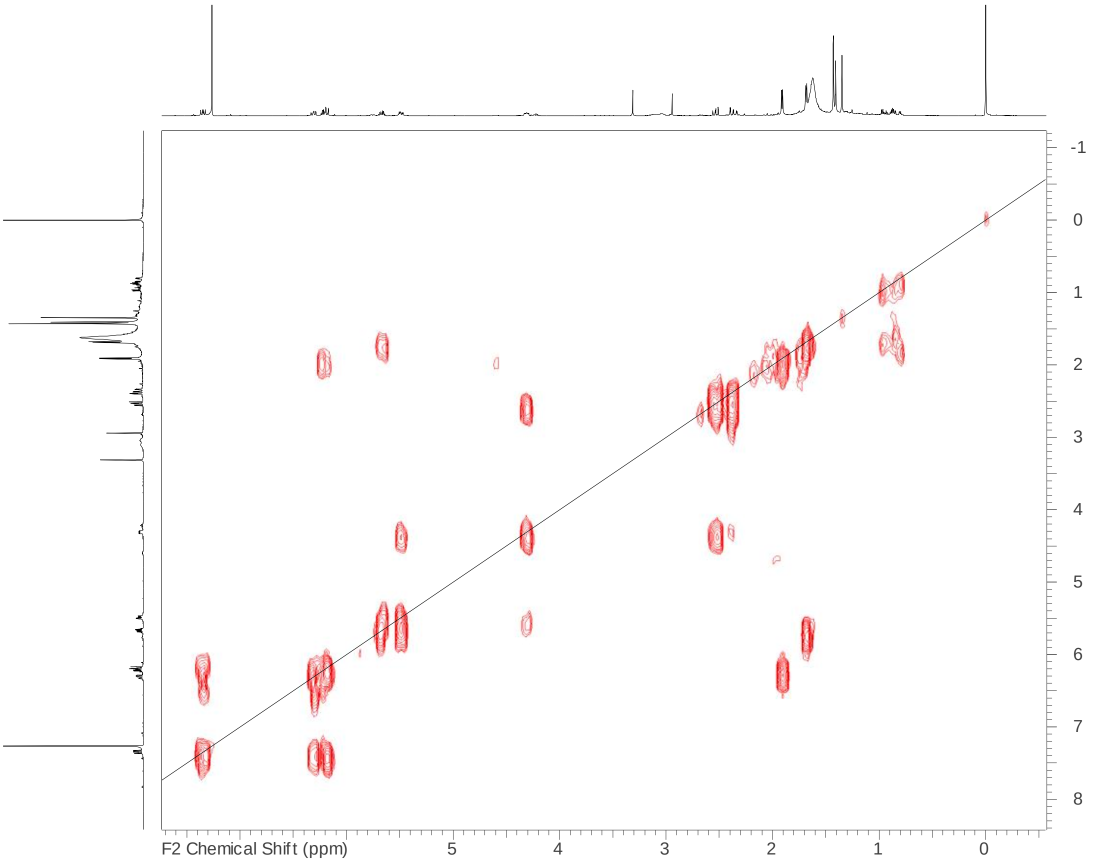

Figure S23 COSY spectrum of compound **3**

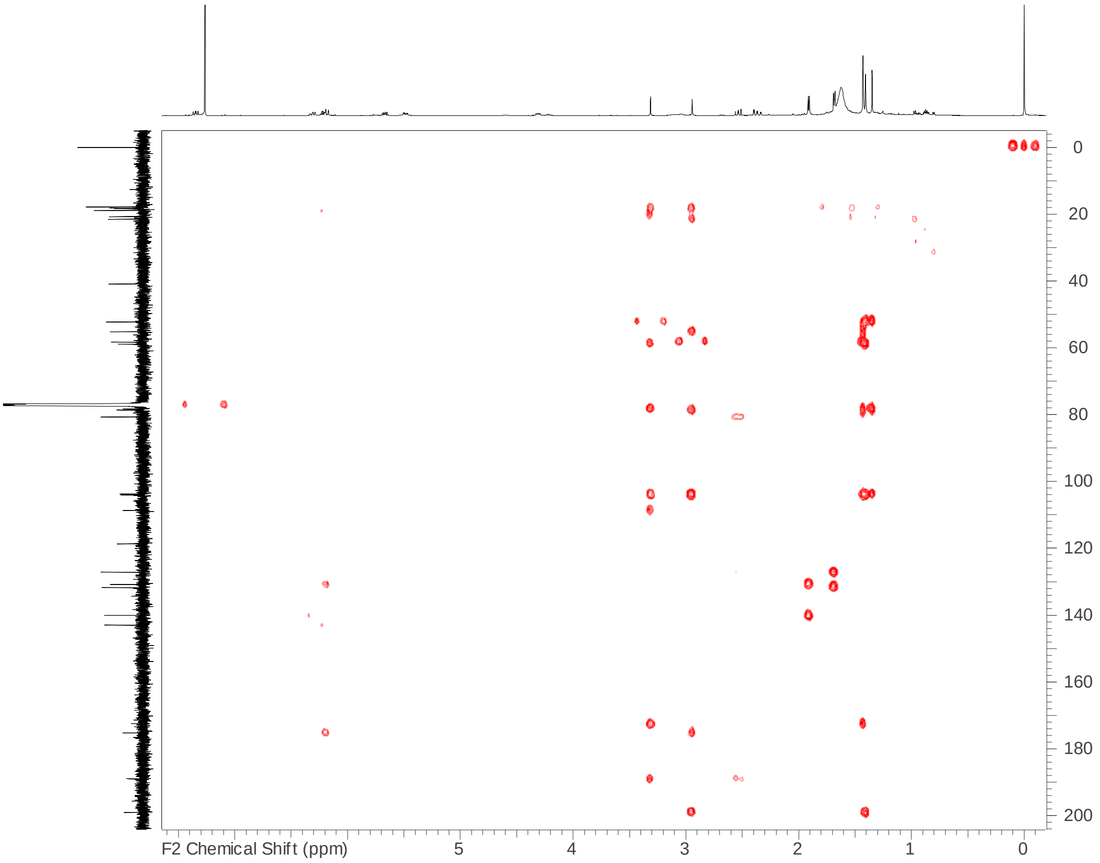

Figure S24 HMBC spectrum of compound **3**

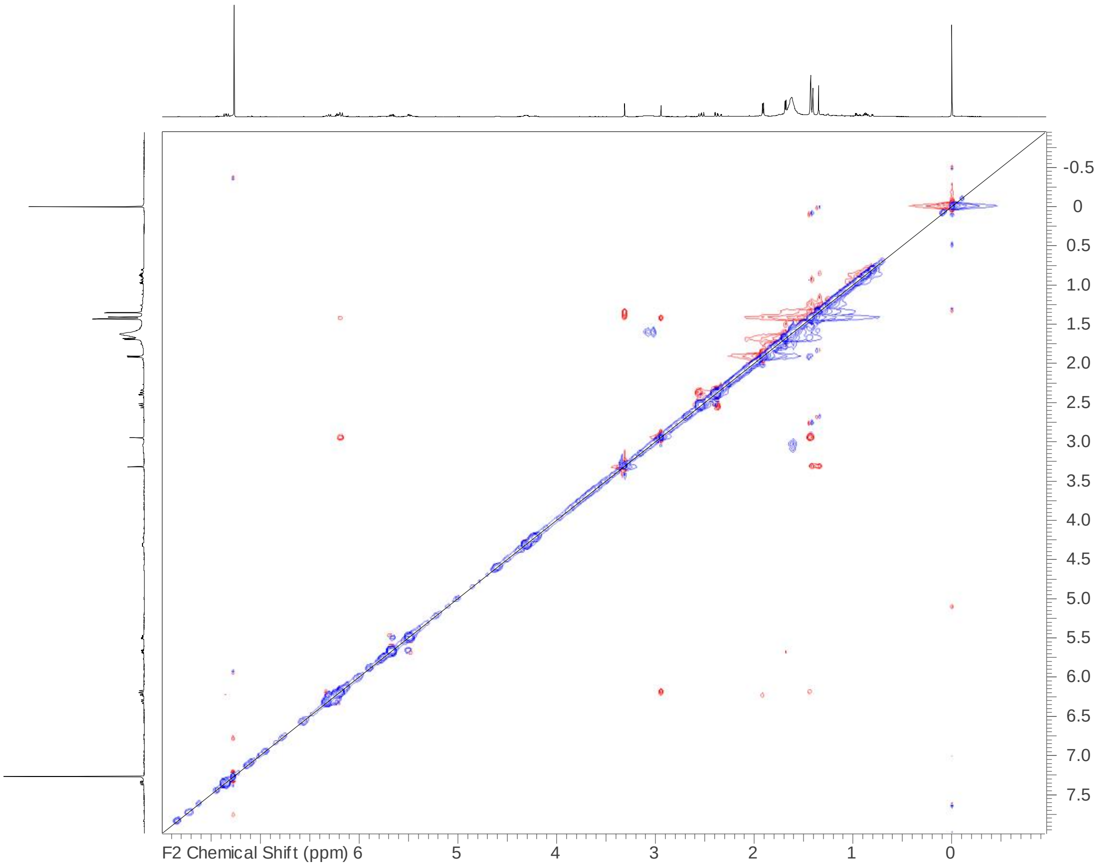

Figure S25 NOESY spectrum of compound **3**

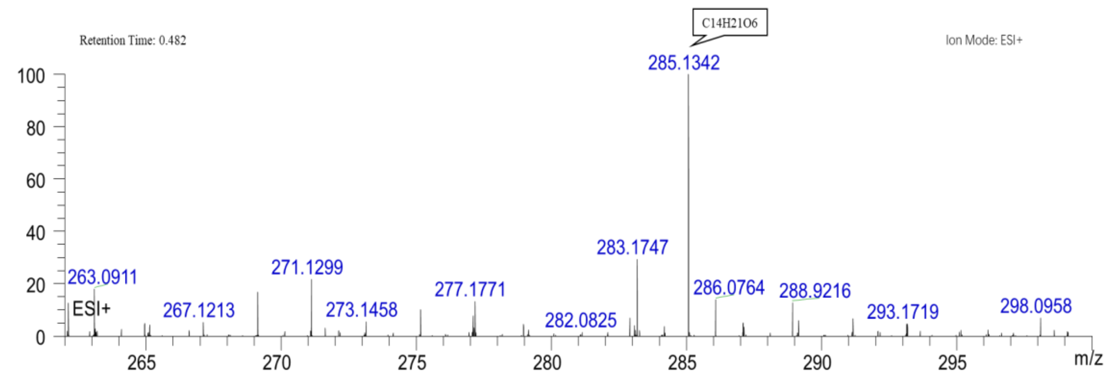

Figure S26 HR-ESI-MS spectra of compound **4**

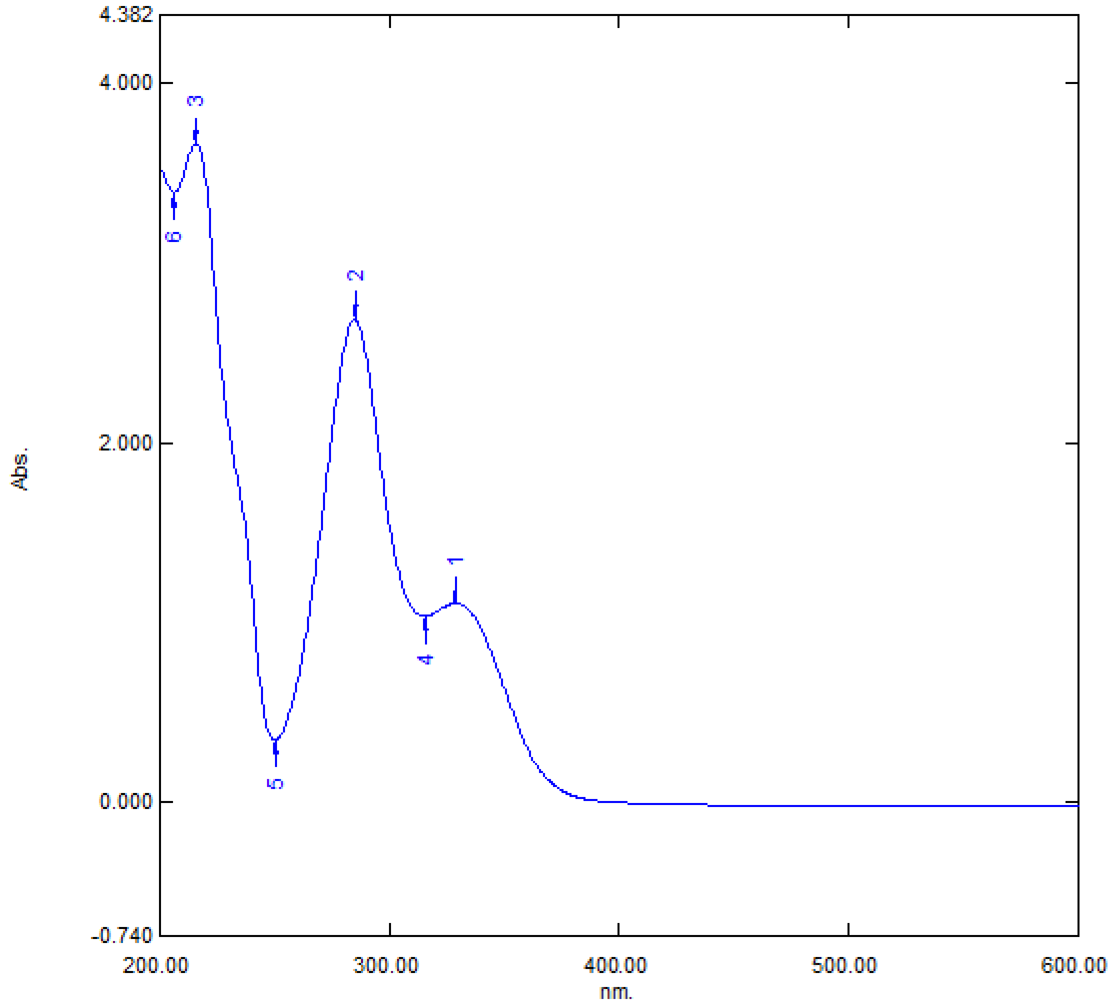

Figure S27 UV spectrum of compound **4**

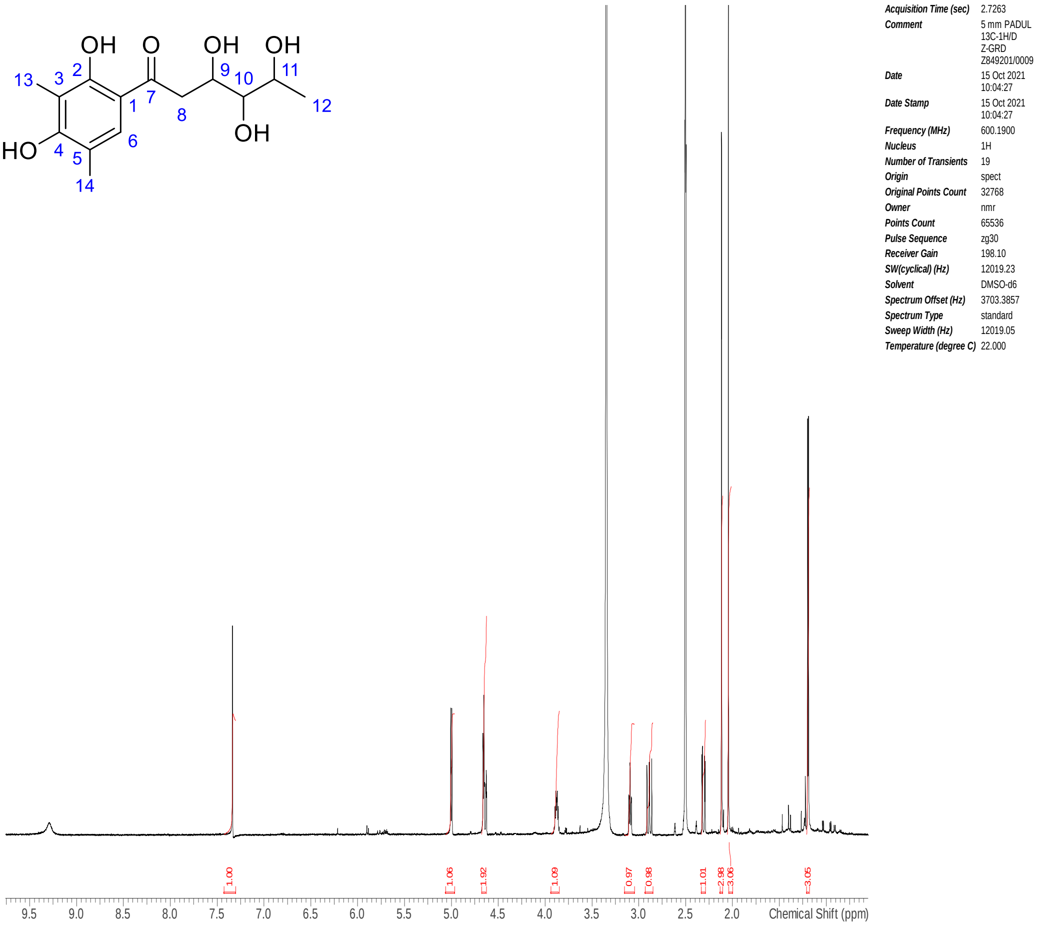

Figure S28 ^{1}H-NMR (150 MHz, DMSO-d_6
) of compound **4**

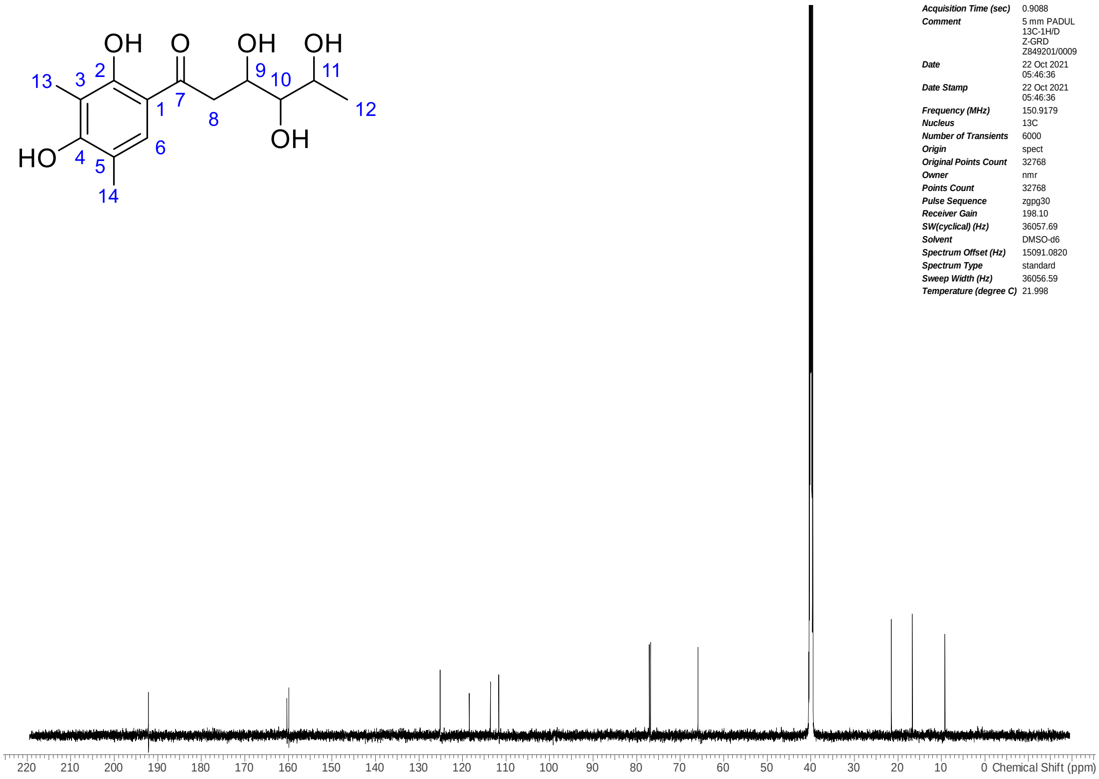

Figure S29 ^{13}C-NMR (600 MHz, DMSO-d_6) of compound **4**

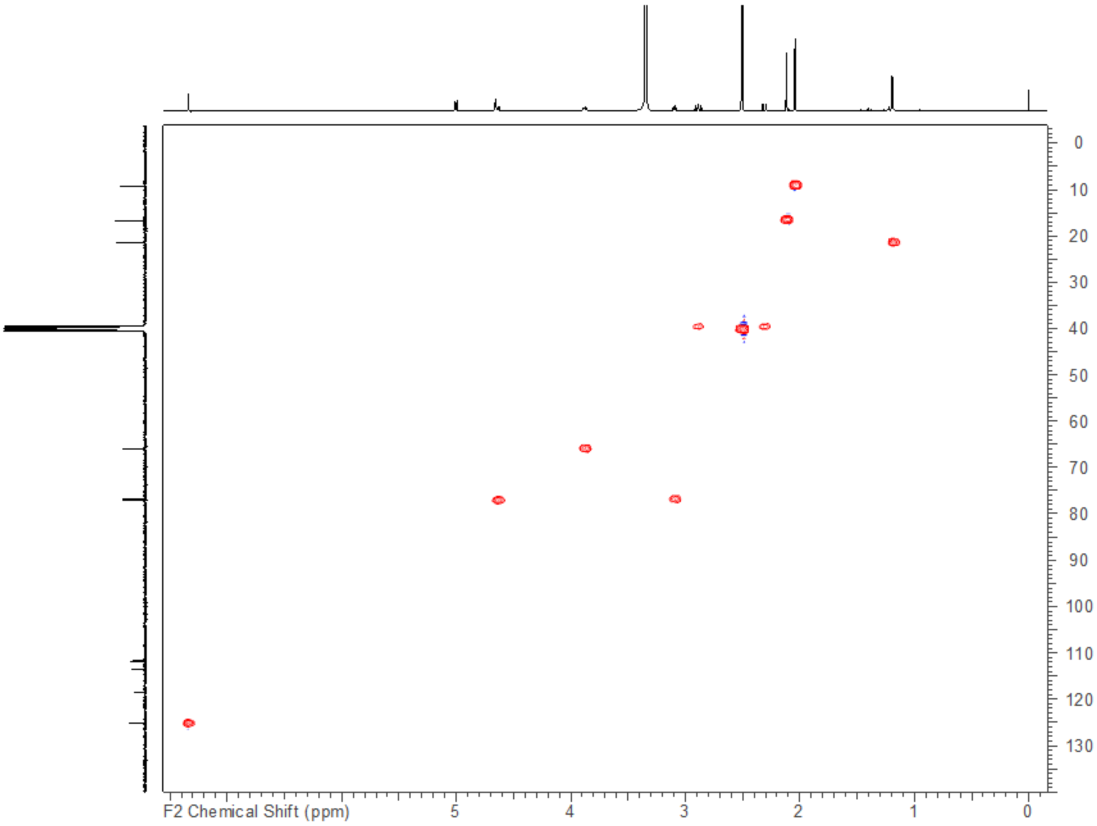

Figure S30 HSQC spectrum of compound **4**

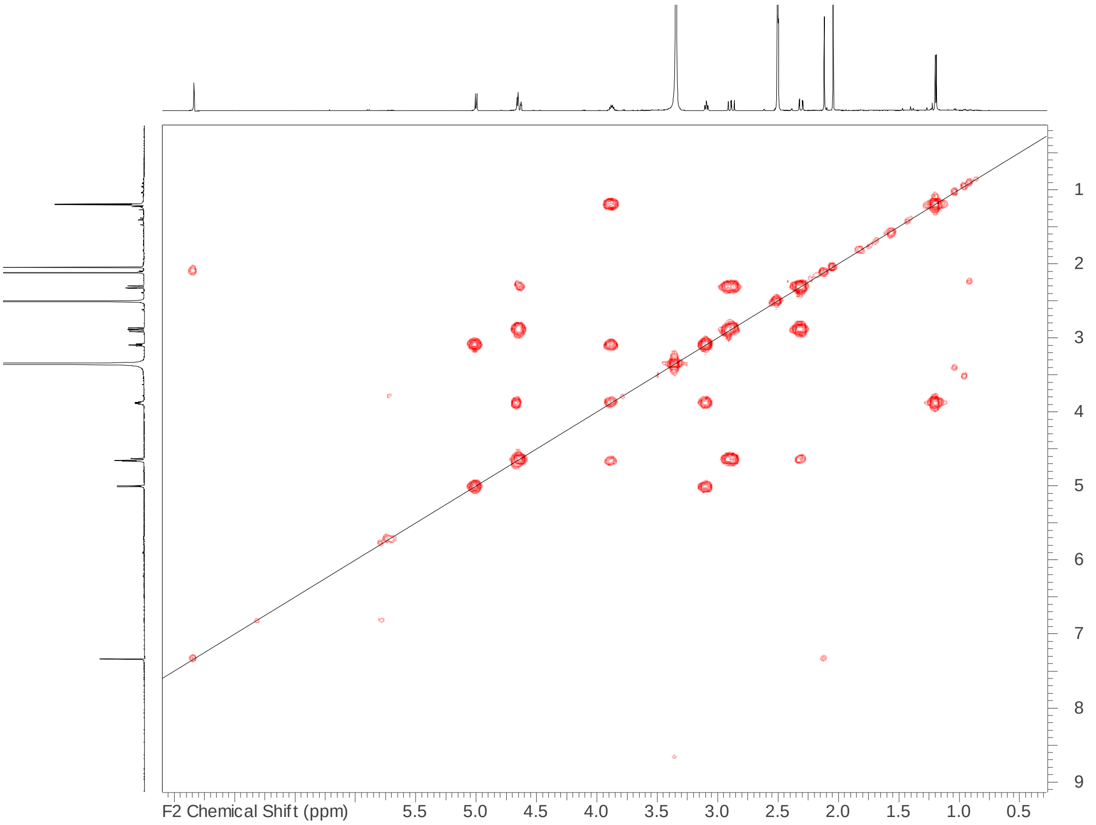

Figure S31 COSY spectrum of compound **4**

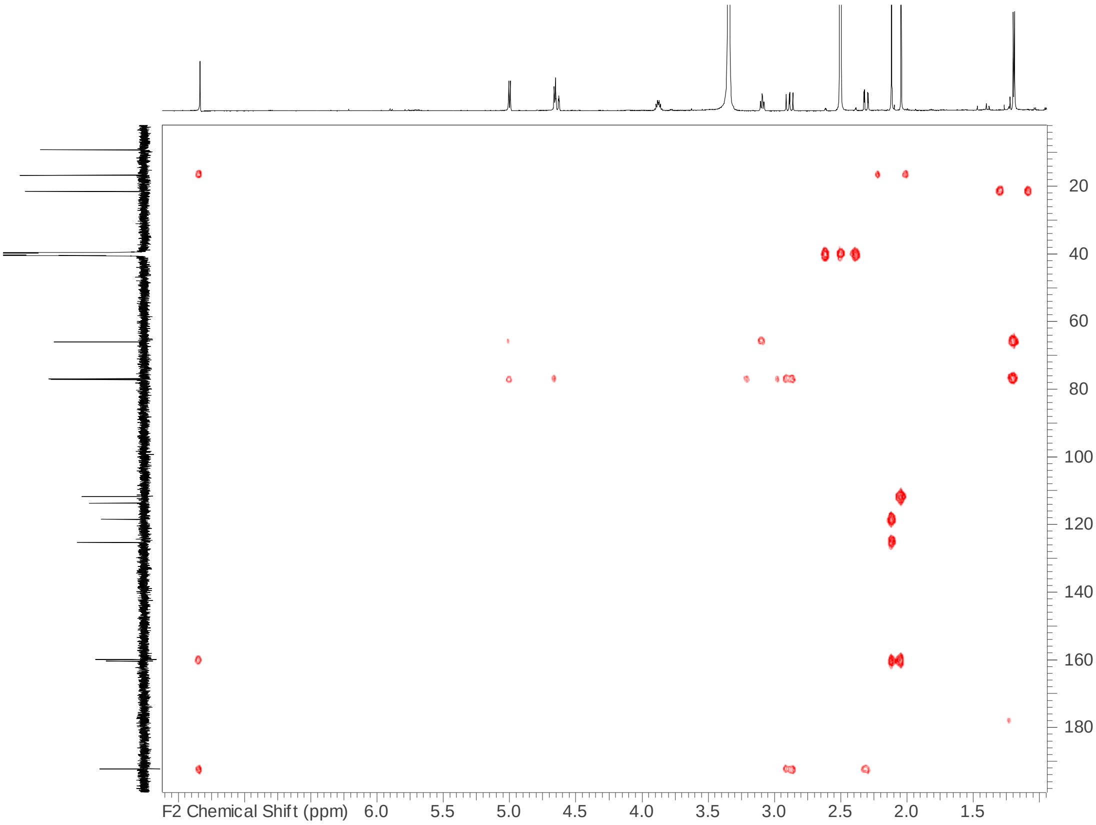

Figure S32 HMBC spectrum of compound **4**

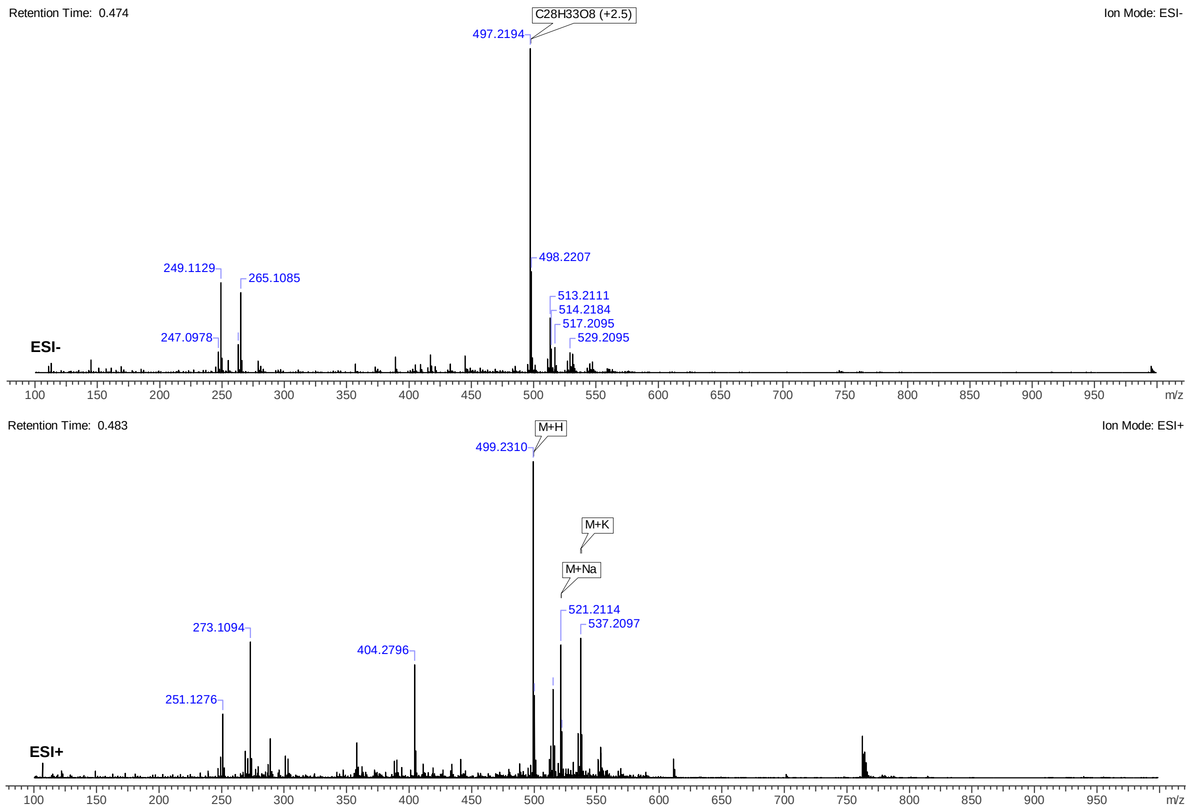

Figure S33 HR-ESI-MS spectra of compound **5**

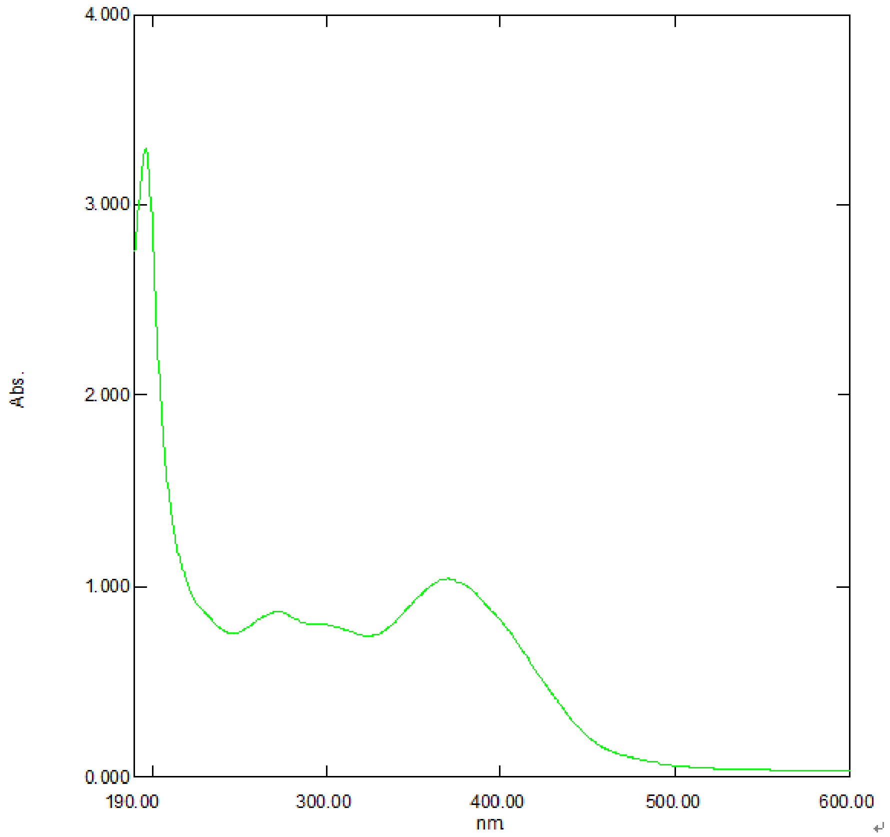

Figure S34 UV spectrum of compound **5**

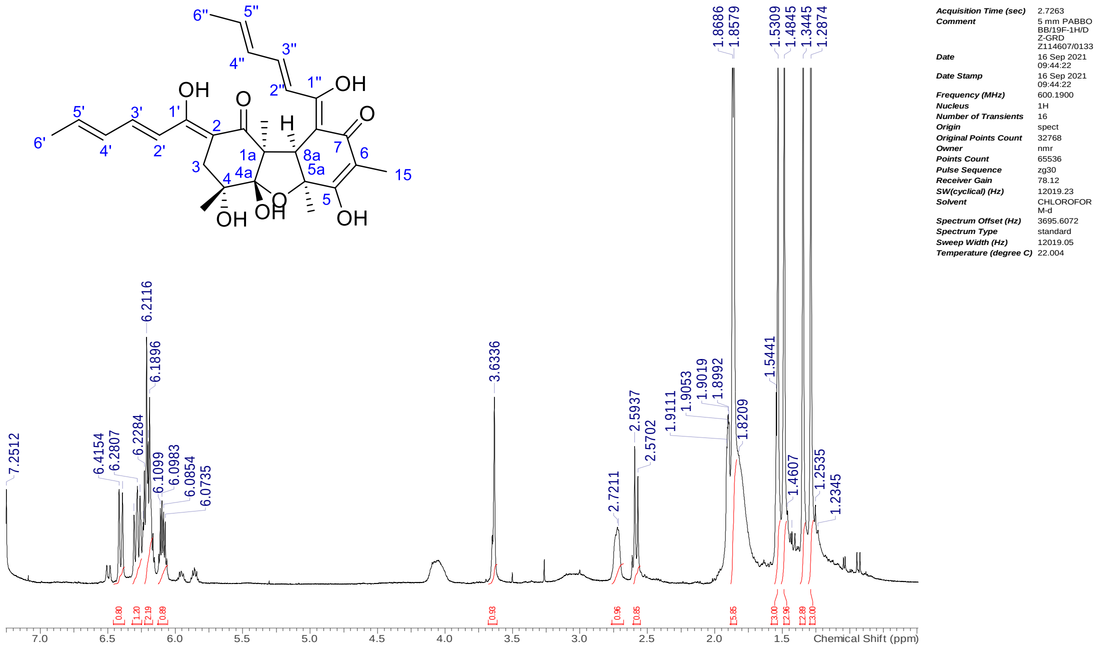

Figure S35 ^{1}H-NMR (600 MHz, $CDCl_3$) of compound **5**

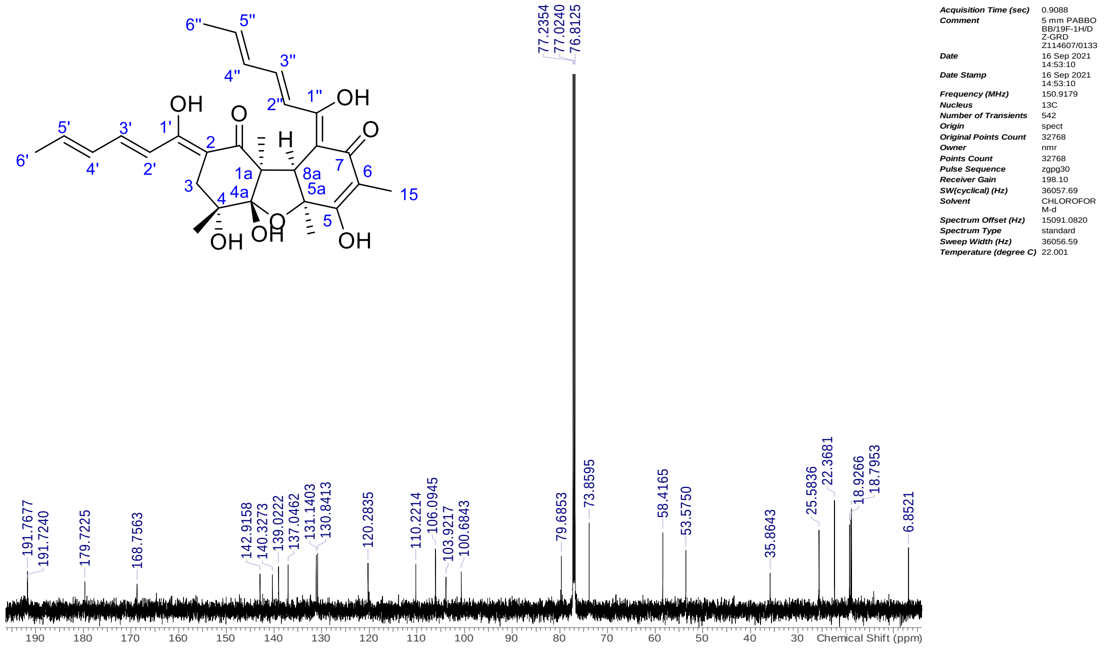

Figure S36 ^{13}C-NMR (150 MHz, $CDCl_3$) of compound **5**

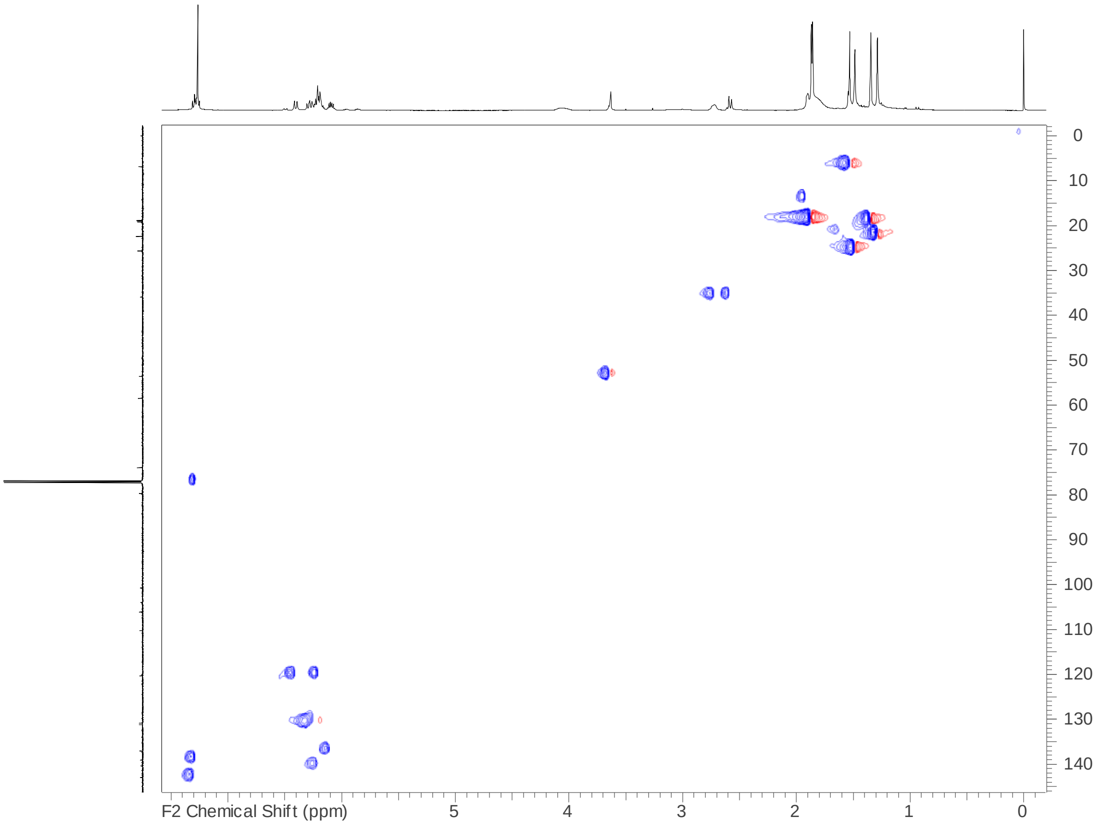

Figure S37. HSQC spectrum of compound **5**

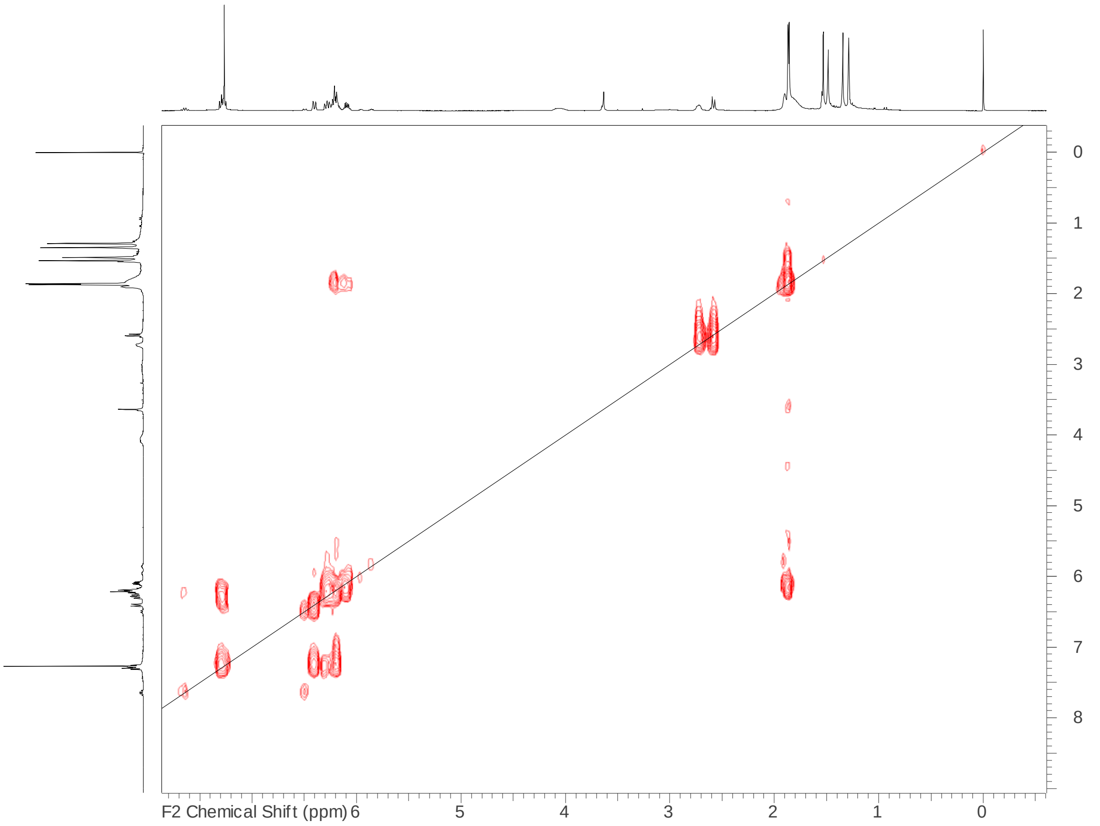

Figure S38. COSY spectrum of compound **5**

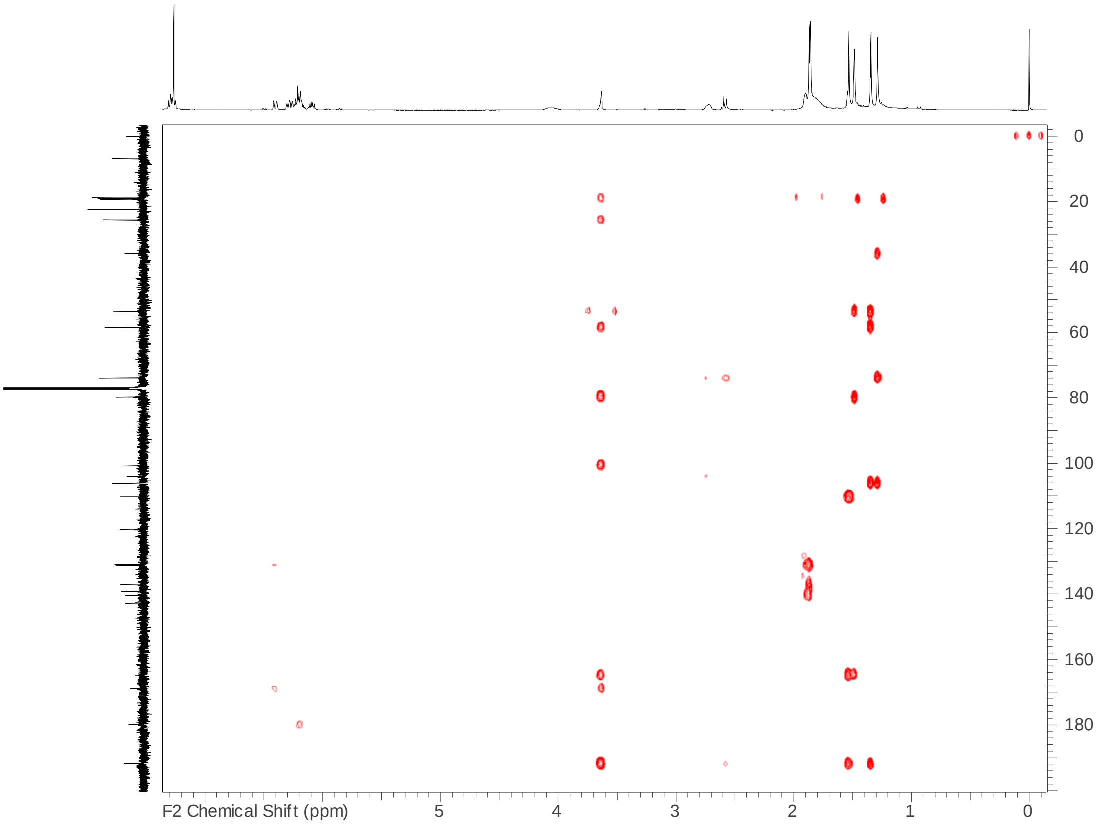

Figure S39 HMBC spectrum of compound **5**

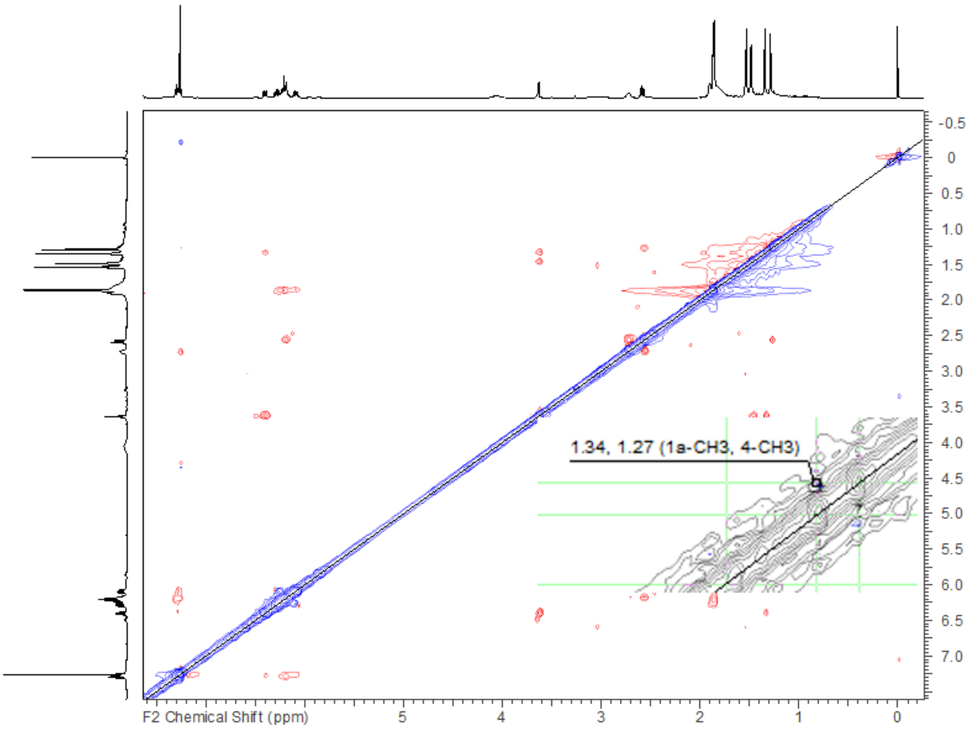

Figure S40 NOESY spectrum of compound **5**

ECD calculation of compound **1**

The conformational analysis of compound **1** was carried out by MOE 2018 using MMFF94s with an energy cutoff of 7kcal/mol. The obtained conformers were optimized using Gaussian09 at b3lyp/6-31+G(d) level in gas phase. The optimized 4 stable conformers were further subjected to ECD calculations at cam-b3lyp/6-31+g(d) level with the PCM solvation model of methanol with the first 10 electronic excitations. The overall theoretical ECD spectra were produced by SpecDis 1.70 software.

Table S1. Energy data of optimized conformers of **1**.

conformer	Energy (Hartree)	Energy (kcal/mol)	Population (%)
1	-1266.650249	-794835.025	22.28
2	-1266.65028	-794835.0444	23.02
3	-1266.650281	-794835.0452	23.06
4	-1266.65058	-794835.2327	31.64

Table S2. Standard orientations at cam-b3lyp/6-31 level in methanol.

Conformer 1-1

Center	Atomic	Atomic	Coordinates (Angstroms)		
Number	Number	Type	X	Y	Z
1	8	0	1.161624	1.917586	1.556524
2	6	0	1.768470	0.806938	2.044728
3	6	0	1.755153	-0.287631	0.952679
4	6	0	3.011584	0.028736	0.103270
5	6	0	3.030108	1.123291	-0.703397
6	6	0	1.839728	2.009489	-0.769108
7	6	0	0.674152	1.667926	0.182946
8	6	0	4.197410	1.535959	-1.563510
9	1	0	4.150117	1.042063	-2.542240
10	1	0	5.160785	1.282181	-1.109368
11	1	0	4.169199	2.613514	-1.742035
12	8	0	1.782014	2.973484	-1.518049
13	6	0	-0.523221	2.566446	-0.045248
14	1	0	-1.304984	2.368290	0.693321
15	1	0	-0.926956	2.409623	-1.048461
16	1	0	-0.220568	3.612877	0.039291
17	6	0	1.716236	-1.687426	1.591803
18	1	0	2.601940	-1.815298	2.222692
19	1	0	0.882970	-1.708691	2.303015
20	6	0	6.074946	-2.224280	-0.916131
21	1	0	6.195792	-2.967208	-1.707553
22	1	0	6.279162	-2.677996	0.061210
23	1	0	6.816447	-1.427021	-1.061255
24	6	0	4.678937	-1.631767	-0.972411
25	6	0	4.264185	-0.805894	0.255813
26	1	0	4.188820	-1.510655	1.090215
27	1	0	5.113290	-0.158527	0.517160

28	6	0	1.580747	-2.856161	0.606092
29	1	0	2.385030	-2.876422	-0.134048
30	1	0	0.641447	-2.809996	0.043227
31	1	0	1.593579	-3.808943	1.148115
32	8	0	3.942640	-1.807406	-1.923846
33	8	0	2.236118	0.743737	3.155499
34	6	0	0.479636	0.130838	0.150356
35	1	0	0.531563	-0.235224	-0.878822
36	6	0	-0.855312	-0.321331	0.760172
37	1	0	-1.090503	0.222456	1.683849
38	1	0	-0.802811	-1.379248	1.050428
39	6	0	-2.036745	-0.241246	-0.209355
40	6	0	-3.385346	-0.247814	0.402859
41	1	0	-3.450863	-0.268554	1.489979
42	6	0	-4.507045	-0.221429	-0.352421
43	1	0	-4.386888	-0.195947	-1.435481
44	6	0	-5.856024	-0.218335	0.174840
45	1	0	-5.968678	-0.250052	1.259981
46	6	0	-6.956771	-0.164944	-0.603324
47	1	0	-6.852564	-0.140491	-1.688815
48	8	0	-1.866157	-0.220219	-1.423460
49	6	0	-8.364289	-0.152424	-0.083524
50	1	0	-8.870990	0.774436	-0.375872
51	1	0	-8.372953	-0.206805	1.015325
52	8	0	-9.159741	-1.193902	-0.658494
53	1	0	-8.752615	-2.048243	-0.442020

Conformer 1-2

Center	Atomic	Atomic	Coordinates (Angstroms)		
Number	Number	Type	X	Y	Z
1	8	0	1.078997	1.797506	1.673820
2	6	0	1.731333	0.684466	2.093209
3	6	0	1.767014	-0.337486	0.933335
4	6	0	3.008798	0.085635	0.109666
5	6	0	2.982483	1.229533	-0.624891
6	6	0	1.755531	2.067081	-0.637955
7	6	0	0.604027	1.614920	0.285301
8	6	0	4.135526	1.744010	-1.448956
9	1	0	4.148892	1.267527	-2.437124
10	1	0	5.103776	1.552258	-0.973927
11	1	0	4.032261	2.820084	-1.606050
12	8	0	1.657444	3.075137	-1.321820
13	6	0	-0.630699	2.474183	0.109954
14	1	0	-1.029426	2.359660	-0.900952
15	1	0	-0.372139	3.525569	0.256267
16	1	0	-1.402002	2.200063	0.835178

17	6	0	1.789427	-1.775370	1.482333
18	1	0	2.681655	-1.904639	2.103547
19	1	0	0.960122	-1.876405	2.191414
20	6	0	6.168983	-1.961235	-1.041813
21	1	0	6.875633	-1.125680	-1.136878
22	1	0	6.321825	-2.648872	-1.876505
23	1	0	6.392296	-2.463939	-0.093126
24	6	0	4.749286	-1.425535	-1.067062
25	6	0	4.295912	-0.702698	0.211496
26	1	0	4.250571	-1.464057	0.996779
27	1	0	5.115820	-0.037764	0.518562
28	6	0	1.700158	-2.884651	0.425614
29	1	0	2.505133	-2.829024	-0.312205
30	1	0	0.760046	-2.840184	-0.135953
31	1	0	1.749549	-3.867938	0.907754
32	8	0	4.025406	-1.565853	-2.033727
33	8	0	2.198375	0.569938	3.200113
34	6	0	0.475212	0.076121	0.155022
35	1	0	0.542810	-0.221620	-0.895045
36	6	0	-0.839188	-0.469734	0.732029
37	1	0	-1.100968	0.011901	1.682658
38	1	0	-0.739512	-1.538300	0.965158
39	6	0	-2.021167	-0.390128	-0.236713
40	6	0	-3.367691	-0.517838	0.366640
41	1	0	-3.432967	-0.612088	1.449892
42	6	0	-4.487653	-0.517264	-0.391637
43	1	0	-4.367526	-0.422431	-1.470854
44	6	0	-5.834758	-0.632304	0.127728
45	1	0	-5.947776	-0.713202	1.210271
46	6	0	-6.933148	-0.651848	-0.655310
47	1	0	-6.829100	-0.563029	-1.737477
48	8	0	-1.850661	-0.273969	-1.445422
49	6	0	-8.338794	-0.771046	-0.143735
50	1	0	-8.348839	-0.823876	0.955195
51	1	0	-8.808550	-1.684449	-0.526331
52	8	0	-9.174998	0.288955	-0.617601
53	1	0	-8.801640	1.133850	-0.318661

Conformer 1-3

Center	Atomic	Atomic	Coordinates (Angstroms)		
Number	Number	Type	X	Y	Z
1	8	0	1.078807	1.797069	1.674077
2	6	0	1.731305	0.684038	2.093281
3	6	0	1.767056	-0.337728	0.933247
4	6	0	3.008790	0.085654	0.109631
5	6	0	2.982307	1.229640	-0.624776

6	6	0	1.755270	2.067064	-0.637668
7	6	0	0.603841	1.614654	0.285568
8	6	0	4.135254	1.744411	-1.448781
9	1	0	5.103657	1.551008	-0.974761
10	1	0	4.032852	2.820832	-1.604126
11	1	0	4.147474	1.269520	-2.437729
12	8	0	1.657075	3.075224	-1.321366
13	6	0	-0.630985	2.473802	0.110349
14	1	0	-1.029732	2.359348	-0.900556
15	1	0	-0.372534	3.525200	0.256771
16	1	0	-1.402234	2.199516	0.835569
17	6	0	1.789602	-1.775701	1.482009
18	1	0	2.681829	-1.905003	2.103217
19	1	0	0.960289	-1.876928	2.191054
20	6	0	6.169602	-1.960055	-1.042127
21	1	0	6.322995	-2.646895	-1.877366
22	1	0	6.392768	-2.463525	-0.093803
23	1	0	6.875974	-1.124145	-1.136128
24	6	0	4.749697	-1.424951	-1.067276
25	6	0	4.296030	-0.702498	0.211433
26	1	0	4.250650	-1.464095	0.996480
27	1	0	5.115745	-0.037499	0.518859
28	6	0	1.700465	-2.884797	0.425086
29	1	0	1.749762	-3.868171	0.907063
30	1	0	2.505560	-2.829058	-0.312594
31	1	0	0.760442	-2.840214	-0.136622
32	8	0	4.025803	-1.565448	-2.033901
33	8	0	2.198415	0.569432	3.200144
34	6	0	0.475201	0.075858	0.155028
35	1	0	0.542798	-0.221712	-0.895086
36	6	0	-0.839130	-0.470248	0.731973
37	1	0	-1.100853	0.011052	1.682783
38	1	0	-0.739360	-1.538891	0.964715
39	6	0	-2.021171	-0.390406	-0.236682
40	6	0	-3.367690	-0.517789	0.366740
41	1	0	-3.432944	-0.611822	1.450012
42	6	0	-4.487677	-0.517235	-0.391505
43	1	0	-4.367564	-0.422621	-1.470743
44	6	0	-5.834787	-0.632009	0.127900
45	1	0	-5.947812	-0.712663	1.210461
46	6	0	-6.933172	-0.651579	-0.655141
47	1	0	-6.829122	-0.562957	-1.737325
48	8	0	-1.850694	-0.274360	-1.445410
49	6	0	-8.338857	-0.770642	-0.143621
50	1	0	-8.349000	-0.822673	0.955348

51	1	0	-8.808394	-1.684434	-0.525570
52	8	0	-9.175208	0.288827	-0.618374
53	1	0	-8.802540	1.133979	-0.319305

Conformer 1-4

Center	Atomic	Atomic	Coordinates (Angstroms)		
Number	Number	Type	X	Y	Z
1	8	0	1.051089	1.797495	1.673863
2	6	0	1.648694	0.657962	2.102066
3	6	0	1.692804	-0.350900	0.931086
4	6	0	2.978902	0.038670	0.160502
5	6	0	3.019270	1.191663	-0.559233
6	6	0	1.823326	2.072043	-0.606335
7	6	0	0.622799	1.648580	0.265963
8	6	0	4.219534	1.675367	-1.333180
9	1	0	5.162329	1.446343	-0.824415
10	1	0	4.158147	2.755886	-1.481544
11	1	0	4.254574	1.208895	-2.325608
12	8	0	1.787110	3.091742	-1.279181
13	6	0	-0.574519	2.551780	0.056272
14	1	0	-0.287293	3.591407	0.230900
15	1	0	-1.383370	2.292004	0.744673
16	1	0	-0.935510	2.466300	-0.971494
17	6	0	1.645200	-1.794895	1.462193
18	1	0	2.508406	-1.962098	2.114688
19	1	0	0.786677	-1.875716	2.138265
20	6	0	6.108357	-2.107855	-0.892086
21	1	0	6.271717	-2.632612	0.056919
22	1	0	6.849393	-1.298702	-0.943552
23	1	0	6.270763	-2.788731	-1.730522
24	6	0	4.711865	-1.518038	-0.968481
25	6	0	4.233326	-0.794995	0.300386
26	1	0	4.131413	-1.563928	1.072932
27	1	0	5.063157	-0.163426	0.648492
28	6	0	1.558236	-2.888567	0.389081
29	1	0	2.390444	-2.851134	-0.319246
30	1	0	0.640552	-2.807089	-0.204431
31	1	0	1.558525	-3.878365	0.860454
32	8	0	4.024724	-1.618904	-1.966380
33	8	0	2.070919	0.514366	3.223710
34	6	0	0.446827	0.116813	0.109565
35	1	0	0.545610	-0.168941	-0.941383
36	6	0	-0.907418	-0.391067	0.624670
37	1	0	-1.192898	0.087590	1.569930
38	1	0	-0.853321	-1.465015	0.846875
39	6	0	-2.044715	-0.258616	-0.392654

40	6	0	-3.415525	-0.361112	0.149640
41	1	0	-3.530490	-0.478139	1.226338
42	6	0	-4.503537	-0.310927	-0.654109
43	1	0	-4.332334	-0.191304	-1.724301
44	6	0	-5.872983	-0.402691	-0.196481
45	1	0	-6.055399	-0.518613	0.869563
46	6	0	-6.922735	-0.349332	-1.039317
47	1	0	-6.752979	-0.232795	-2.110296
48	8	0	-1.813951	-0.122678	-1.590539
49	6	0	-8.360287	-0.437662	-0.635962
50	1	0	-8.827902	-1.287504	-1.162845
51	1	0	-8.883279	0.473381	-0.974830
52	8	0	-8.469335	-0.591936	0.773189
53	1	0	-9.405906	-0.635707	1.015624

ECD calculation of compound **2**

Table S3. Energy data of optimized conformers of 2 (17*R*-OH,13*S*-OH).

Name	Compound	conformer	Energy (Hartree)	Energy (kcal/mol)	Population (%)
2-1	2	1	-1193.84	-749148	79.35
2-2	2	2	-1193.84	-749147	16.79
2-3	2	3	-1193.84	-749146	3.35
2-4	2	4	-1193.84	-749145	0.21
2-5	2	5	-1193.84	-749145	0.31

Standard orientations at cam-b3lyp/6-31 level in methanol.

Conformer 2-1

Center Number	Atomic Number	Atomic Type	Coordinates (Angstroms) X	Y	Z
1	6	0	8.604206	-0.423236	-0.949930
2	1	0	9.406833	-0.060861	-0.292517
3	1	0	8.883903	-0.134087	-1.972588
4	1	0	8.588228	-1.517382	-0.897552
5	6	0	7.281709	0.169257	-0.564595
6	1	0	7.214256	1.258915	-0.591988
7	6	0	6.193109	-0.532373	-0.197055
8	1	0	6.245921	-1.622155	-0.167395
9	6	0	4.920496	0.071658	0.170111
10	1	0	4.875285	1.162590	0.134682
11	6	0	3.819409	-0.612794	0.536005
12	1	0	3.844737	-1.703142	0.583905
13	6	0	2.516128	0.030131	0.917317
14	1	0	2.609104	1.118920	0.807971
15	6	0	1.344969	-0.495699	0.071383
16	1	0	1.591889	-0.386089	-0.990831
17	1	0	1.289174	-1.569789	0.263766
18	8	0	2.173569	-0.275507	2.287606
19	1	0	2.929655	-0.041051	2.848830
20	6	0	-0.007604	0.176466	0.397789
21	1	0	-0.028103	0.422504	1.463493
22	6	0	-1.279039	-0.663639	0.026227
23	6	0	-2.556724	0.008584	0.609751
24	6	0	-2.597721	1.367320	0.728319
25	6	0	-1.461336	2.184328	0.233558
26	6	0	-0.302811	1.432626	-0.456061
27	8	0	-0.833549	0.862070	-1.714804
28	6	0	-1.384290	-0.346471	-1.477115
29	8	0	-1.905916	-1.005208	-2.352767
30	8	0	-1.435323	3.402436	0.350419
31	6	0	0.821000	2.373759	-0.841578

32	1	0	1.242104	2.850505	0.047850
33	1	0	0.428680	3.166756	-1.482997
34	1	0	1.609279	1.843149	-1.380724
35	6	0	-3.731211	2.187365	1.295590
36	1	0	-4.268477	2.715436	0.497712
37	1	0	-3.329349	2.961620	1.956592
38	1	0	-4.454737	1.592291	1.854285
39	6	0	-3.756887	-0.855551	0.938973
40	1	0	-4.374034	-0.336887	1.678086
41	1	0	-3.448268	-1.793988	1.403457
42	6	0	-4.714830	-1.247191	-0.239648
43	1	0	-5.566073	-1.735115	0.250700
44	6	0	-5.238598	-0.058068	-1.046675
45	1	0	-4.430860	0.469005	-1.567488
46	1	0	-5.753806	0.662704	-0.400920
47	1	0	-5.948990	-0.416273	-1.799022
48	8	0	-4.175051	-2.261823	-1.076725
49	1	0	-3.497329	-1.884308	-1.672554
50	6	0	-1.173704	-2.176533	0.307331
51	1	0	-2.098026	-2.663139	-0.012554
52	1	0	-0.396085	-2.594009	-0.341828
53	6	0	-0.862775	-2.530710	1.770195
54	1	0	-1.623804	-2.146873	2.459154
55	1	0	0.100599	-2.130336	2.101927
56	1	0	-0.832527	-3.620030	1.889361

Conformer 2-2

Center	Atomic	Atomic	Coordinates (Angstroms)		
Number	Number	Type	X	Y	Z
1	6	0	-8.431297	-1.227798	0.434306
2	1	0	-8.584835	-2.301294	0.612922
3	1	0	-9.249449	-0.900404	-0.222447
4	1	0	-8.534167	-0.705764	1.392107
5	6	0	-7.094681	-0.970054	-0.196035
6	1	0	-6.912991	-1.453317	-1.158316
7	6	0	-6.122037	-0.203147	0.330931
8	1	0	-6.292541	0.284286	1.292916
9	6	0	-4.829821	0.026783	-0.300728
10	1	0	-4.659847	-0.460873	-1.261177
11	6	0	-3.851821	0.788088	0.223432
12	1	0	-4.006293	1.271623	1.189948
13	6	0	-2.503307	1.039534	-0.407353
14	1	0	-2.278464	2.112961	-0.319211
15	6	0	-1.398455	0.225778	0.294860
16	1	0	-1.482125	0.361331	1.379557
17	1	0	-1.637362	-0.822029	0.101574

18	8	0	-2.449084	0.666893	-1.789789
19	1	0	-3.098898	1.196400	-2.278632
20	6	0	0.028002	0.559929	-0.194446
21	1	0	-0.018242	0.819827	-1.256336
22	6	0	1.090174	-0.573170	0.025288
23	6	0	2.417143	-0.216435	-0.708074
24	6	0	2.775453	1.094195	-0.834567
25	6	0	1.937635	2.150132	-0.212490
26	6	0	0.719209	1.685065	0.612384
27	8	0	1.236920	0.971435	1.801015
28	6	0	1.443701	-0.328963	1.504457
29	8	0	1.885730	-1.116081	2.315247
30	8	0	2.195330	3.340850	-0.330720
31	6	0	-0.089115	2.860419	1.124985
32	1	0	-0.915997	2.522353	1.754205
33	1	0	-0.480600	3.448882	0.290681
34	1	0	0.554744	3.517269	1.715019
35	6	0	4.005733	1.626123	-1.529396
36	1	0	4.749953	1.965328	-0.797712
37	1	0	3.740871	2.504341	-2.126123
38	1	0	4.482659	0.892171	-2.180608
39	6	0	3.321535	-1.338012	-1.175568
40	1	0	3.957875	-0.966068	-1.983715
41	1	0	2.739210	-2.158922	-1.598313
42	6	0	4.284120	-1.985218	-0.118950
43	1	0	4.924254	-2.654321	-0.706837
44	6	0	5.176406	-0.983392	0.615494
45	1	0	4.589932	-0.289517	1.228913
46	1	0	5.773600	-0.393347	-0.089420
47	1	0	5.859764	-1.525324	1.277530
48	8	0	3.611868	-2.857110	0.780238
49	1	0	3.126039	-2.339797	1.453334
50	6	0	0.585854	-2.007452	-0.233927
51	1	0	1.394750	-2.711603	-0.025606
52	1	0	-0.187899	-2.239713	0.505918
53	6	0	0.027462	-2.238482	-1.647104
54	1	0	0.771657	-2.032240	-2.424905
55	1	0	-0.842381	-1.609037	-1.860679
56	1	0	-0.280693	-3.284638	-1.757521

Conformer 2-3

Center	Atomic	Atomic	Coordinates (Angstroms)		
Number	Number	Type	X	Y	Z
1	6	0	7.940010	-1.511056	-0.892954
2	1	0	8.527422	-0.731055	-0.396178
3	1	0	8.231271	-1.535838	-1.952276

4	1	0	8.230050	-2.481448	-0.466473
5	6	0	6.466596	-1.274708	-0.744250
6	1	0	5.810158	-2.011060	-1.212777
7	6	0	5.907709	-0.239901	-0.089231
8	1	0	6.552606	0.504173	0.381879
9	6	0	4.471374	-0.035572	0.038340
10	1	0	3.840561	-0.784921	-0.440198
11	6	0	3.910863	1.003488	0.687719
12	1	0	4.560182	1.744628	1.156868
13	6	0	2.441562	1.275675	0.884555
14	1	0	2.224794	2.280082	0.506536
15	6	0	1.492352	0.259815	0.223630
16	1	0	1.753141	0.138921	-0.834755
17	1	0	1.676765	-0.709514	0.694777
18	8	0	2.154719	1.392486	2.296615
19	1	0	2.602559	0.662612	2.755785
20	6	0	0.004530	0.646590	0.366651
21	1	0	-0.137205	1.139260	1.333678
22	6	0	-1.014102	-0.537099	0.218258
23	6	0	-2.447677	-0.067028	0.604685
24	6	0	-2.807841	1.227723	0.368925
25	6	0	-1.861215	2.142750	-0.318220
26	6	0	-0.516605	1.544329	-0.782870
27	8	0	-0.818504	0.567902	-1.855147
28	6	0	-1.089669	-0.638348	-1.317083
29	8	0	-1.384803	-1.600955	-1.995084
30	8	0	-2.128475	3.318207	-0.527612
31	6	0	0.382096	2.600449	-1.394409
32	1	0	0.605430	3.382265	-0.663553
33	1	0	-0.133200	3.075201	-2.233066
34	1	0	1.314312	2.162025	-1.758937
35	6	0	-4.136710	1.864776	0.695674
36	1	0	-4.730618	1.282837	1.401954
37	1	0	-4.734005	2.007825	-0.213774
38	1	0	-3.972856	2.862516	1.114080
39	6	0	-3.435923	-1.082989	1.140333
40	1	0	-4.203905	-0.558918	1.716283
41	1	0	-2.950593	-1.772542	1.833957
42	6	0	-4.196494	-1.977273	0.100570
43	1	0	-4.941280	-2.517410	0.697762
44	6	0	-4.927874	-1.193636	-0.990489
45	1	0	-5.636370	-0.478064	-0.557467
46	1	0	-5.485792	-1.891182	-1.623819
47	1	0	-4.231760	-0.638702	-1.630111
48	8	0	-3.380182	-3.007579	-0.442100

49	1	0	-2.775020	-2.638608	-1.116125
50	6	0	-0.584184	-1.864881	0.873953
51	1	0	-1.348817	-2.621075	0.681334
52	1	0	0.310540	-2.230523	0.358133
53	6	0	-0.301723	-1.768254	2.380856
54	1	0	0.514698	-1.073982	2.606455
55	1	0	-0.022674	-2.753029	2.772873
56	1	0	-1.174895	-1.424064	2.945906

Conformer 2-4

Center	Atomic	Atomic	Coordinates (Angstroms)		
Number	Number	Type	X	Y	Z
1	6	0	7.244288	-2.224701	1.105048
2	1	0	7.434581	-3.172184	0.581857
3	1	0	6.734445	-2.452326	2.047663
4	1	0	8.228037	-1.796449	1.342680
5	6	0	6.440219	-1.289693	0.252037
6	1	0	6.880323	-1.011006	-0.707685
7	6	0	5.237086	-0.780969	0.578143
8	1	0	4.785132	-1.052732	1.533828
9	6	0	4.478159	0.124157	-0.272648
10	1	0	4.940173	0.394537	-1.225029
11	6	0	3.266180	0.630480	0.028330
12	1	0	2.802771	0.381508	0.983917
13	6	0	2.517171	1.590117	-0.857101
14	1	0	3.124825	1.770756	-1.757778
15	6	0	1.145319	1.089809	-1.355981
16	1	0	0.780582	1.802084	-2.104303
17	1	0	1.350809	0.166386	-1.902078
18	8	0	2.311287	2.837200	-0.169104
19	1	0	3.163394	3.121568	0.198878
20	6	0	0.062088	0.846784	-0.274971
21	1	0	0.539630	0.716400	0.700796
22	6	0	-0.899820	-0.370791	-0.545492
23	6	0	-1.752301	-0.669260	0.723588
24	6	0	-2.102005	0.362228	1.544833
25	6	0	-1.738761	1.756117	1.176263
26	6	0	-1.010902	1.965330	-0.170664
27	8	0	-1.971560	1.608331	-1.242783
28	6	0	-1.921834	0.288308	-1.491390
29	8	0	-2.633850	-0.248153	-2.316363
30	8	0	-2.013940	2.706143	1.895781
31	6	0	-0.644604	3.417828	-0.384724
32	1	0	-1.544581	4.031089	-0.293768
33	1	0	-0.208325	3.569991	-1.373646
34	1	0	0.077815	3.741370	0.364921

35	6	0	-2.898724	0.262211	2.823286
36	1	0	-2.962225	-0.755049	3.212473
37	1	0	-3.920536	0.633982	2.675374
38	1	0	-2.449172	0.903379	3.587763
39	6	0	-2.258241	-2.078766	0.957912
40	1	0	-2.465371	-2.205941	2.024352
41	1	0	-1.495430	-2.817785	0.704928
42	6	0	-3.559674	-2.520539	0.201414
43	1	0	-3.808513	-3.497178	0.634355
44	6	0	-4.753530	-1.585203	0.400900
45	1	0	-4.991703	-1.467807	1.464464
46	1	0	-5.629464	-2.005145	-0.104411
47	1	0	-4.566229	-0.589550	-0.017626
48	8	0	-3.331438	-2.803818	-1.173009
49	1	0	-3.249526	-1.971099	-1.680140
50	6	0	-0.261696	-1.622442	-1.181341
51	1	0	-1.044021	-2.367660	-1.341046
52	1	0	0.086456	-1.365042	-2.187755
53	6	0	0.898014	-2.237913	-0.385218
54	1	0	1.215370	-3.175930	-0.855277
55	1	0	0.617301	-2.467176	0.649374
56	1	0	1.770871	-1.579165	-0.346610

Conformer 2-5

Center	Atomic	Atomic	Coordinates (Angstroms)		
Number	Number	Type	X	Y	Z
1	6	0	8.270749	-1.271578	-0.498634
2	1	0	8.957498	-1.327117	0.357324
3	1	0	8.703579	-0.593206	-1.242128
4	1	0	8.235772	-2.278927	-0.936909
5	6	0	6.904184	-0.824710	-0.069596
6	1	0	6.412570	-1.439003	0.686605
7	6	0	6.269317	0.264920	-0.536291
8	1	0	6.792953	0.902336	-1.250833
9	6	0	4.926469	0.717414	-0.150724
10	1	0	4.750562	1.791213	-0.247342
11	6	0	3.905930	-0.051101	0.271837
12	1	0	4.019210	-1.133980	0.338989
13	6	0	2.566977	0.492414	0.687860
14	1	0	2.551756	1.576672	0.515134
15	6	0	1.413240	-0.190755	-0.063446
16	1	0	1.588785	-0.105384	-1.142197
17	1	0	1.483374	-1.254960	0.175530
18	8	0	2.325507	0.236901	2.089816
19	1	0	3.076218	0.587121	2.595161
20	6	0	0.015141	0.346674	0.312776

21	1	0	0.020103	0.625708	1.370481
22	6	0	-1.174767	-0.636757	0.031810
23	6	0	-2.488546	-0.085031	0.660804
24	6	0	-2.671543	1.264809	0.741080
25	6	0	-1.654970	2.181106	0.165612
26	6	0	-0.456502	1.534260	-0.560332
27	8	0	-0.982260	0.867891	-1.773239
28	6	0	-1.387288	-0.383399	-1.472611
29	8	0	-1.877665	-1.123250	-2.300314
30	8	0	-1.754213	3.398410	0.245827
31	6	0	0.539436	2.575439	-1.030023
32	1	0	0.946488	3.125791	-0.177255
33	1	0	0.034630	3.298475	-1.675421
34	1	0	1.355572	2.112864	-1.590635
35	6	0	-3.862872	1.975059	1.337620
36	1	0	-4.521259	2.363029	0.549872
37	1	0	-3.523715	2.843667	1.909817
38	1	0	-4.459644	1.336765	1.991176
39	6	0	-3.569703	-1.059899	1.079942
40	1	0	-4.205038	-0.580919	1.830446
41	1	0	-3.137752	-1.939484	1.561284
42	6	0	-4.533087	-1.601925	-0.031924
43	1	0	-5.297193	-2.163826	0.519007
44	6	0	-5.230358	-0.512188	-0.848381
45	1	0	-5.792500	0.169633	-0.199596
46	1	0	-5.930893	-0.978416	-1.548922
47	1	0	-4.516175	0.082594	-1.429681
48	8	0	-3.922699	-2.580061	-0.863955
49	1	0	-3.323889	-2.150408	-1.507048
50	6	0	-0.894384	-2.119719	0.348098
51	1	0	-1.775944	-2.711056	0.089935
52	1	0	-0.109558	-2.474202	-0.329255
53	6	0	-0.476553	-2.392407	1.801607
54	1	0	0.455057	-1.884629	2.071421
55	1	0	-0.326711	-3.468379	1.948369
56	1	0	-1.238424	-2.066840	2.519084

ECD calculation of compound **3**

Table S4. Energy data of optimized conformers of 3 (9’R-OH).

Name	Compound	conformer	Energy (Hartree)	Energy (kcal/mol)	Population (%)
3-1	3	1	-1763.64	-1106702	59.53
3-2	3	2	-1763.64	-1106701	9.96
3-3	3	3	-1763.64	-1106701	14.01
3-4	3	4	-1763.64	-1106701	8.96
3-5	3	5	-1763.64	-1106700	1.98
3-6	3	6	-1763.64	-1106699	1.48
3-7	3	7	-1763.64	-1106700	4.08

Standard orientations at cam-b3lyp/6-31 level in methanol.

Conformer 2-1

Center	Atomic	Atomic	Coordinates (Angstroms)		
Number	Number	Type	X	Y	Z
1	6	0	-2.988978	-0.962438	-1.226469
2	6	0	-2.892721	-1.661134	0.210345
3	8	0	-3.818282	-0.979630	1.067706
4	6	0	-3.221319	0.307927	1.235058
5	6	0	-3.268646	1.014633	-0.200619
6	8	0	-3.931191	0.102452	-1.089891
7	6	0	-1.779315	-0.039107	1.729815
8	6	0	-0.733256	1.045329	1.421621
9	6	0	-0.749045	1.588529	0.057460
10	6	0	-1.864171	1.151625	-0.864909
11	6	0	-1.688385	-0.219011	-1.668470
12	6	0	-0.378365	-0.925946	-1.298505
13	6	0	-0.317312	-1.524638	0.044630
14	6	0	-1.531379	-1.398734	0.929918

15	1	0	-1.964532	1.914922	-1.637047
16	6	0	-4.040952	2.325668	-0.203416
17	1	0	-4.072897	2.722563	-1.222971
18	1	0	-3.562648	3.061234	0.449607
19	1	0	-5.074543	2.187685	0.131760
20	8	0	-3.425956	-1.823189	-2.231607
21	1	0	-4.357549	-2.039078	-2.061746
22	6	0	-1.714666	0.065606	-3.178168
23	1	0	-0.899032	0.744005	-3.438707
24	1	0	-2.667814	0.526794	-3.452425
25	1	0	-1.595235	-0.852435	-3.758049
26	1	0	-1.444382	-2.161231	1.703896
27	6	0	-3.262282	-3.137301	0.190128
28	1	0	-3.223871	-3.532323	1.210261
29	1	0	-2.569978	-3.704696	-0.438502
30	1	0	-4.277364	-3.295030	-0.189396
31	8	0	-3.917362	1.003228	2.220316
32	1	0	-4.862408	0.986620	1.997259
33	6	0	-1.785283	-0.335951	3.237210
34	1	0	-2.570527	-1.061186	3.470111
35	1	0	-1.964593	0.569772	3.819732
36	1	0	-0.816752	-0.745422	3.535716
37	8	0	0.049510	1.393150	2.308911
38	8	0	0.553082	-0.939156	-2.105941
39	6	0	0.787314	-2.205089	0.503399

40	6	0	1.992930	-2.504535	-0.254755
41	1	0	1.907109	-2.402182	-1.329825
42	6	0	3.181028	-2.859142	0.298367
43	1	0	3.314639	-2.865485	1.382679
44	6	0	4.358426	-3.196469	-0.477049
45	1	0	4.251050	-3.178898	-1.562471
46	6	0	5.549078	-3.525598	0.064711
47	1	0	5.640659	-3.540077	1.152903
48	6	0	6.780892	-3.883960	-0.709781
49	1	0	6.606380	-3.843203	-1.790340
50	1	0	7.125978	-4.896197	-0.456455
51	1	0	7.610272	-3.203703	-0.470247
52	8	0	0.740593	-2.643366	1.799693
53	1	0	1.439804	-3.299768	1.945552
54	6	0	0.206299	2.448108	-0.420566
55	6	0	1.404365	2.973440	0.336558
56	1	0	1.202609	4.011534	0.642984
57	1	0	1.524489	2.395778	1.252469
58	6	0	2.711301	2.956523	-0.471435
59	1	0	2.864542	1.956905	-0.900865
60	6	0	3.903075	3.345553	0.353896
61	1	0	3.846736	4.321987	0.838231
62	6	0	4.986383	2.574343	0.508819
63	1	0	5.009575	1.600620	0.014013
64	6	0	6.190398	2.924739	1.334905

65	1	0	6.336776	2.193638	2.141578
66	1	0	6.095245	3.917285	1.788183
67	1	0	7.104614	2.910235	0.726191
68	8	0	0.081008	2.881993	-1.694678
69	1	0	0.869814	3.420733	-1.934505
70	8	0	2.526188	3.910181	-1.563021
71	1	0	3.300643	3.875490	-2.147665

Conformer 2-2

Center	Atomic	Atomic	Coordinates (Angstroms)		
Number	Number	Type	X	Y	Z
1	6	0	3.040552	0.743409	-1.227193
2	6	0	3.047005	1.410430	0.227516
3	8	0	3.897698	0.604459	1.053851
4	6	0	3.158883	-0.610532	1.196927
5	6	0	3.113215	-1.279945	-0.256411
6	8	0	3.860971	-0.422497	-1.132949
7	6	0	1.770324	-0.113468	1.718987
8	6	0	0.599406	-1.057309	1.397115
9	6	0	0.550111	-1.582191	0.025569
10	6	0	1.695407	-1.244351	-0.901221
11	6	0	1.658518	0.159542	-1.663479
12	6	0	0.438263	0.993318	-1.251888
13	6	0	0.474026	1.581030	0.096598
14	6	0	1.673810	1.289094	0.961255
15	1	0	1.701309	-1.991394	-1.695347

16	6	0	3.739859	-2.666158	-0.302242
17	1	0	3.719856	-3.037269	-1.331709
18	1	0	3.192166	-3.363214	0.338360
19	1	0	4.785681	-2.648132	0.022989
20	8	0	3.552833	1.576372	-2.220054
21	1	0	4.503253	1.693356	-2.057894
22	6	0	1.629463	-0.085773	-3.180017
23	1	0	0.740812	-0.665249	-3.440360
24	1	0	2.522189	-0.640678	-3.482352
25	1	0	1.601464	0.854615	-3.735272
26	1	0	1.687133	2.033771	1.757191
27	6	0	3.587323	2.833108	0.236483
28	1	0	3.607644	3.205460	1.265665
29	1	0	2.960000	3.492825	-0.369688
30	1	0	4.609772	2.878218	-0.153131
31	8	0	3.779418	-1.406446	2.156007
32	1	0	4.717039	-1.494923	1.919205
33	6	0	1.824562	0.139403	3.233683
34	1	0	2.691997	0.760467	3.475600
35	1	0	1.900553	-0.796516	3.790689
36	1	0	0.913923	0.652645	3.553323
37	8	0	-0.223451	-1.311681	2.279776
38	8	0	-0.508645	1.109285	-2.033052
39	6	0	-0.528649	2.392742	0.575717
40	6	0	-1.687579	2.860168	-0.169355

41	1	0	-1.621187	2.763419	-1.246296
42	6	0	-2.816224	3.360327	0.395647
43	1	0	-2.944218	3.366943	1.480683
44	6	0	-3.941001	3.862338	-0.368436
45	1	0	-3.839398	3.850022	-1.454415
46	6	0	-5.078016	4.332014	0.184522
47	1	0	-5.164819	4.337798	1.273131
48	6	0	-6.255732	4.858102	-0.578123
49	1	0	-7.163502	4.282440	-0.348991
50	1	0	-6.089047	4.817279	-1.659915
51	1	0	-6.470506	5.900391	-0.303524
52	8	0	-0.414800	2.804303	1.876301
53	1	0	-1.020095	3.545541	2.035608
54	6	0	-0.490135	-2.332673	-0.457571
55	6	0	-1.727612	-2.758281	0.296022
56	1	0	-1.664159	-3.835456	0.511996
57	1	0	-1.751966	-2.243503	1.255807
58	6	0	-3.038410	-2.513635	-0.475802
59	1	0	-3.044066	-1.473164	-0.834328
60	6	0	-4.246861	-2.741289	0.397305
61	1	0	-4.402727	-1.974469	1.158151
62	6	0	-5.086191	-3.779064	0.312618
63	1	0	-4.901211	-4.540951	-0.445140
64	6	0	-6.279583	-3.994819	1.200162
65	1	0	-6.390043	-3.190039	1.935230

66	1	0	-6.195418	-4.944995	1.744897
67	1	0	-7.205963	-4.050687	0.612281
68	8	0	-0.422254	-2.753220	-1.741465
69	1	0	-1.276427	-3.177040	-1.984876
70	8	0	-3.013488	-3.395346	-1.625607
71	1	0	-3.724691	-3.142571	-2.236356

Conformer 2-3

Center	Atomic	Atomic	Coordinates (Angstroms)		
Number	Number	Type	X	Y	Z
1	6	0	0.146793	-2.903442	1.185727
2	6	0	-0.327832	-3.121557	-0.327202
3	8	0	0.843965	-3.446782	-1.089370
4	6	0	1.579191	-2.221774	-1.095738
5	6	0	2.034636	-1.964983	0.416355
6	8	0	1.564739	-3.080532	1.187406
7	6	0	0.534706	-1.192062	-1.635142
8	6	0	0.777528	0.261470	-1.194168
9	6	0	1.138909	0.458948	0.218316
10	6	0	1.288424	-0.770299	1.085403
11	6	0	-0.021620	-1.440163	1.706608
12	6	0	-1.307451	-0.737340	1.247075
13	6	0	-1.683556	-0.923793	-0.163557
14	6	0	-0.804798	-1.804198	-1.015695
15	1	0	1.887785	-0.483773	1.949663
16	6	0	3.544407	-1.876709	0.586053

17	1	0	3.950868	-1.030683	0.024606
18	1	0	4.044077	-2.788636	0.241998
19	1	0	3.780224	-1.748476	1.647303
20	8	0	-0.421120	-3.807976	2.080369
21	1	0	-0.072772	-4.692471	1.881522
22	6	0	0.065598	-1.398840	3.240035
23	1	0	0.952970	-1.942814	3.576386
24	1	0	-0.814665	-1.852566	3.700489
25	1	0	0.130246	-0.361078	3.575701
26	1	0	-1.393990	-2.109082	-1.880648
27	6	0	-1.328964	-4.256131	-0.487892
28	1	0	-2.250975	-4.042766	0.060432
29	1	0	-0.924595	-5.206298	-0.122931
30	1	0	-1.565427	-4.381294	-1.549346
31	8	0	2.647125	-2.342306	-1.983018
32	1	0	3.123565	-3.162671	-1.774957
33	6	0	0.455624	-1.258408	-3.168111
34	1	0	1.356195	-0.849391	-3.630945
35	1	0	-0.401166	-0.676932	-3.517225
36	1	0	0.341650	-2.298015	-3.488907
37	8	0	0.642819	1.164440	-2.022496
38	8	0	-1.940424	-0.050945	2.050855
39	6	0	-2.796985	-0.337599	-0.722026
40	6	0	-3.782933	0.475025	-0.026636
41	1	0	-3.757734	0.413301	1.054549

42	6	0	-4.667688	1.303831	-0.637768
43	1	0	-4.640812	1.456538	-1.719388
44	6	0	-5.660379	2.083481	0.074138
45	1	0	-5.692622	1.963555	1.157709
46	6	0	-6.521317	2.931711	-0.524585
47	1	0	-6.474715	3.041850	-1.610118
48	6	0	-7.555060	3.754706	0.181999
49	1	0	-7.399144	4.826769	-0.003289
50	1	0	-7.534818	3.588819	1.264465
51	1	0	-8.564864	3.519201	-0.182459
52	8	0	-2.984247	-0.539758	-2.062948
53	1	0	-3.886517	-0.280708	-2.306825
54	6	0	1.344583	1.693177	0.777676
55	6	0	1.213780	3.034311	0.094495
56	1	0	1.090082	2.880716	-0.976767
57	1	0	0.295677	3.511985	0.469018
58	6	0	2.377724	4.008098	0.346709
59	1	0	2.160310	4.934501	-0.200451
60	6	0	3.726910	3.489003	-0.063004
61	1	0	4.028406	2.530621	0.362838
62	6	0	4.546564	4.136627	-0.901023
63	1	0	4.217168	5.089449	-1.322237
64	6	0	5.896709	3.651773	-1.343291
65	1	0	6.677748	4.385091	-1.101164
66	1	0	5.922132	3.511338	-2.432273

67	1	0	6.159505	2.699446	-0.870780
68	8	0	1.656098	1.748586	2.094308
69	1	0	1.882060	2.676961	2.330112
70	8	0	2.354153	4.314883	1.776216
71	1	0	3.188274	4.751451	2.014834

Conformer 2-4

Center	Atomic	Atomic	Coordinates (Angstroms)		
Number	Number	Type	X	Y	Z
1	6	0	0.080823	-2.889110	1.198749
2	6	0	-0.392557	-3.097489	-0.316484
3	8	0	0.774422	-3.463141	-1.068601
4	6	0	1.546219	-2.260792	-1.078552
5	6	0	1.995003	-2.001787	0.434680
6	8	0	1.493626	-3.100538	1.209445
7	6	0	0.537886	-1.205978	-1.635854
8	6	0	0.814106	0.243261	-1.203986
9	6	0	1.158071	0.442057	0.212807
10	6	0	1.270535	-0.784585	1.089580
11	6	0	-0.055587	-1.419411	1.711860
12	6	0	-1.338317	-0.713085	1.248044
13	6	0	-1.670729	-0.844050	-0.179379
14	6	0	-0.823129	-1.770923	-1.016773
15	1	0	1.872155	-0.505000	1.954537
16	6	0	3.505097	-1.946548	0.615460
17	1	0	3.935461	-1.114548	0.050783

18	1	0	3.986199	-2.871962	0.281511
19	1	0	3.735692	-1.815606	1.677536
20	8	0	-0.513882	-3.775809	2.092771
21	1	0	-0.187166	-4.669640	1.899036
22	6	0	0.030386	-1.372408	3.245069
23	1	0	0.909099	-1.928169	3.585071
24	1	0	-0.857380	-1.809260	3.706628
25	1	0	0.111492	-0.334076	3.576212
26	1	0	-1.416884	-2.065664	-1.881782
27	6	0	-1.427598	-4.201370	-0.477204
28	1	0	-2.345888	-3.956282	0.064116
29	1	0	-1.055249	-5.161389	-0.104311
30	1	0	-1.661738	-4.324992	-1.539308
31	8	0	2.618199	-2.422471	-1.954591
32	1	0	3.059135	-3.261466	-1.742558
33	6	0	0.468731	-1.285512	-3.168581
34	1	0	1.385115	-0.908759	-3.627912
35	1	0	-0.367551	-0.682979	-3.530659
36	1	0	0.326122	-2.324417	-3.479935
37	8	0	0.713793	1.143407	-2.040631
38	8	0	-2.005840	-0.074046	2.062978
39	6	0	-2.699900	-0.149767	-0.774803
40	6	0	-3.579286	0.805944	-0.121349
41	1	0	-3.267027	1.118002	0.867951
42	6	0	-4.741211	1.276789	-0.641538

43	1	0	-5.123685	0.910562	-1.597501
44	6	0	-5.585277	2.244080	0.030559
45	1	0	-5.235329	2.610096	0.996576
46	6	0	-6.754406	2.698063	-0.465670
47	1	0	-7.092606	2.320287	-1.432973
48	6	0	-7.649498	3.693740	0.207139
49	1	0	-7.241134	4.020211	1.169580
50	1	0	-8.647690	3.269574	0.384994
51	1	0	-7.796103	4.582456	-0.422702
52	8	0	-2.900632	-0.388227	-2.107255
53	1	0	-3.482559	0.292229	-2.478595
54	6	0	1.381604	1.675221	0.767524
55	6	0	1.287930	3.014618	0.074547
56	1	0	1.176218	2.856937	-0.997425
57	1	0	0.374750	3.513487	0.433450
58	6	0	2.467037	3.967444	0.336631
59	1	0	2.272604	4.895704	-0.215997
60	6	0	3.810624	3.422504	-0.057375
61	1	0	4.091106	2.460542	0.374659
62	6	0	4.648997	4.052377	-0.890452
63	1	0	4.339902	5.009146	-1.318089
64	6	0	5.994271	3.542606	-1.319065
65	1	0	6.236478	2.587431	-0.841314
66	1	0	6.785719	4.263144	-1.072470
67	1	0	6.026750	3.398192	-2.407337

68	8	0	1.672695	1.732728	2.088985
69	1	0	1.919446	2.656709	2.321426
70	8	0	2.433043	4.279649	1.764634
71	1	0	3.272228	4.701756	2.011420

Conformer 2-5

Center	Atomic	Atomic	Coordinates (Angstroms)		
Number	Number	Type	X	Y	Z
1	6	0	0.469169	-2.732349	1.295952
2	6	0	-0.043263	-3.095455	-0.176298
3	8	0	1.121943	-3.360327	-0.970426
4	6	0	1.734185	-2.075417	-1.090134
5	6	0	2.222132	-1.671961	0.379370
6	8	0	1.896710	-2.772040	1.242132
7	6	0	0.573752	-1.187925	-1.646297
8	6	0	0.702967	0.307852	-1.315210
9	6	0	1.077052	0.634585	0.069341
10	6	0	1.388877	-0.511627	1.004577
11	6	0	0.178942	-1.260250	1.730236
12	6	0	-1.186305	-0.714783	1.288920
13	6	0	-1.598578	-1.029383	-0.088455
14	6	0	-0.673549	-1.877627	-0.924275
15	1	0	1.992247	-0.107638	1.817411
16	6	0	3.720743	-1.424621	0.470475
17	1	0	4.012369	-0.577039	-0.156235
18	1	0	4.296209	-2.301347	0.154686

19	1	0	3.986848	-1.208508	1.510054
20	8	0	0.030871	-3.625957	2.270894
21	1	0	0.465397	-4.480793	2.116707
22	6	0	0.326201	-1.108248	3.252207
23	1	0	-0.484919	-1.612642	3.781914
24	1	0	0.302830	-0.049296	3.520487
25	1	0	1.276115	-1.541937	3.577983
26	1	0	-1.266219	-2.294485	-1.738561
27	6	0	-0.935982	-4.327070	-0.218556
28	1	0	-1.848178	-4.168209	0.363543
29	1	0	-0.424197	-5.209694	0.179235
30	1	0	-1.207607	-4.540430	-1.257302
31	8	0	2.771759	-2.156203	-2.016765
32	1	0	3.336528	-2.908456	-1.775098
33	6	0	0.438759	-1.369409	-3.166042
34	1	0	1.278309	-0.915715	-3.696696
35	1	0	-0.481176	-0.892184	-3.512949
36	1	0	0.407252	-2.434974	-3.411794
37	8	0	0.487223	1.135007	-2.204295
38	8	0	-1.849895	-0.038776	2.076664
39	6	0	-2.786107	-0.590775	-0.629472
40	6	0	-3.819499	0.164194	0.061618
41	1	0	-3.743099	0.174789	1.142086
42	6	0	-4.809607	0.860426	-0.553367
43	1	0	-4.845639	0.946108	-1.642048

44	6	0	-5.844500	1.580107	0.161619
45	1	0	-5.815751	1.524010	1.250489
46	6	0	-6.813924	2.300498	-0.438820
47	1	0	-6.828482	2.348657	-1.529754
48	6	0	-7.893015	3.057595	0.273607
49	1	0	-7.807557	2.957559	1.360967
50	1	0	-8.889076	2.702999	-0.026486
51	1	0	-7.854740	4.127242	0.023874
52	8	0	-3.004907	-0.895546	-1.946103
53	1	0	-3.938598	-0.747672	-2.163166
54	6	0	1.164619	1.917378	0.542480
55	6	0	0.832889	3.184772	-0.206605
56	1	0	0.621368	2.939774	-1.246790
57	1	0	-0.082507	3.606688	0.234296
58	6	0	1.921752	4.275244	-0.145753
59	1	0	1.537311	5.130529	-0.721720
60	6	0	3.232443	3.839994	-0.753280
61	1	0	3.186008	3.675646	-1.831017
62	6	0	4.385541	3.640933	-0.105134
63	1	0	4.422622	3.778162	0.976912
64	6	0	5.671291	3.209474	-0.753454
65	1	0	5.551879	3.067659	-1.832904
66	1	0	6.029666	2.265019	-0.322531
67	1	0	6.465300	3.951110	-0.591144
68	8	0	1.521442	2.095105	1.836433

69	1	0	1.693563	3.048695	1.991680
70	8	0	2.032098	4.678118	1.236887
71	1	0	2.776061	5.294401	1.335024

Conformer 2-6

Center	Atomic	Atomic	Coordinates (Angstroms)		
Number	Number	Type	X	Y	Z
1	6	0	0.371641	-2.716926	1.311779
2	6	0	-0.148373	-3.069253	-0.161093
3	8	0	1.011402	-3.382027	-0.946756
4	6	0	1.669867	-2.120586	-1.073086
5	6	0	2.160753	-1.719645	0.395459
6	8	0	1.797169	-2.802088	1.264390
7	6	0	0.545913	-1.198595	-1.645445
8	6	0	0.715783	0.294460	-1.324179
9	6	0	1.095712	0.619689	0.059405
10	6	0	1.359057	-0.528707	1.006918
11	6	0	0.125540	-1.233075	1.735427
12	6	0	-1.234498	-0.674182	1.293236
13	6	0	-1.616972	-0.930048	-0.103993
14	6	0	-0.729131	-1.835750	-0.922503
15	1	0	1.971689	-0.137917	1.819176
16	6	0	3.665419	-1.515568	0.493534
17	1	0	4.217148	-2.409844	0.185031
18	1	0	3.931796	-1.301542	1.533506
19	1	0	3.984629	-0.679919	-0.135921

20	8	0	-0.098821	-3.589678	2.289482
21	1	0	0.301727	-4.461350	2.137783
22	6	0	0.276346	-1.074956	3.256313
23	1	0	0.278929	-0.013700	3.517695
24	1	0	1.215997	-1.528567	3.584846
25	1	0	-0.546650	-1.555256	3.789381
26	1	0	-1.333022	-2.239712	-1.734754
27	6	0	-1.085797	-4.267421	-0.198807
28	1	0	-1.993299	-4.071245	0.379270
29	1	0	-0.608294	-5.165981	0.205887
30	1	0	-1.362377	-4.476927	-1.237005
31	8	0	2.710713	-2.247242	-1.991090
32	1	0	3.242035	-3.021310	-1.742495
33	6	0	0.415411	-1.390228	-3.164336
34	1	0	0.348728	-2.456485	-3.399453
35	1	0	1.274413	-0.971721	-3.692993
36	1	0	-0.484837	-0.885677	-3.523010
37	8	0	0.517670	1.121988	-2.217412
38	8	0	-1.920964	-0.041648	2.097463
39	6	0	-2.731802	-0.370846	-0.686192
40	6	0	-3.654626	0.558614	-0.054585
41	1	0	-3.309236	0.994782	0.874982
42	6	0	-4.890044	0.864836	-0.526178
43	1	0	-5.297334	0.363291	-1.407419
44	6	0	-5.777916	1.816852	0.110371

45	1	0	-5.400412	2.316813	1.003211
46	6	0	-7.018059	2.104428	-0.335181
47	1	0	-7.382641	1.593722	-1.229001
48	6	0	-7.959232	3.081153	0.301649
49	1	0	-8.893998	2.588411	0.603904
50	1	0	-8.239930	3.876641	-0.402898
51	1	0	-7.518204	3.549510	1.188168
52	8	0	-2.981657	-0.724756	-1.984452
53	1	0	-3.634372	-0.119490	-2.368544
54	6	0	1.240515	1.902646	0.518081
55	6	0	0.988794	3.175884	-0.252643
56	1	0	0.783732	2.927915	-1.293476
57	1	0	0.088158	3.651406	0.163467
58	6	0	2.130653	4.210403	-0.182763
59	1	0	1.801826	5.076269	-0.777036
60	6	0	3.429831	3.701785	-0.757481
61	1	0	3.398505	3.529323	-1.834505
62	6	0	4.556706	3.450091	-0.081961
63	1	0	4.577257	3.597126	0.999222
64	6	0	5.831233	2.942661	-0.696512
65	1	0	5.729742	2.799221	-1.777563
66	1	0	6.124789	1.982814	-0.250795
67	1	0	6.661782	3.639483	-0.519594
68	8	0	1.588495	2.078924	1.814965
69	1	0	1.810523	3.023831	1.960247

70	8	0	2.233463	4.625786	1.196737
71	1	0	3.005108	5.205715	1.302652

Conformer 2-7

Center	Atomic	Atomic	Coordinates (Angstroms)		
Number	Number	Type	X	Y	Z
1	6	0	3.017717	0.981135	-1.106628
2	6	0	2.915935	1.553166	0.385271
3	8	0	3.774247	0.744519	1.201579
4	6	0	3.101400	-0.515514	1.235162
5	6	0	3.153704	-1.092321	-0.257508
6	8	0	3.894526	-0.145235	-1.042062
7	6	0	1.665781	-0.130949	1.721941
8	6	0	0.575258	-1.123294	1.288222
9	6	0	0.600495	-1.536818	-0.118348
10	6	0	1.765815	-1.086658	-0.968088
11	6	0	1.691595	0.357527	-1.646908
12	6	0	0.413332	1.107550	-1.248592
13	6	0	0.344856	1.587681	0.141282
14	6	0	1.520138	1.307634	1.043040
15	1	0	1.846583	-1.782933	-1.803228
16	6	0	3.851890	-2.440992	-0.357788
17	1	0	3.313165	-3.201616	0.214576
18	1	0	4.880310	-2.393380	0.015903
19	1	0	3.894452	-2.748234	-1.407493
20	8	0	3.532286	1.900744	-2.018259

21	1	0	4.467792	2.050377	-1.804308
22	6	0	1.746217	0.205685	-3.174774
23	1	0	2.678708	-0.286552	-3.465787
24	1	0	1.696673	1.176140	-3.673679
25	1	0	0.900280	-0.397244	-3.512986
26	1	0	1.453983	2.000965	1.881615
27	6	0	3.367527	3.000820	0.508574
28	1	0	2.729482	3.659965	-0.086985
29	1	0	4.402174	3.131141	0.174213
30	1	0	3.316041	3.306667	1.558393
31	8	0	3.727251	-1.335840	2.170352
32	1	0	4.678597	-1.349357	1.975629
33	6	0	1.639313	0.028902	3.249489
34	1	0	1.747185	-0.934266	3.751969
35	1	0	0.686233	0.467700	3.556449
36	1	0	2.456296	0.682429	3.569054
37	8	0	-0.247328	-1.516790	2.120297
38	8	0	-0.489047	1.248231	-2.076106
39	6	0	-0.731088	2.294303	0.628469
40	6	0	-1.884648	2.741568	-0.136553
41	1	0	-1.765091	2.724412	-1.213052
42	6	0	-3.066996	3.130805	0.405474
43	1	0	-3.243097	3.057464	1.481250
44	6	0	-4.187245	3.610871	-0.378877
45	1	0	-4.038043	3.672009	-1.457630

46	6	0	-5.375127	3.974181	0.146552
47	1	0	-5.509796	3.906804	1.228229
48	6	0	-6.549879	4.472357	-0.638975
49	1	0	-6.333062	4.510049	-1.711921
50	1	0	-6.844711	5.479727	-0.313073
51	1	0	-7.426970	3.827257	-0.488945
52	8	0	-0.704439	2.607212	1.961427
53	1	0	-1.365492	3.292776	2.146251
54	6	0	-0.388427	-2.294320	-0.692464
55	6	0	-1.632950	-2.802958	0.000935
56	1	0	-1.439330	-3.824501	0.365025
57	1	0	-1.795287	-2.198665	0.893501
58	6	0	-2.888232	-2.808350	-0.882382
59	1	0	-3.021564	-1.814654	-1.326778
60	6	0	-4.161975	-3.214821	-0.189341
61	1	0	-5.031251	-3.232205	-0.849471
62	6	0	-4.309878	-3.546228	1.099793
63	1	0	-3.449172	-3.517414	1.767745
64	6	0	-5.612282	-3.945366	1.733135
65	1	0	-5.538248	-4.941496	2.189834
66	1	0	-6.430907	-3.960516	1.005376
67	1	0	-5.882536	-3.250296	2.539595
68	8	0	-0.251078	-2.626534	-1.994296
69	1	0	-1.045162	-3.131704	-2.290684
70	8	0	-2.695923	-3.662174	-2.056844

71	1	0	-2.805682	-4.587479	-1.777966

42	6	0	-4.503537	-0.310927	-0.654109
43	1	0	-4.332334	-0.191304	-1.724301
44	6	0	-5.872983	-0.402691	-0.196481
45	1	0	-6.055399	-0.518613	0.869563
46	6	0	-6.922735	-0.349332	-1.039317
47	1	0	-6.752979	-0.232795	-2.110296
48	8	0	-1.813951	-0.122678	-1.590539
49	6	0	-8.360287	-0.437662	-0.635962
50	1	0	-8.827902	-1.287504	-1.162845
51	1	0	-8.883279	0.473381	-0.974830
52	8	0	-8.469335	-0.591936	0.773189
53	1	0	-9.405906	-0.635707	1.015624

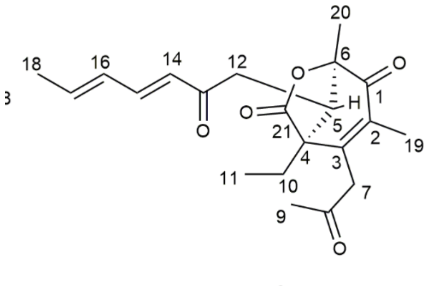

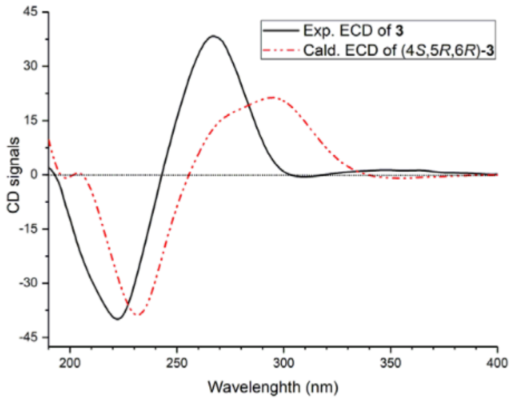

Figure S41 Calculated and experimental ECD spectra of trichodermolide B

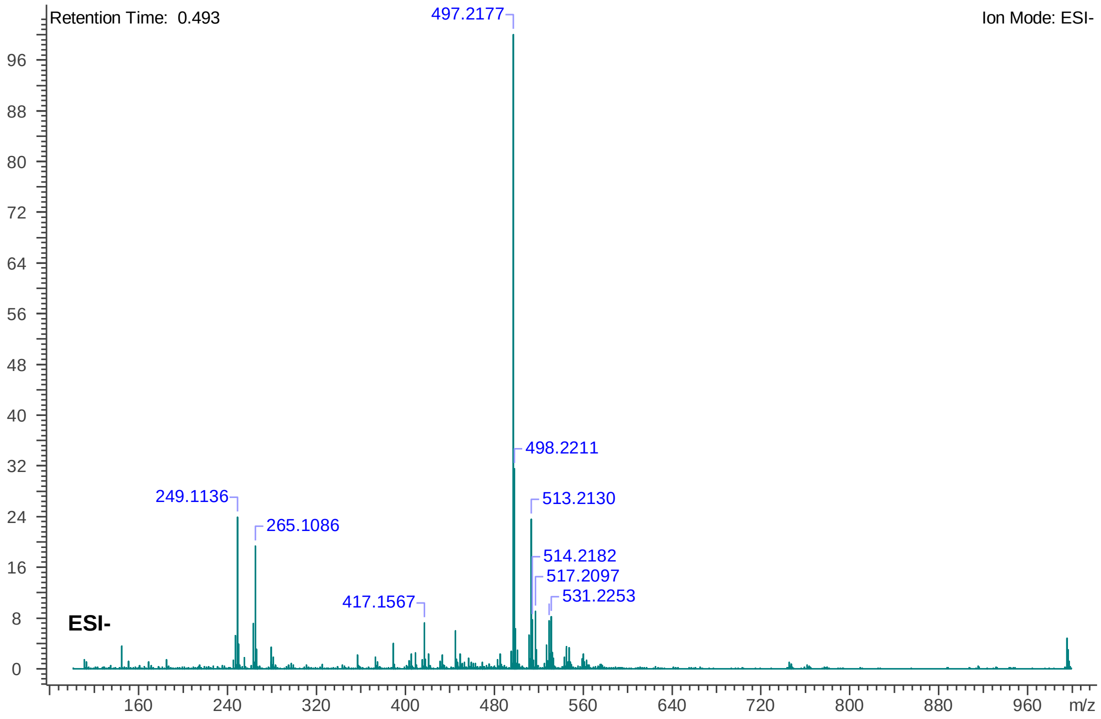

Figure S42 HR-ESI-MS spectra of compound **11**
